# Production and molecular characterization of bread wheat lines with reduced amount of α-type gliadins

**DOI:** 10.1186/s12870-017-1211-3

**Published:** 2017-12-19

**Authors:** Francesco Camerlengo, Francesco Sestili, Marco Silvestri, Giuseppe Colaprico, Benedetta Margiotta, Roberto Ruggeri, Roberta Lupi, Stefania Masci, Domenico Lafiandra

**Affiliations:** 1 Department of Agriculture and Forestry Sciences, University of Tuscia, 01100 Viterbo, Italy; 2 Institute of Biosciences and Bioresources, CNR, 70126 Bari, Italy; 3 Barilla G. e R. F.lli S.p.A, 43122 Parma, Italy

**Keywords:** Gluten, α-gliadins, Celiac disease, Bread wheat, Deletion lines

## Abstract

**Background:**

Among wheat gluten proteins, the α-type gliadins are the major responsible for celiac disease, an autoimmune disorder that affects about 1% of the world population. In fact, these proteins contain several toxic and immunogenic epitopes that trigger the onset of the disease. The α-type gliadins are a multigene family, encoded by genes located at the complex *Gli-2* loci.

**Results:**

Here, three bread wheat deletion lines (Gli-A2, Gli-D2 and Gli-A2/Gli-D2) at the *Gli-2* loci were generated by the introgression in the bread wheat cultivar Pegaso of natural mutations, detected in different bread wheat cultivars. The molecular characterization of these lines allowed the isolation of 49 unique expressed genes coding α-type gliadins, that were assigned to each of the three *Gli-2* loci.

The number and the amount of α-type gliadin transcripts were drastically reduced in the deletion lines. In particular, the line Gli-A2/Gli-D2 contained only 12 active α-type gliadin genes (−75.6% respect to the cv. Pegaso) and a minor level of transcripts (−80% compared to cv. Pegaso). Compensatory pleiotropic effects were observed in the two other classes of gliadins (ω- and γ-gliadins) either at gene expression or protein levels.

Although the comparative analysis of the deduced amino acid sequences highlighted the typical structural features of α-type gliadin proteins, substantial differences were displayed among the 49 proteins for the presence of toxic and immunogenic epitopes.

**Conclusion:**

The deletion line Gli-A2/Gli-D2 did not contain the 33-mer peptide, one of the major epitopes triggering the celiac disease, representing an interesting material to develop less “toxic” wheat varieties.

**Electronic supplementary material:**

The online version of this article (10.1186/s12870-017-1211-3) contains supplementary material, which is available to authorized users.

## Background

With a surface of 220 M of hectares, wheat represents the most widely cultivated crop and a major component of the human diet. This is the result of its agronomic adaptability, ease of storage, nutritional value and ability of its flour to produce a large variety of palatable and satisfying foods [1]. It is firmly established that functional properties of doughs, necessary to realize different types of end products are mainly related to gluten proteins. The main constituents of the gluten are gliadins and glutenins that represent the storage proteins, accumulated in the endosperm during the graining phase and used by the growing embryo during the first phases of germination. Gliadins and glutenins constitute about 80% of the total proteins and are classified as “prolamins” because of their high content in proline and glutamine [2]. Glutenins are polymeric proteins, whose constituents, high and low molecular weight glutenin subunits (HMW-GS and LMW-GS, respectively), are held together by intermolecular disulfide bonds [3, 4]. Genes encoding these subunits are present at the *Glu-1* and *Glu-3* loci, present on the long and short arm of the homeologous group 1 chromosomes, respectively [4]. Differently, gliadins are monomeric proteins, classified in four groups, known as α-, β-, γ- and ω-gliadins, on the basis of their decreasing mobility in electrophoresis at acid pH [5]. Subsequent comparisons of amino acid and DNA sequences have demonstrated that the separation of gliadins in four groups is not correct as α- and β-gliadins share similar sequences leading Kasarda et al. [6] to suggest that, from a structural point of view, gliadins could be subdivided into three groups: α-, γ- and ω-type.

The genes encoding ω- and γ-type gliadins are located at three complex *Gli-1* loci (*Gli-A1*, *Gli-B1* e *Gli-D1*) present on the short arms of the homeologous chromosomes of group 1, whereas α-type are controlled by genes located at the *Gli-2* loci (*Gli-A2*, *Gli-B2* e *Gli-D2)* on the short arms of the homeologous chromosomes of group 6 [7, 8].

Within the same *Gli-1* and *Gli-2* loci, genes encoding for mutant form of gliadins are also present [9, 10]. They are highly similar in sequence to individual α-, γ- and ω-type gliadins, and are probably derived from these components by point mutations resulting in the presence of an additional cysteine residue. The extra cysteine residue is capable to form intermolecular disulfide bonds resulting in incorporation of the modified gliadin into the glutenin polymers where it can act as chain terminator and limiting their molecular weight distribution [3, 11].

It is estimated that the average number of copies of α-gliadin genes per haploid genome ranges from 25–35 to 100–150 copies; this large number has been generated by duplication, deletion events and retrotransposon insertion [12–14].

At least half of α-type gliadin genes are considered inactive or pseudogenes because they show premature stop codons in the coding sequences [15–17]. From a quantitative point of view the proportions of the different classes of gliadins fluctuate considerably among different varieties, although the α-type gliadins are generally present in larger amount, followed by the γ- and ω-type gliadins [18].

Several studies have shown that peptides derived from incomplete digestion of all gluten proteins, but in particular those of the α-type gliadins, are the main responsible for the onset of celiac disease [17, 19, 20]. Celiac disease is a permanent food disorder caused by an abnormal autoimmune response to dietary gluten peptides that occurs in genetically susceptible individuals carrying T-cell with HLA-DQ2 or -DQ8 receptors [21–23]. The autoimmune component is demonstrated by serologic autoantibodies such as serum anti-tissue transglutaminase (tTG) and anti-endomysial antibodies (EMA) [24]. The partially digested peptides contain epitopes that are involved in the disease processes in different manner and they are accordingly classified into two groups: toxic and immunogenic epitopes. Toxic peptides can induce mucosal damage when added in culture to duodenal endoscopic biopsy or if administered in vivo, whereas the peptides defined immunogenic stimulate HLA-DQ2- or DQ8-restricted T cell clones isolated from jejunal mucosa or peripheral blood of coeliac patients [25]. However, it is not excluded that some peptides can perform both functions, toxic and immunogenic.

Several studies have demonstrated that the large genetic diversity existing at the different loci encoding gliadin components is reflected in a large variation in the number of T-cell–stimulatory peptides present in different durum and bread wheat accessions as well as in diploid progenitors of polyploid wheats [17, 26–28]. In the past years, several lines and cultivars have been identified in durum and bread wheat, characterized by the deletion of genes associated to the *Gli-1, Gli-2* and *Glu-1* loci [29, 30].

In this paper, we report the production of bread wheat lines with reduced amount of α-type gliadins, and the characterization of their corresponding genes, with the aim to produce wheat varieties with a reduced content in immunogenic and toxic peptides.

## Methods

### Plant material and experimental site

Bread wheat lines lacking gliadin components at the three homeologous *Gli-1* loci and two lines without the gliadins associated at the *Gli-A2* and *Gli-D2* loci, previously identified in different bread wheat cultivars, have been initially used to develop the lines used in this work (Table 1). In order to have the single deletion lines in the same genetic background, they were crossed and backcrossed four times with the Italian bread wheat cultivar Pegaso. The gliadins present in the seeds obtained after each backcross were analyzed by one dimensional polyacrylamide gel electrophoresis in acidic conditions (A-PAGE) and those lacking the gliadins, associated at one of the five above mentioned loci, were used for the next step of backcrossing. BC_4_F_6_ seeds were used for all the analyses. The single deletion lines obtained from the backcross with the bread wheat cv Pegaso were crossed between them, thus, besides single Gli-A2, or Gli-D2 deletion lines, also double Gli-A2/Gli-D2 deletion lines were isolated.

**Table 1:** List of deletion mutant lines at the *Gli-1* and *Gli-2* loci

Locus	Cultivar	Country	Reference
*Gli-A1*	Saratovskaja 29	USSR	Redaelli et al. [30]
*Gli-B1*	Spada	Italy	Lafiandra et al. [31]
*Gli-D1*	Darius	France	Branlard et al. [32]
*Gli-A2*	Reader	USA	Lafiandra et al. [29]
*Gli-D2*	Saratovskaja 29	USSR	Redaelli et al. [30]

The three BC_4_F_6_ deletion lines and the cultivar Pegaso were grown in open field during 2014/2015 growing season at the Experimental Farm of the University of Tuscia, located in Viterbo, Italy (lat. 42°26’ N, long. 12° 04′ E, altitude 310 m a.s.l.). The experimental site is characterized by a Mediterranean climate with mean annual maximum and minimum temperatures of 19 and 8 °C, respectively, and annual rainfall of 743 mm.

### Electrophoretic analysis of gliadins

Monomeric gliadins were extracted from single kernel and fractionated by A-PAGE as described by Pflüger et al. [33]. Two-dimensional (two-pH) polyacrylamide gel electrophoresis was carried out as described by Lafiandra and Kasarda [34].

### Isolation of genes coding α-type gliadin proteins expressed in grain

Genes coding α-type gliadins were amplified by Reverse transcriptase-PCR. Total RNA was extracted from immature kernels (24 Day Post Anthesis, DPA) using the Spectrum Plant Total RNA kit (Sigma-Aldrich, St. Louis, USA) and following manufacturer’s instructions. One μg of RNA constituted the template for the synthesis of cDNA, based on the QuantiTect Reverse Transcription Kit (Qiagen, Hilden, Germany). Each PCR reaction were performed in a volume of 20 μL containing 10 μL of GoTaq Green Master Mix (Promega), 0.5 μM of each primer (L1 and R1, Table 2) and 1 μL of cDNA. PCR conditions were: an initial denaturation at 95 °C for 5 min, followed by 35 cycles at 95 °C for 1 min, 61 °C for 30 s,72 °C for 1 min and a final extension at 72 °C for 5 min. PCR amplicons were checked on 1% agarose gel electrophoresis.

**Table 2:** List of primer pairs used to isolate the α-type gliadins and in RT-PCR analysis

Primer name	Sequence	Reference
L1	5’-ATGAAGACCTTTCTCATCCTTG	Xie et al. [35]
R1	5’-TCAGTTRGTACCRAAGATGCC	
RT a-gli F	5’-AGACCTTTCTCATCCTTGCC	Li et al. [36]
RT a-gli R	5’-TGTACCAATGGAACTTGCTCT	
G1Gli*1	5’-ACAGGTGAACCCATGCAAGAATTT	Piston et al. [37]
G1Gli*2	5’-TGCATGATGATGGAATGTATGATGG	
G2Gli*1	5’-TCATTCCCCCAACAACAACGG	Piston et al. [37]
G2Gli*2	5’-AGGTTTGCATTGTTGCAAGAGGAT	
G3 Gli*1	5’-GCAAATCCTGGTGCCACTGTCTCAA	Piston et al. [37]
G3 Gli*2	5’-TGGGACATACACGTTGCACATGGTT	
G4 Gli*1	5’-GATCCTGCGGCCACTATTTCAGCTC	Piston et al. [37]
G4 Gli*2	5’-CAGGTGGCACATACACGTTGCACAT	
QF18	5’-AAGGCAAGCAAGCAGTAG	Altenbach et al. [38]
QR18	5’-GATTGTTGAGGTGATTGTAGC	
QF21	5’-CAACCACCACAACAATTC	Altenbach et al. [38]
QR21	5’-TTACATCTCTTCATTTCATAGG	

PCR amplicons were recovered from gel by NucleoSpin® Gel and PCR Clean-up kit (Macherey-Nagel, Duren, Germany) and the purified products were ligated into pGEM-T Easy vector (Promega, Madison, WI, USA). JM109 Competent Cells (Promega, Madison, WI, USA) were transformed with the ligation reaction. Transformed cells were plated onto LB medium plus Ampicillin containing X-Gal and IPTG (Sigma-Aldrich,) for blue/white screening.

White colonies were inoculated in LB medium plus Ampicillin, and plasmids were extracted with the NucleoSpin® Plasmid kit (Macherey-Nagel, Duren, Germany). The presence of the insert was verified by digesting about one μg of each recombinant plasmid for 2 h with 5 units of EcoRI restriction enzyme (Promega, Madison, WI, USA). Digested plasmids were checked on an agarose gel (0.8%).

DNA sequencing was performed with the universal primers T7 and SP6 by Eurofins Genomics (Ebersberg, Germany).

Full length sequences of α-gliadin genes were reconstructed by overlapping the fragments with the bioinformatic program Geneious R8. Clustal Omega (http://www.ebi.ac.uk/Tools/msa/clustalo/) was used to align and compare the deduced amino acid sequences.

### Phylogenetic analysis

The phylogenetic tree was generated by Neighbor Joining method using MEGA7 software package (http://www.megasoftware.net/) [39].

### Quantification of gliadin transcripts

Real-time PCR was performed on total RNA using primer pairs specific for α-, γ and ω-gliadins (Table 2). The analyses were performed in the CFX 96 Real-Time PCR Detection System device (Bio-Rad Hercules, CA, USA). The reactions were carried out in a final volume of 15 μL, consisting in 7.5 μL SsoAdvUniver SYBR GRN SMX (Bio-Rad Hercules, CA, USA), 0.5 μM of each primer and 1 μL of cDNA, following the protocol below: 94 °C for 30 s and 40 cycles at 94 °C for 5 s, 60 °C for 30 s and melt curve 65–95 °C with 0.5 °C increment 5 s/step. Three technical replicates per biological sample and three independent plants per genotype were analyzed. The quantification analysis was performed as described in Sestili et al. [40].

## Results

### Separation of gliadins by one- and two-dimensional electrophoresis

The utilization of the three different deletion lines, without the *Gli-1* loci (*Gli-A1, Gli-B1, Gli-D1*) and the two without the *Gli-2* loci (*Gli-A2* and *Gli-D2*), all backcrossed four times with the bread wheat cv Pegaso, have allowed the isolation of five single deletion lines. The five lines obtained differ for the absence of different clusters of gliadin components encoded by genes present at the *Gli-1* or *Gli-2* locus, whereas the remaining gliadin genes are the same as those present in Pegaso.

**Fig. 1:**
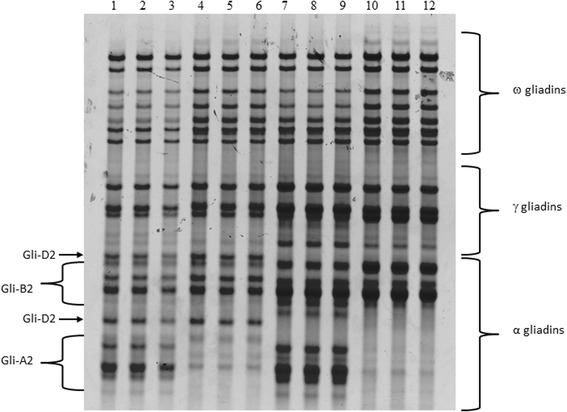
A-PAGE separation of gliadin fractions. Proteins were extracted from kernels of Pegaso (1–3), Gli-A2 deletion line (4–6); Gli-D2 deletion line (7–9) and Gli-A2/Gli-D2 double deletion line (10–12)

One dimensional electrophoretic separations of gliadin components present in the bread wheat cultivar Pegaso and derived single and double deletion lines (Gli-A2, Gli-D2 and Gli-A2/Gli-D2) are reported in Fig. 1. Three independent single seeds were analyzed for each deletion line. Comparison of the electrophoretic separations of gliadins present in Pegaso, with single and double deletion lines, allowed to assign gliadin components to the three different *Gli-2* loci. Although the gliadins were extracted from single kernels for each genotype and the same volume was loaded on the gel, an increase of intensity of the other gliadin fractions was observed in the deletion lines and this was more marked in the double deletion genotype (Fig. 1).

**Fig. 2:**
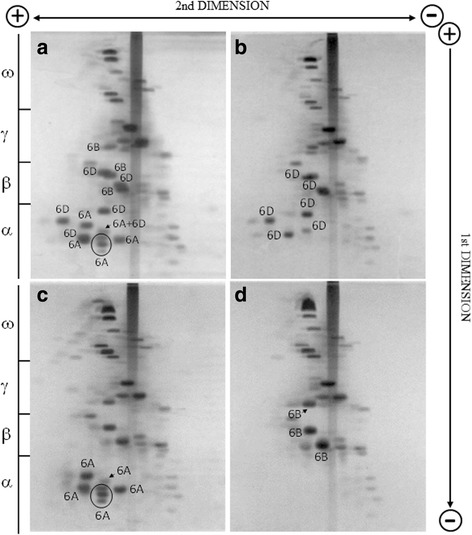
Two-dimensional (2-pH) electrophoretic separation of gliadins. Proteins were extracted from the bread wheat cultivar Pegaso (**a**) and Gli-A2 (**b**), Gli-D2 (**c**), Gli-A2/Gli-D2 (**d**) deletion lines

Two dimensional electrophoretic separations have a higher resolving power of a complex mixture of proteins as is the case for the gliadin fraction present in wheat. Using the two dimensional separation at two different pH [34] a more complete chromosomal assignment of the gliadins associated at the *Gli-2* loci was possible (Fig. 2). Though the deletion line involving the *Gli-B2* locus was not available, components associated at this locus were also identified, after assignment of gliadin components at the three *Gli-1* loci (data not shown), along with those associated at the *Gli-A2* and *Gli-D2* loci.

A total of seventeen gliadins were found associated to the *Gli-2* loci, seven to the *Gli-A2*, six to the *Gli-D2* and three to the *Gli-B2* locus. It is very likely that these numbers are an underestimation since it has been reported that single protein spots could derive from different genes because of their close similarity [7].

### Isolation and genetic localization of α-type gliadins expressed in the bread wheat cv Pegaso

The transcripts of α-type gliadin genes were isolated by Reverse transcriptase-PCR on immature caryopsis (24 DPA) of the bread wheat cv Pegaso and derived deletion lines at the *Gli-A2*, *Gli-D2* and *Gli-A2/Gli-D2* loci. Full-length ORFs were amplified using specific oligonucleotides (L1 and R1) designed on highly conserved regions previously described by Xie et al. [35]. The PCR products were checked on agarose gel (data not shown) and ranged from 800 to 950 base pairs. After purification, the amplicons were cloned into pGEM T-easy vector. In total, 381 sequences of α-type gliadins were identified and sequenced. In particular, 104 sequences were isolated from the bread wheat cv Pegaso; 101, 107 and 69 were isolated from the deletion lines Gli-A2, Gli-D2 and Gli-A2/Gli-D2, respectively. Several chimeric genes, probably produced during PCR amplification, and some pseudogenes were observed and discarded. Blast analysis on NCBI database highlighted that the sequences isolated from the deletion lines Gli-A2 and Gli-D2 had a high similarity with genes coding α-type gliadins from *Triticum aestivum, Triticum urartu, Aegilops speltoides* and *Aegilops tauschii* species; while the sequences deriving from the double deletion line and associated to the *Gli-B2* locus showed the higher similarity with genes coding α-type gliadins isolated from *Aegilops speltoides*; this is not unexpected, as it has been suggested that an unknown close relative of *Aegilops speltoides* donated the B genome to polyploid wheats [41].

After removing either redundant genes and chimeric/pseudogenic sequences, 49 unique genes were isolated from the cv Pegaso and submitted to the EMBL database (from the acc. n° LT627562 to LT627610). Considering together the data obtained by the sequencing of the four genotypes, all the full-length sequences were assigned to one of the homoeologous *Gli-2* loci: 18 sequences to the *Gli-A2*, 12 to the *Gli-B2* and 19 to the *Gli-D2*. Alignment of the isolated α-type-gliadins is reported in Additional file 1: Figure S1. All α-type gliadin genes isolated lack introns and their size range from 846 to 960 bp.

### Analysis of amino acidic sequences of the α-type gliadins isolated from the bread wheat cv Pegaso

The 49 deduced amino acidic sequences were aligned through the bioinformatic tool Clustal Omega (EMBL-EBI) (Aditional file 2: Figure S2). All the sequences had the typical features of α-type gliadins, characterized from a signal peptide (P) and five domains: 1) N-terminal domain (R1); 2) Polyglutamine domain 1 (QR1); 3) Unique domain 1 (NR1); 4) Polyglutamine domain 2 (QR2); 5) C-terminal unique domain (NR2). In Additional file 3: Table S1 the number of amino acids present in each domain of the 49 α-type gliadins isolated from the bread wheat cv Pegaso is reported. Size of the deduced proteins ranges from 281 to 319 amino acids, whereas the molecular weight ranges from 30.4 to 34.7 kDa. The signal peptide is strongly conserved in size (20 aa) and composition in all the sequences. Similarly the domains NR1 and NR2 resulted highly conserved among the different genomes and ranged from 68 to 69 and 76 to 78 aa, respectively.

The α-type gliadins assigned to the *Gli-B2* locus, with the exception of Gli-B2–1, Gli-B2–8 and Gli-B2–9, are larger in size compared to those assigned to the other two loci. In particular, their size (including the signal peptide) ranged from 312 to 319 aa; differently, α-type gliadins assigned to the *Gli-A2* and *Gli-D2* loci ranged from 283 to 294 and from 282 to 309 aa, respectively (Aditional file 3: Table S1; Additional file 2: Figure S2). These differences were mainly due to the length of the two polyglutamine domains (QR1 and QR2) and the N-terminal domain (R1); in fact, QR1 was highly polymorphic among the different α-type gliadins and ranged from 18 to 29 aa for the *Gli-A2* locus, from 21 to 33 for the *Gli-B2* and from 12 to 25 for the *Gli-D2*. Similarly, the QR2 domain was different in length for the proteins associated to the *Gli-B2* (7–33) and *Gli-D2* (8–18) loci, while was identical in size (8 aa) for the α-type-gliadins associated to the *Gli-A2* locus (Additional file 3: Table S1).

InDels polymorphism was also observed in the R1 domain, except for the sequences associated to the *Gli-A2* locus, that were highly conserved (92 aa) (Additional file 3: Table S1). This domain was higher in size in the α-type gliadins associated to the *Gli-D2* locus compared to those of the other two loci. In particular, a peptide of seven amino acids (QPQLPYP), present from one to three times in sixteen genes out of the nineteen possible, is responsible for this difference (Additional file 2: Figure S2).

All the 18 sequences associated to the *Gli-A2* locus resulted more conserved among them than those of the other two loci; in particular the size of the 4 domains (R1, NR1, QR2 and NR2) was identical in the 18 α-type-gliadins; only the QR1 varied in size (from 18 to 29 aa). Differently for the proteins associated to the other two loci, the NR1 and NR2 domains were conserved, while R1 and QR2, as above reported, changed in length among the different sequences (Additional file 3: Table S1).

The position and number of cysteine residues (in total 6) were highly conserved in the deduced proteins except for the six sequences (Gli-B2–1, Gli-D2–4, Gli-D2–5, Gli-D2–6, Gli-D2–7 and Gli-D2–8) which showed the presence of an additional cysteine residue in the NR2 domain. The extra cysteine is the result of a point mutation in the codon TCC, coding for the amino acid serine, to TGC. The polymorphism was detected in the same position in all sequences above cited (Additional file 2: Figure S2).

### Phylogenetic analysis of α-type gliadins isolated from the bread wheat cv Pegaso

**Fig. 3:**
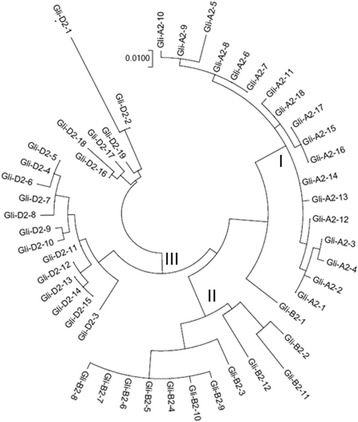
Phylogenetic relationships among the α-type gliadins isolated from the cultivar Pegaso. The tree was constructed based on neighbor-joining analysis using the entire coding regions

The phylogenetic relationships among the 49 α-type gliadins were investigated on the basis of the predicted amino acidic sequences. This analysis showed that it is possible to distinguish the α-type gliadins on the basis of their genomic origin (Fig. 3). In fact, the phylogenetic tree revealed that the deduced proteins were grouped in three large clusters (I, II and III): all the sequences associated at the *Gli-A2* locus were assigned to the cluster I; whereas, those associated at the *Gli-B2* and *Gli-D2* loci were assigned to the cluster II and III, respectively. Only the sequence Gli-B2–1 did not cluster with other sequences in the phylogenetic tree, but it was located at an intermediate position between the clusters I and II.

### Analysis of gene expression of the ω-, γ- and α-type gliadins

**Fig. 4:**
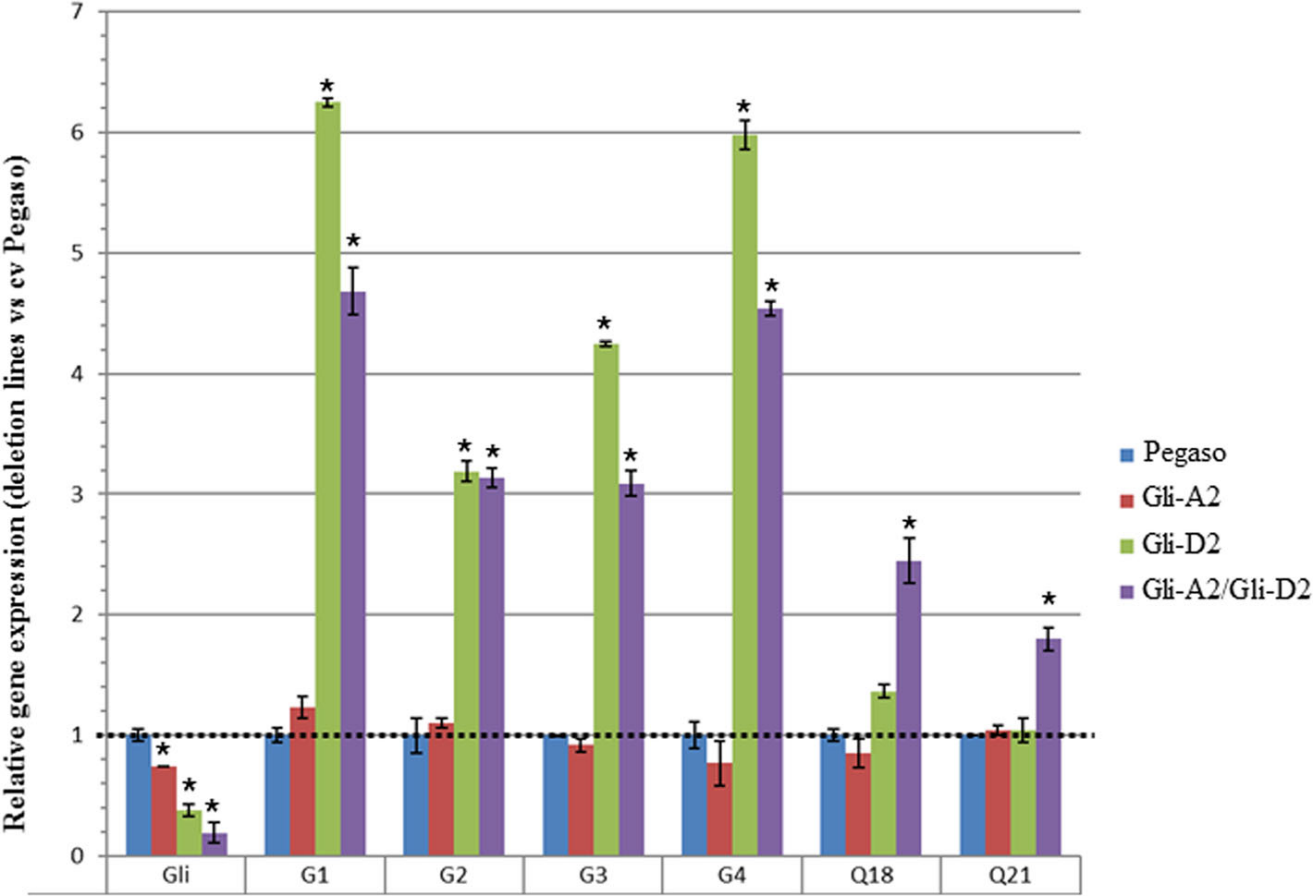
Analysis of relative gene expression of α- (Gli), γ- (G1, G2, G3 and G4) and ω-type (Q18 and Q21) gliadin transcripts in immature grain of bread wheat cv Pegaso and derived Gli-2 deletion lines. Dotted line indicates the relative transcription value of the control (cv. Pegaso). Each bar corresponds to the standard error, along with an asterisk to indicate where the deletion line values differed significantly (*P* < 0.05) from that of the wild type

To investigate at molecular level the reduction of α-type gliadin transcripts and to confirm the possible pleiotropic effects on the other classes of gliadins observed at protein level (Fig. 1), a Real Time PCR experiment was carried out on mRNA extracted from 24 DPA immature kernels of Pegaso, the two single deletion lines and the double one. Seven primer pairs were used: one specific for genes encoding α-type gliadins, four for γ-gliadins (G1, G2, G3, G4) [37] and two for ω-gliadins (Q18, Q21) [38]. Data shown in Fig. 4 highlighted that the single deletion lines (Gli-A2 and Gli-D2) had a significant reduction of the expression of α-type gliadins, that ranged from 25% in the Gli-A2 line to 62% in the Gli-D2 one. A more drastic reduction (80%) was observed in the double deletion line Gli-A2/Gli-D2. These results indicated that the genes located on the locus *Gli-D2* were more expressed or in a higher number of copies compared to the *Gli-A2* and *Gli-B2* loci.

The primer pairs, designed by Piston et al. [37], were used to quantify the levels of the four classes of γ-gliadin transcripts in immature grains. No changes were detected in the single deletion line Gli-A2; differently, these genes were up-regulated (up to 6 fold) in the other two deletion lines (Gli-D2 and Gli-A2/Gli-D2) (Fig. 4). A significant increase, but less evident, was also observed for the gene expression of the ω-gliadins in the genotype Gli-A2/Gli-D2, whereas no significant differences were observed in the single deletion lines (Fig. 4). All together, these data confirmed the existence of a compensatory mechanism as a consequence of the α-type gliadins down-regulation, in particular, when the locus *Gli-D2* was absent.

### Analysis of toxic and immunogenic peptides present in the 49 α-gliadins expressed in bread wheat cv Pegaso

The presence of toxic and immunogenic epitopes was investigated in the isolated α-gliadin genes. The analysis was focused on seven toxic epitopes previously described [25] and on five dominant immunogenic epitopes [36] (Additional file 4: Table S2 and Additional file 5: Table S3). Six toxic peptides, α-Glia (31–43), α-Glia (31–49), α-Glia (31–55), α-Glia (44–55), α-Glia (51–70) and α-Glia (56–75), were found in the N-terminal repetitive domain; whereas, the epitope α-Glia (206–217) was localized in the C-terminal domain. The position of the seven toxic epitopes was highly conserved, but a specificity in epitope occurrence on α-type gliadins assigned to the different genomes was observed. In particular the toxic peptides α-Glia (31–43) was specific of six and sixteen α-type gliadins present at the *Gli-B2* and *Gli-D2* loci, respectively. Similarly, the epitope α-Glia (31–49) was not detected in the proteins associated to the *Gli-A2* and was observed in fourteen α-type gliadins (five associated to the *Gli-B2* and nine to the G*li-D2*).

The peptides α-Glia (31–55) and α-Glia (206–217) were specific for the D and A genome, respectively. The first one was present in nine sequences; the last one was detected in all the α-type gliadins of the *Gli-A2* locus. Two epitopes (α-Glia (44–55) and α-Glia (51–70)) were detected in all the three *Gli*-2 loci, although they were more abundant in the α-type gliadins of the *Gli-A2* and *Gli-D2* loci compared to those of *Gli-B2*. α-Glia (56–75) was characteristic of five α-type gliadins of the *Gli-D2 * locus: it was not observed in any sequences assigned to the *Gli-A2* and *Gli-B2* loci (Additional file 4: Table S2).

The presence of the five immunogenic epitopes (Glia-α, Glia-α2, Glia-α9, Glia-α20 and 33-mer), able to specifically stimulate HLA-DQ2- or DQ8-restricted T cell clones, was investigated in deduced protein sequences of the 49 α-gliadins isolated from the cultivar Pegaso (Additional file 5: Table S3).

Glia-α9 and Glia-α20 were the most abundant, being identified in 30 and 25 sequences, respectively. Noteworthy, these epitopes were present only in one sequence produced from the *Gli-B2* locus. The Glia-α2 peptide was typical of α-gliadins associated at the D genome, being completely absent in those associated with the *Gli-A2* and *Gli-B2* loci. In particular, it was detected in 13 sequences out of 19 associated with the *Gli-D2* locus.

Gli-α was present in 24 sequences (13 associated with the D genome and 11 with the B genome): this epitope was not detected in α-gliadins deriving from the *Gli-A2* locus.

The 33-mer immunogenic peptide, that is considered the principal responsible of the development of the gluten dependent lesion since it is the most immunogenic powerful inducer [42], was completely absent in the α-gliadins assigned to *Gli-A2* and *Gli-B2 * loci; whereas it was detected in four proteins assigned to *Gli-D2* locus.

The comparison of deduced amino acid sequences between Pegaso and derived Gli-2 deletion lines highlighted that these latter had a reduced number of toxic and immunogenic epitopes (Table 3). This reduction was either quantitative or qualitative: the deletion lines had less α-type gliadin proteins (31 in the Gli-A2 genotype; 30 in Gli-D2 and 12 in Gli-A2/Gli-D2) compared to Pegaso (49); moreover, as some epitopes were specific of one of the three *Gli-2* loci, each deletion lines had lost some of these peptides. More in detail, the immunogenic peptides Glia-α2 and 33-mer and the toxic peptides α-Glia (31–55) and α-Glia (56–75) were completely absent in the Gli-D2 and Gli-A2/Gli-D2 deletion lines (Table 3). Similarly, the toxic peptide α-Glia (206–217) associated to the *Gli-A2* locus was not detected in the α-type gliadins present in the two genotypes lacking this locus (Gli-A2 and Gli-A2/Gli-D2 deletion lines) (Table 3). In conclusion, the total number of the immunogenic and toxic epitopes detected in the Gli-A2/Gli-D2 deletion line was, respectively, 13 and 17, drastically reduced if compared to the cultivar Pegaso, where 96 immunogenic and 111 toxic peptides were present.

**Table 3:** Number of deduced proteins and immunodominant/toxic epitopes detected in the bread wheat cv Pegaso and Gli-2 deletion lines

		Immunodominant epitopes					Toxic epitopes						
Genotypes	n° proteins	Glia-α	Glia-α2	Glia-α9	Glia-α20	33-mer	α-Glia (31–43)	α-Glia (31–49)	α-Glia (31–55)	α-Glia (44–55)	α-Glia (51–70)	α-Glia (56–75)	α-Glia (206–217)
Pegaso	49	24	13	30	25	4	22	14	9	27	16	5	18
Gli-A2	31	24	13	18	10	4	22	14	9	13	10	5	0
Gli-D2	30	11	0	13	16	0	6	5	0	15	7	0	18
Gli-A2/GliD2	12	11	0	1	1	0	6	5	0	5	1	0	0

## Discussion

### Copy number of expressed α-type gliadin genes and genetic localization

Genes coding α-gliadins present in the bread wheat cultivar Pegaso and the deletion lines were isolated and characterized. The analysis of α-gliadin transcripts leads to the isolation of 49 unique sequences. This number is congruent with that previously reported for other bread wheat cultivars [16, 43].

Although estimated copy numbers of α-type gliadins are consistently higher (from 100 up to 150) in genomic DNA [13], it was estimated that half of the α-type-gliadin genes are pseudogenes and thus not expressed [15]. In agreement with this estimation, Noma and colleagues [43] found that 40 out of 90 genes isolated from the bread wheat cultivar Chinese Spring (ca. 44%) had a premature stop codon.

Here, all the 49 α-type-gliadin genes were associated to one of the three *Gli-2* loci. The *Gli-B2* locus contains a lower number of genes (12) compared to the *Gli-A2* (18) and *Gli-D2* (19) loci. This result agrees with that observed by Salentijn et al. [44] that found a low amount of *Gli-B2* sequences in two landraces and in the bread wheat cultivar Lavett. However, the same authors analyzed several tetraploid and hexaploid wheat varieties and demonstrated that the copy number of expressed α-type gliadins associated to each *Gli-2* locus is extremely variable, either among the wheat species or within the same species among different varieties. For example, differently from the cv Pegaso, Noma and colleagues [43] found a similar number of intact genes coding α-type gliadins in the three *Gli-2* loci of the bread wheat cv Chinese Spring; in particular, the copy number was 16, 16 and 18 from the *Gli-A2*, *Gli-B2* and *Gli -D2* loci, respectively.

### Expression of gliadin fractions in bread wheat cv Pegaso and derived Gli-2 deletion lines

Our data highlighted a differential contribution of the three *Gli-2* loci in the production of α-type gliadin transcripts. In particular, the levels of transcript produced from the *Gli-D2* locus is larger than those deriving from the *Gli-A2* and *Gli-B2* loci. Kawaura and colleagues [45] carried out a study of expression patterns of α-type gliadin genes by using a bioinformatic approach and found that genes from the D genome were preferentially expressed compared to those deriving from the A or B genomes. Similarly, Salentijn et al. [44] investigated the expression of α-type-gliadins in some tetraploid and hexaploid wheats, demonstrating that large differences exist in the expression levels of the α-type gliadins associated at the three *Gli-2* loci. Noteworthy the Gli-A2/Gli-D2 deletion line contains a low level of α-type gliadins (20%), but a drastic increase of the other gliadin fractions was observed in A-PAGE. This observation was confirmed at molecular level by Real-Time PCR and lead us to hypothesize the existence of compensatory mechanisms. A similar mechanism of regulation was never observed among the gliadin fraction, but it has previously reported between glutenins and gliadins [46, 47].

### Number and position of the cysteine residues

The number and position of cysteine residues was conserved among the different α-type gliadins; In fact, six cysteine residues were present in most of the α-gliadins, except for six sequences which showed the presence of an additional cysteine residue in the NR2 domain (five associated to the *Gli-D2* locus and one to the *Gli-B2*). The presence of an extra cysteine residue in α-gliadins was also reported by different authors [35, 43]. Interestingly, a search on NCBI revealed that this extra cysteine was also present in the same position in different *T. aestivum* ssp. (*vulgare, compactum, spelta* and *macha*) and several accessions of *Ae tauschii*, thereby supporting the hypothesis that the point mutation responsible for the appearance of the odd cysteine residue occurred in the wild progenitor of the D genome to hexaploid wheats.

### Presence of toxic and immunogenic epitopes

It is scientifically proved that celiac disease is triggered from digestion-resistant gluten peptides, among these α-type gliadins are considered the major responsible [19, 26, 48–50]. In fact, as all the other gluten proteins, α-type gliadins, being rich in proline and glutamine, have few trypsin cleavage sites and are partially digested. Some of these peptides are recognized from T cell lines and can activate the adaptive and innate immune response [25]. Van den Broeck and colleagues [28] compared the genetic diversity of gluten proteins for the presence of CD epitopes in 36 modern European wheat varieties and in 50 landraces, finding that the presence of the Gli-α9 (one of the major immunodominant epitope present on α-type gliadins) was higher in modern wheats, as compared to old varieties. In the present work the number of α-type gliadins that have the epitope Gli-α9 is reduced in the lines lacking *Gli-A2* (18) and *Gli-D2* (14) loci compared to the cultivar Pegaso (31); this reduction is drastically evident in the Gli-A2/Gli-D2 deletion line, where the epitope Gli-α9 was detected only in one α-type gliadin (Table 3).

Some α-type gliadin isoforms contain the peptide 33-mer, that correspond to a cluster of immunogenic epitopes. This peptide is considered the main immunodominant toxic epitope and it is formed by six overlapping copies of three DQ2-restricted T-cell epitopes [16, 42].

Here, the 33-mer epitope was detected in four α-gliadin proteins, all encoded by the genes associated with the *Gli-D2* locus; for this reason, the Gli-D2 and Gli-A2/Gli-D2 deletion lines are completely devoid of this peptide.

The investigation of the presence of immunogenic and toxic epitopes among the 49 α-gliadin isoforms isolated from the bread wheat cv Pegaso highlighted that the proteins associated with the *Gli-D2* locus should be considered more highly reactive in respect to T cell toxicity. This result agrees with other works that found a higher amount of immunogenic and toxic epitopes in the α-gliadin proteins specific of the D genome compared to those derived from A and B genomes [17, 27, 51].

## Conclusions

Several wheat breeding programs have focused on gluten proteins, in particular on glutenins as these proteins are the main determinants of wheat technological properties. It has been hypothesized that breeding has contributed to the production of more “toxic” varieties, responsible for the increase in celiac disease and gluten sensitivity [28]. Since the gluten exposure is correlated with the incidence of CD, the use of wheat varieties with a reduced amount of toxic and immunogenic peptides seems to be a promising solution. At this regard, the single and double deletion lines here described could, very likely, be less harmful for people with a genetic predisposition for celiac disease, but that have still not developed the pathology, and would represents a good material for the development of safer wheat varieties. In particular, the Gli-A2/Gli-D2 deletion line contains a drastic reduction of immunogenic (−85%) and toxic (−85%) epitopes compared to the bread wheat cv Pegaso. The use of novel technologies (e. g. genome editing technologies), capable to introduce accurately specific changes into the wheat genome combined with the use of some deletion lines, lacking already several toxic epitopes, could be effective in the challenge of obtaining a complete safe wheat for people suffering of CD.

## Additional files

Additional file 1: Figure S1. Comparison of α-type gliadin genes. Alignment of the 49 full length nucleotidic sequences isolated from the bread wheat cv Pegaso were performed by the Clustal Omega multiple sequence alignment program. (DOC 112 kb)

Additional file 2: Figure S2. Amino acidic alignment of the signal peptide and the five domains corresponding to the 49 α-type gliadins isolated. Heptapeptide QPQLPYP in single copy or duplicated is highlighted in yellow; α-Glia (31–43) in gray; α-Glia (31–49) in gray + light blue; α-Glia (31–55) in gray + light blue + green; α-Glia (44–55) in light blue + green; α-Glia (51–70) underlined; α-Glia (56–75) in italic; α-Glia (206–217) in red; Glia-α in fuchsia; Glia-α2 in orange character; Glia-α9 in the black box; Glia-α20 in the blue box; the 33-mer peptide in the red box; the odd cysteine in bold and red character. (PDF 103 kb)

Additional file 3: Table S1. Accession number, molecular weight (MW) and length of the domains in the α-type gliadins isolated from bread wheat cv Pegaso. S=Signal peptide, R1 = Repetitive N-terminal domain; QR1 and QR2 = Polyglutamine domain 1 and 2; NR1 = Unique domain 1; NR2 = C-terminal unique domain. (DOCX 19 kb)

Additional file 4: Table S2. Presence or absence of seven toxic epitopes (**α-Glia (31–43):** PGQQQPFPPQQPY**; α-Glia (31–49):** PGQQQPFPPQQPYPQPQPF; **α-Glia (31–55):** PGQQQPFPPQQPYPQPQPFPSQQPY; **α-Glia (44–55):** PQPQPFPSQQPY**; α-Glia (51–70):** SQQPYLQLQPFPQPQLPY**; α-Glia (56–75):** LQLQPFPQPQLPYPQPQLPY; **α-Glia (206–217):** LGQGSFRPSQQN) in the 49 deduced amino acidic sequences isolated from the bread wheat cv Pegaso. (DOCX 17 kb)

Additional file 5: Table S3. Presence or absence of five immunogenic epitopes (**Glia-α:** QGSFQPSQQ; **Glia-α2:** PQPQLPYPQPQLPY; **Glia-α9:** PFPQPQLPY; **Glia-α20:** PFRPQQPYPQ; **33-mer:** LQLQPFPQPQLPYPQPQLPYPQPQLPYPQPQPF) in the 49 deduced amino acidic sequences isolated from the bread wheat cv Pegaso. (DOCX 15 kb)

## Abbreviations

A-PAGE: Acid-Polyacrylamide gel electrophoresis
CD: Celiac disease
DPA: Days Post Anthesis
EMA: Endomysial antibodies
EMBL-EBI: European Bioinformatics Institute
HMW-GS: High molecular weight glutenin subunits
IPTG: Isopropyl-β-D-1-tiogalattopyranoside
LB: Luria-Bertani
LMW-GS: Low molecular weight glutenin subunits
NCBI: National Center for Biotechnology Information
NR1: Unique domain 1
NR2: C-terminal unique domain
ORF: Open Reading Frame
P: Signal peptide
QR1: Polyglutamine domain 1
QR2: Polyglutamine domain 2
R1: N-terminal domain
tTG: Tissue transglutaminase
