# Supplementary material for: Production and molecular characterization of bread wheat lines with reduced amount of α-type gliadins
Source: BMC Plant Biol. 2017 Dec 19;17:248. doi: 10.1186/s12870-017-1211-3 (PMC5738072; doi:10.1186/s12870-017-1211-3)
Supplement: Supplementary file 1 — Comparison of α-type gliadin genes. Alignment of the 49 full length nucleotidic sequences isolated from the bread wheat cv Pegaso were performed by the Clustal Omega multiple sequence alignment program. (DOC 112 kb) [file 12870_2017_1211_MOESM1_ESM.doc]

Gli-B2-12 ATGAAGACCTTTCTCATCCTTGCCCTCCTTGCTATCGTGGCGACCACCGCCACAACTGCA 60

Gli-B2-2 ATGAAGACCTTTCTCATCCTTGCCCTCCTTGCTATCGTGGCGACCACCGCCACAACTGCA 60

Gli-B2-11 ATGAAGACCTTTCTCATCCTTGCCCTCCTTGCTATCGTGGCGACCACCGCCACAACTGCA 60

Gli-B2-1 ATGAAGACCTTTCTCATCCTTGCCCTCCTTGCTATCGTGGCGACCACCGCCACAACTGCA 60

Gli-A2-17 ATGAAGACCTTTCTCATCCTTGCCCTCCTTGCTATCGTGGCGACCACCGCCACAACTGCA 60

Gli-A2-15 ATGAAGACCTTTCTCATCCTTGCCCTCCTTGCTATCGTGGCGACCACCGCCACAACTGCA 60

Gli-A2-8 ATGAAGACCTTTCTCATCCTTGCCCTCCTTGCTATCGTGGCGACCACCGCCACAACTGCA 60

Gli-A2-4 ATGAAGACCTTTCTCATCCTTGTCCTCCTTGCTATTGTGGCGACCACCGCCACAACTGCA 60

Gli-A2-16 ATGAAGACCTTTCTCATCCTTGCCCTCCTTGCTATCGTGGCGACCACCGCCACAACTGCA 60

Gli-A2-11 ATGAAGACCTTTCTCATCCTTGCCCTCCTTGCTATCGTGGCGACCACTGCCACAACTGCA 60

Gli-A2-18 ATGAAGACCTTTCTCATCCTTGCCCTCCTTGCTATCGTGGCGACCACCGCCACAACTGCA 60

Gli-A2-6 ATGAAGACCTTTCTCATCCTTGCCCTCCTTGCTATCGTGGCGACCACCGCCACAACTGCA 60

Gli-A2-13 ATGAAGACCTTTCTCATCCTTGCCCTCCTTGCTATCGTGGCGACCACCGCCACAACTGCA 60

Gli-A2-14 ATGAAGACCTTTCTCATCCTTGCCCTCCTTGCTATCGTGGCGACCACCGCCACAACTGCA 60

Gli-A2-1 ATGAAGACCTTTCTCATCCTTGTCCTCCTTGCTATTGTGGCGACCACCGCCACAACTGCA 60

Gli-A2-2 ATGAAGACCTTTCTCATCCTTGTCCTCCTTGCTATTGTGGCGACCACCGCCACAACTGCA 60

Gli-A2-12 ATGAAGACCTTTCTCATCCTTGCCCTCCTTGCTATCGTGGCGACCACCGCCACAACTGCA 60

Gli-A2-3 ATGAAGACCTTTCTCATCCTTGTCCTCCTTGCTATTGTGGCGACCACCGCCACAACTGCA 60

Gli-A2-5 ATGAAGACCTTTCTCATCCTTGCCCTCCTTGCTATCGTGGCGACCACCGCCACAACTGCA 60

Gli-A2-7 ATGAAGACCTTTCTCATCCTTGCCCTCCTTGCTATCGTGGCGACCACCGCCACAACTGCA 60

Gli-A2-9 ATGAAGACCTTTCTCATCCTTGCCCTCCTTGCTATCGTGGCGACCACCGCCACAACTGCA 60

Gli-A2-10 ATGAAGACCTTTCTCATCCTTGCCCTCCTTGCTATCGTGGCGACCACCGCCACAACTGCA 60

Gli-D2-2 ATGAAGACCTTTCTCATCCTAGCCCTCCTTGCTATCGTGGCGACCACCGCCACAAGTGCA 60

Gli-D2-12 ATGAAGACCTTTCTCATCCTTGCCCTCCTTGCTATTGTAGCAACCACCGCCACAATTGCA 60

Gli-D2-13 ATGAAGACCTTTCTCATCCTTGCCCTCCTTGCTATTGTAGCAACCACCGCCACAATTGCA 60

Gli-D2-14 ATGAAGACCTTTCTCATCCTTGCCCTCCTTGCTATTGTAGCAACCACCGCCACAATTGCA 60

Gli-D2-15 ATGAAGACCTTTCTCATCCTTGCCCTCCTTGCTATTGTAGCAACCACCGCCACAATTGCA 60

Gli-D2-18 ATGAAGACCTTTCTCATCCTTGCCCTCCTTGCTATTGTGGCGACCACCGCCACAACTGCA 60

Gli-D2-16 ATGAAGACCTTTCTCATCCTTGCCCTCCTTGCTATTGTGGCGACCACCGCCACAACTGCA 60

Gli-D2-17 ATGAAGACCTTTCTCATCCTTGCCCTCCGTGCTATTGTAGCAACCACCGCCACAATTGCA 60

Gli-D2-19 ATGAAGACCTTTCTCATCCTAGCCCTCCTTGCTATCGTGGCGACCACCGCCACAAGTGCA 60

Gli-D2-3 ATGAAGACCTTTCTCATCCTTGCCCTCCTTGCTATTGTAGCAACCACCGCCACAATTGCA 60

Gli-D2-11 ATGAAGACCTTTCTCATCCTTGCACTCCTTGCTATTGTAGCAACCACCGCCACAATTGCA 60

Gli-D2-9 ATGAAGACCTTTCTCATCCTTGCCCTCCTTGCTATTGTAGCAACCACCGCCACAATTGCA 60

Gli-D2-10 ATGAAGACCTTTCTCATCCTTGCCCTCCTTGCTATTGTAGCAACCACCGCCACAATTGCA 60

Gli-D2-8 ATGAAGACCTTTCTCATCCTTGCCCTCCTTGCTATCGTGGCGACCACCGCCACAACTGCA 60

Gli-D2-7 ATGAAGACCTTTCTCATCCTTGCCCTCCTTGCTATTGTAGCAACCACCGCCACAATTGCA 60

Gli-D2-6 ATGAAGACCTTTCTCATCCTTTCCCTCCTTGCTATCGTGGCGACCACTGCCACAACTGCA 60

Gli-D2-4 ATGAAGACCTTTCTCATCCTTGCCCTCCTTGCTATTGTAGCAACCACCGCCACAATTGCA 60

Gli-D2-5 ATGAAGACCTTTCTCATCCTTGCCCTCCTTGCTATTGTAGCAACCACCGCCACAATTGCA 60

Gli-D2-1 ATGAAGACCTTTCTCATCCTTGCCCTCCTTGCTATCGTGGCGACCACCGCCACAAGTGCA 60

Gli-B2-3 ATGAAGACCTTTCTCATCCTTGCCCTCCTTGCTATTGTGGCGACCACCGCCACAACTGCA 60

Gli-B2-9 ATGAAGACCTTTCTCATCCTTTCCCTCCTTGCTATCGTGGCGACCACTGCCACAACTGCA 60

Gli-B2-7 ATGAAGACCTTTCTCATCCTTTCCCTCCTTGCTATCGTGGCGACCACTGCCACAACTGCA 60

Gli-B2-10 ATGAAGACCTTTCTCATCCTTTCCCTCCTTGCTATCGTGGCGACCACTGCCACAACTGCA 60

Gli-B2-4 ATGAAGACCTTTCTCATCCTTTCCCTCCTTGCTATCGTGGCGACCACTGCCACAACTGCA 60

Gli-B2-6 ATGAAGACCTTTCTCATCCTTTCCCTCCTTGCTATCGTGGCGACCACTGCCACAACTGCA 60

Gli-B2-5 ATGAAGACCTTTCTCATCCTTTCCCTCCTTGCTATCGTGGCGACCACTGCCACAACTGCA 60

Gli-B2-8 ATGAAGACCTTTCTCATCCTTTCCCTCCTTGCTATCGTGGCGACCACTGCCACAACTGCA 60

******************** **** ****** ** ** ***** ******* ****

Gli-B2-12 GTTAGAGTTCCAGTGCCACAACCGCAGCCACAAAATCCATCTCAGCCACAGCCACAAGGG 120

Gli-B2-2 GTTAGAGTTCCAGTGCCACAATTGCAGCCGCAAAATCCATCTCAGCAACAGCCACAAGAG 120

Gli-B2-11 GTTAGAGTTCCAGTGCCACAATTGCAGCCACAAAATCCATCTCAGCAACAGCCACAAGAG 120

Gli-B2-1 GTTAGAGTTCCAGTGCCACAATTGCAGCCACAAAATCCATCTCAGCAACAGCCACAAGAG 120

Gli-A2-17 GTTAGAGTTCCAGTGCCACAATTGCAGCCACAAAATCCATCTCAGCAACAGCCACAAGAG 120

Gli-A2-15 GTTAGAGTTCCAGTGCCACAATTGCAGCCACAAAATCCATCTCAGCAACAGCCACAAGAG 120

Gli-A2-8 GTTAGAGTTCCAGTGCCACAATTGCAGCCACAAAATCCATCTCAGCAACAGCCACAAGAG 120

Gli-A2-4 GTTAGATTTCCAGTGCCACAATTGCAGCCACAAAATCCATCTCAGCAACAGCCACAAGAG 120

Gli-A2-16 GTTAGAGTTCCAGTGCCACAATTGCAGCCACAAAATCCATCTCAGCAACAGCCACAAGAG 120

Gli-A2-11 GTTAGAGTTCCAGTGCCACAATTGCAGCCGCAAAATCCATCTCAGCAACAGCCACAAGAG 120

Gli-A2-18 GTTAGAGTTCCAGTGCCACAATTGCAGCCACAAAATCCATCTCAGCAACAGCCACAAGAG 120

Gli-A2-6 GTTAGAGTTCCAGTGCCACAATTGCAGCCACAAAATCCATCTCAGCAACAGCCACAAGAG 120

Gli-A2-13 GTTAGAGTTCCAGTGCCACAATTGCAGCCACAAAATCCATCTCAGCAACAGCCACAAGAG 120

Gli-A2-14 GTTAGAGTTCCAGTGCCACAATTGCAGCCACAAAATCCATCTCAGCAACAGCCACAAGAG 120

Gli-A2-1 GTTAGATTTCCAGTGCCACAATTGCAGCCACAAAATCCATCTCAGCAACAGCCACAAGAG 120

Gli-A2-2 GTTAGATTTCCAGTGCCACAATTGCAGCCACAAAATCCATCTCAGCAACAGCCACAAGAG 120

Gli-A2-12 GTTAGATTTCCAGTGCCACAATTGCAGCCACAAAATCCATCTCAGCAACAGCCACAAGAG 120

Gli-A2-3 GTTAGATTTCCAGTGCCACAATTGCAGCCACAAAATCCATCTCAGCAACAGCCACAAGAG 120

Gli-A2-5 GTTAGAGTTCCAGTGCCACAATTGCAGCCACAAAATCCATCTCAGCAACAGCCACAAGAG 120

Gli-A2-7 GTTAGAGTTCCAGTGCCACAATTGCAGCCACAAAATCCATCTCAGCAACAGCCACAAGAG 120

Gli-A2-9 GTTAGAGTTCCAGTGCCACAATTGCAGCCACAAAATCCATCTCAGCAACAGCCACAAGAG 120

Gli-A2-10 GTTAGAGTTCCAGTGCCACAATTGCAGCCACAAAATCCATCTCAGCAACAGCCACAAGAG 120

Gli-D2-2 GTTAGAGTTCCAGTGCCACAATTGCAGCCGCAAAATCCATCTCAACAACAACCACAAGAG 120

Gli-D2-12 GTTAGAGTTCCAGTGCCACAATTGCAGCCACAAAATCCATCTCAGCAACAACCACAAGAG 120

Gli-D2-13 GTTAGAGTTCCAGTGCCACAATTGCAGCCACAAAATCCATCTCAGCAACAACCACAAGAG 120

Gli-D2-14 GTTAGAGTTCCAGTGCCACAATTGCAGCCACAAAATCCATCTCAGCAACAACCACAAGAG 120

Gli-D2-15 GTTAGAGTTCCAGTGCCACAATTGCAGCCACAAAATCCATCTCAGCAACAACCACAAGAG 120

Gli-D2-18 GTTAGAGTTCCAGTGCCACAATTGCAGCTACAAAATCCATCTCAGCAACAGCCACAAGAG 120

Gli-D2-16 GTTAGAGTTCCAGTGCCACAATTGCAGCTACAAAATCCATCTCAGCAACAGCCACAAGAG 120

Gli-D2-17 GTTAGAGTTCCAGTGCCACAATTGCAGCCACAAAATCCATCTCAGCAACAGCCACAAGAG 120

Gli-D2-19 GTTAGAGTTCCAGTGCCACAATTGCAGCCGCAAAATCCATCTCAACAACAACCACAAGAG 120

Gli-D2-3 GTTAGAGTTCCAGTGCCACAATTGCAGCCACAAAATCCATCTCAGCAACAACCACAAGAG 120

Gli-D2-11 GTTAGAGTTCCAGTGCCACAATTGCAGCCACAAAATCCATCTCAGCAACAACCACAAGAG 120

Gli-D2-9 GTTAGAGTTCCAGTGCCACAATTGCAGCCACAAAATCCATCTCAGCAACAACCACAAAAG 120

Gli-D2-10 GTTAGAGTTCCAGTGCCACAATTGCAGCCACAAAATCCATCTCAGCAACAACCACAAAAG 120

Gli-D2-8 GTTAGAGTTCCAGTGCCACAATTGCAGCCACAAAATCCATCTCAGCAACAGCCACAAGAG 120

Gli-D2-7 GTTAGAGTTCCAGTGCCACAATTGCAGCCACAAAATCCATCTCAGCAACAACCACAAGAG 120

Gli-D2-6 GTTAGAGTTCCAGTGCCACAACTGCAGCCACAAAATCCATCTCAGCAACAACCACAAAAG 120

Gli-D2-4 GTTAGAGTTCCAGTGCCACAATTGCAGCCACAAAATCCATCTCAGCAACAACCACAAAAG 120

Gli-D2-5 GTTAGAGTTCCAGTGCCACAATTGCAGCCACAAAATCCATCTCAGCAACAACCACAAAAG 120

Gli-D2-1 GTTAGAGTTCCAGTGCCACAATTGCAGCCGCAAAATCCATCTCAACAACAACCACAAGAG 120

Gli-B2-3 GTTACAGTTCCAGTGCCACAATTGCAGCCACAAAATCCATCTCAGCAACAACCACAAAAG 120

Gli-B2-9 GTTAGAGTTCCAGTGCCACAATTGCAGCCGCAAAATCCATCTCAGCAACAACCACAAGAG 120

Gli-B2-7 GTTAGAGTTCCAGTGCCACAATTGCAGCCGCAAAATCCATCTCAGCAACAACCACAAGAG 120

Gli-B2-10 GTTAGAGTTCCAGTGCCACAATTGCAGCCGCAAAATCCATCTCAGCAACAACCACAAGAG 120

Gli-B2-4 GTTAGAGTTCCAGTGCCACAATTGCAGCCGCAAAATCCATCTCAGCAACAACCACAAGAG 120

Gli-B2-6 GTTAGAGTTCCAGTGCCACAATTGCAGCCGCAAAATCCATCTCAGCAACAACCACAAGAG 120

Gli-B2-5 GTTAGAGTTCCAGTGCCACAATTGCAGCCGCAAAATCCATCTCAGCAACAACCACAAGAG 120

Gli-B2-8 GTTAGAGTTCCAGTGCCACAATTGCAGCCGCAAAATCCATCTCAGCAACAACCACAAGAG 120

**** * ************** ***** ************** * *** ****** *

Gli-B2-12 CAAGTTCCATTGGTACAACAA---CAACAATTTCCAGGGCAGCAACAACAATTTCCACCA 177

Gli-B2-2 CAAGTTCCATTGGTACAACAA---CAACAATTTCCAGGGCAGCAACAACAATTTCCACCA 177

Gli-B2-11 CAAGTTCCATTGGTACAACAA---CAACAATTTCCAGGGCAGCAACAACAATTTCCACCA 177

Gli-B2-1 CAAGTTCCATTGGTACAACA---ACAACAATTTCTAGGGCAGCAACAACCATTTCCACCA 177

Gli-A2-17 CAAGTTCCATTGGTACAACA---ACAACAATTTCTAGGGCAGCAACAACCATTTCCACCA 177

Gli-A2-15 CAAGTTCCGTTGGTACAACA---ACAACAATTTCTAGGGCAGCAACAACCATTTCCACCA 177

Gli-A2-8 CAAGTTCCATTGGTACAACA---ACAACAATTTCTAGGGCAGCAACAACCATTTCCACCA 177

Gli-A2-4 CAAGTTCCATTGGTACAACA---ACAACAATTTCTAGGGCAGCAACAACCATTTCCACCA 177

Gli-A2-16 CAAGTTCCATTGGTACAACA---ACAACAATTTCTAGGGCAGCAACAACCATTTCCACCA 177

Gli-A2-11 CAAGTTCCATTGGTACAACA---ACAACAATTTCTAGGGCAGCAACAACCATTTCCACCA 177

Gli-A2-18 CAAGTTCCATTGGTACAACA---ACAACAATTTCTAGGGCAGCAACAACCATTTCCACCA 177

Gli-A2-6 CAAGTTCCGTTGGTACAACA---ACAACAATTTCTAGGGCAGCAACAACCATTTCCACCA 177

Gli-A2-13 CAAGTTCCGTTGGTACAACA---ACAACAATTTCTAGGGCAGCAACAACCATTTCCACCA 177

Gli-A2-14 CAAGTTCCATTGGTACAACA---ACAACAATTTCTAGGGCAGCAACAACCATTTCCACCA 177

Gli-A2-1 CAAGTTCCATTGGTACAACA---ACAACAATTTCTAGGGCAGCAACAACCATTTCCACCA 177

Gli-A2-2 CAAGTTCCATTGGTACAACA---ACAACAATTTCTAGGGCAGCAACAACCATTTCCACCA 177

Gli-A2-12 CAAGTTCCATTGGTACAACA---ACAACAATTTCTAGGGCAGCAACAACCATTTCCACCA 177

Gli-A2-3 CAAGTTCCATTGGTACAACA---ACAACAATTTCTAGGGCAGCAACAACCATTTCCACCA 177

Gli-A2-5 CAAGTTCCATTGGTACAACA---ACAACAATTTCTAGGGCAGCAACAACCATTTCCACCA 177

Gli-A2-7 CAAGTTCCATTGGTACAACA---ACAACAATTTCTAGGGCAGCAACAACCATTTCCACCA 177

Gli-A2-9 CAAGTTCCGTTGGTACAACA---ACAACAATTTCTAGGGCAGCAACAACCATTTCCACCA 177

Gli-A2-10 CAAGTTCCGTTGGTACAACA---ACAACAATTTCTAGGGCAGCAACAACCATTTCCACCA 177

Gli-D2-2 CAAGTTCCATTGATGCAACAACAACAACAATTTCCAGGGCAGCAAGAACAATTTCCACCA 180

Gli-D2-12 CAAGTTCCATTGGTACAACA---ACAACAATTTCCAGGGCAGCAACAACCATTTCCACCA 177

Gli-D2-13 CAAGTTCCATTGGTACAACA---ACAACAATTTCCAGGGCAGCAACAACCATTTCCACCA 177

Gli-D2-14 CAAGTTCCATTGGTACAACA---ACAACAATTTCCAGGGCAGCAACAACCATTTCCACCA 177

Gli-D2-15 CAAGTTCCATTGGTACAACA---ACAACAATTTCCAGGGCAGCAACAACCATTTCCACCA 177

Gli-D2-18 CAAGTTCCATTGGTACAAGA---ACAACAATTTCCAGGGCAGCAACAACCATTTCCACCA 177

Gli-D2-16 CAAGTTCCATTGGTACAAGA---ACAACAATTTCCAGGGCAGCAACAACCATTTCCACCA 177

Gli-D2-17 CAAGTTCCATTGGTACAAGA---ACAACAATTTCCAGGGCAGCAACAACCATTTCCACCA 177

Gli-D2-19 CAAGTTCCATTGGTACAACA---ACAACAATTTCCAGGGCAGCAACAACCATTTCCACCA 177

Gli-D2-3 CAAGTTCCATTGGTACAACA---ACAGCAATTTCCAGGGCAGCAACAACCATTTCCACCA 177

Gli-D2-11 CAAGTTCCATTGGTACAACA---ACAACAATTTCCAGGGCAGCAACAACCATTTCCACCA 177

Gli-D2-9 CAAGTTCCATTGGTACAACA---ACAACAATTTCCAGGGCAGCAACAACCATTTCCACCA 177

Gli-D2-10 CAAGTTCCATTGGTACAACA---ACAACAATTTCCAGGGCAGCAACAACCATTTCCACCA 177

Gli-D2-8 CAAGTTCCATTGGTACAACA---ACAACAATTTCCAGGGCAGCAACAACAATTTCCACCA 177

Gli-D2-7 CAAGTTCCATTGGTACAACA---ACAACAATTTCCAGGGCAGCAACAACCATTTCCACCA 177

Gli-D2-6 CAAGTTCCATTGGTACAACA---ACAACAATTTCCAGGGCAGCAACAACCATTTCCACCA 177

Gli-D2-4 CAAGTTCCATTGGTACAACA---ACAACAATTTCCAGGGCAGCAACAACCATTTCCACCA 177

Gli-D2-5 CAAGTTCCATTGGTACAACA---ACAACAATTTCCAGGGCAGCAACAACCATTTCCACCA 177

Gli-D2-1 CAAGTTCCATTGATGCAACAACAACAACAATTTCCAGGGCAGCAAGAACAATTTCCACCA 180

Gli-B2-3 CAAGTTCCATTGGTGCAACAA---CAACAATTTCTAGGGCAGCAACAACCATTTCCACCA 177

Gli-B2-9 CAAGTTTCGTTGGTGCAACAA---CTACAATATCCAGGGCAACAACAACCATTTCCACCA 177

Gli-B2-7 CAAGTTTCGTTGGTGCAACAA---CTACAATATCCAGGGCAACAACAACCATTTCCACCA 177

Gli-B2-10 CAAGTTTCGTTGGTGCAACAA---CTACAATATCCAGGGCAACAACAACCATCTCCACCA 177

Gli-B2-4 CAAGTTTCGTTGGTGCAACAA---CTACAATATCCAGGGCAACAACAACCATTTCCACCA 177

Gli-B2-6 CAAGTTTCGTTGGTGCAACAA---CTACAATATCCAGGGCAACAACAACCATTTCCACCA 177

Gli-B2-5 CAGGTTTCGTTGGTGCAACAA---CTACAATATCCAGGGCAACAACAACCATTTCCACCA 177

Gli-B2-8 CAAGTTTCGTTGGTGCAACAA---CTACAATATCCAGGGCAACAACAACCATTTCCACCA 177

** *** * *** * *** * * **** ** ****** *** *** ** *******

Gli-B2-12 CAACAGCCATATCCGCAGCCGCAACCATTTCCATCACAACAACCATATCTGCAATTGCAA 237

Gli-B2-2 CAACAGCCATATCCGCAGCCGCAACCATTTCCATCACAACAACCATATCTGCAGCTGCAA 237

Gli-B2-11 CAACAGCCATATCCGCAGCCGCAACCATTTCCATCACAACAACCATATCTGCAGCTGCAA 237

Gli-B2-1 CAACAACCATATCCACAGCCGCAACCATTTCCATCACAACAACCATATCTGCAACTGCAA 237

Gli-A2-17 CAACAACCATATCCACAGCCGCAACCATTTCCATCACAACAACCATATCTGCAACTGCAA 237

Gli-A2-15 CAACAACCATATCCACAGCCGCAACCATTTCCATCACAACAACCATATCTGCAACTGCAA 237

Gli-A2-8 CAACAACCATATCCACAGCCGCAACCATTTCCATCACAACAACCATATCTGCAACTGCAA 237

Gli-A2-4 CAACAACCATATCCACAGCCGCAACCATTTCCATCACAACAACCATATCTGCAACTGCAA 237

Gli-A2-16 CAACAACCATATCCACAGCCGCAACCATTTCCATCACAACAACCATATCTGCAACTGCAA 237

Gli-A2-11 CAACAACCATATCCACAGCCGCAACCATTTCCATCACAACTACCATATCTGCAGCTGCAA 237

Gli-A2-18 CAACAACCATATCCACAGCCGCAACCATTTCCATCACAACTACCATATCTGCAGCTGCAA 237

Gli-A2-6 CAACAACCATATCCACAGCCGCAACCATTTCCATCACAACAACCATATCTGCAACTGCAA 237

Gli-A2-13 CAACAACCATATCCACAGCCGCAACCATTTCCATCACAACAACCATATCTGCAACTGCAA 237

Gli-A2-14 CAACAACCATATCCACAGCCGCAACCATTTCCATCACAACAACCATATCTGCAACTGCAA 237

Gli-A2-1 CAACAACCATATCCACAGCCGCAACCATTTCCATCACAACTACCATATCTGCAGCTGCAA 237

Gli-A2-2 CAACAACCATATCCACAGCCGCAACCATTTCCATCACAACTACCATATCTGCAGCTGCAA 237

Gli-A2-12 CAACAACCATATCCACAGCCGCAACCATTTCCATCACAACTACCATATCTGCAGCTGCAA 237

Gli-A2-3 CAACAGCCATATCCACAGCCGCAACCATTTCCATCACAACTACCATATCTGCAGCTGCAA 237

Gli-A2-5 CAACAACCATATCCACAGCCGCAACCATTTCCATCACAACAACCATATCTGCAACTGCAA 237

Gli-A2-7 CAACAACCATATCCACAGCCGCAACCATTTCCATCACAACAACCATATCTGCAACTGCAA 237

Gli-A2-9 CAACAACCATATCCACAGCCGCAACCATTTCCATCACAACAACCATATCTGCAACTGCAA 237

Gli-A2-10 CAACAACCATATCCACAGCCGCAACCATTTCCATCACAACAACCATATCTGCAACTGCAA 237

Gli-D2-2 CAACAGCCATATCCGCATCAGCAACCATTTCCATCACAACAACCATATCCGCAGCCGCAA 240

Gli-D2-12 CAACAGCCATATCCGCAGCCGCAACCATTTCCATCACAACAACCATATCTGCAGCTGCAA 237

Gli-D2-13 CAACAGCCATATCCGCAGCCGCAACCATTTCCATCACAACAACCATATCTGCAGCTGCAA 237

Gli-D2-14 CAACAGCCATATCCGCAGCCGCAACCATTTCCATCACAACAACCATATCTGCAGCTGCAA 237

Gli-D2-15 CAACAGCCATATCCGCAGCCGCAACCATTTCCATCACAACAACCATATCTGCAGCTGCAA 237

Gli-D2-18 CAACAGCCATATCCGCAGCCGCAACCATTTCCATCACAACAACCATATCTGCAGCTGCAA 237

Gli-D2-16 CAACAGCCATATCCGCAGCCGCAACCATTTCCATCACAACAACCATATCTGCAGCTGCAA 237

Gli-D2-17 CAACAGCCATATCCGCAGCCGCAACCATTTCCATCACAACAACCATATCTGCAGCTGCAA 237

Gli-D2-19 CAACAGCCATATCCGCAGCTGCAACCATTTCCATCACAACAACCATATATGCAGCTGCAA 237

Gli-D2-3 CAACAACCATATCCGCAGCCGCAACCATTTCCATCACAACAACCATATCTGCAGCTGCAA 237

Gli-D2-11 CAACAGCCATATCCGCAGCCGCAACCATTTCCATCACAACAACCATATCTGCAGCTGCAG 237

Gli-D2-9 CAACAGCCATATCCGCAGCTGCAACCATTTCCATCACAACAACCATATATGCAGCTGCAA 237

Gli-D2-10 CAACAGCCATATCCGCAGCTGCAACCATTTCCATCACAACAACCATATATGCAGCTGCAA 237

Gli-D2-8 CAACAGCCATATCCGCAGCCGCAACCATTTCCATCACAACAACCATATATGCAGCTGCAA 237

Gli-D2-7 CAACAGCCATATCCGCAGCTGCAACCATTTCCATCACAACAACCATATATGCAGCTGCAA 237

Gli-D2-6 CAACAGCCATATCCGCAGCTGCAACCATTTCCATCACAACAACCATATATGCAGCTGCAA 237

Gli-D2-4 CAACAGCCATATCCGCAGCTGCAACCATTTCCATCACAACAACCATATATGCAGCTGCAA 237

Gli-D2-5 CAACAGCCATATCCGCAGCTGCAACCATTTCCATCACAACAACCATATATGCAGCTGCAA 237

Gli-D2-1 CAACAGCCATATCCGCATCAGCAACCATTTCCATCACAACAACCATATCCGCAGCCGCAA 240

Gli-B2-3 CAACAGCCATATCCGCAGCCGCAACCATTTCCATCACAACAACCATATCCACAGCCACAA 237

Gli-B2-9 CAACAACCATATCCACAACCGCA------------------------------------- 200

Gli-B2-7 CAACAGCCATATCCGCAGCCGCAACCATTTCCATCACAACAACCATTGCCGCAACCGCAA 237

Gli-B2-10 CAACAGCCATATCCGCAGCCGCAACCATTTCCATCACAACAACCATTGCCGCAACCGCAA 237

Gli-B2-4 CAACAGCCATATCCGCAGCCGCAACCATTTCCATCACAACAACCATTGCCGCAACCGCAA 237

Gli-B2-6 CAACAGCCATATCCGCAGCCGCAACCATTTCCATCACAACAACCATTGCCGCAACCGCAA 237

Gli-B2-5 CAACAGCCATATCCGCAGCCGCAACCATTTCCATCACAACAACCATTGCCGCAACCGCAA 237

Gli-B2-8 CAACAGCCATATCCGCAGCCGCAACCATTTCCATCACAACAACCATTGCCGCAACCGCAA 237

***** ******** ** * ***

Gli-B2-12 CCATTTCCACAACCGCAACCATTTC---------------------------CGCCACAA 270

Gli-B2-2 CCATTTCCGCAGCCGCAACCATTTC---------------------------CGCCACAA 270

Gli-B2-11 CCATTTCCGCAGCCGCAACCATTTC---------------------------CGCCACAA 270

Gli-B2-1 CCATTTCCGCAGCCGCAACTACCATATTCGCAAC-------------------------- 271

Gli-A2-17 CCATTTCCGCAGCCGCAACTACCATATTCGCAAC-------------------------- 271

Gli-A2-15 CCATTTCCGCAGCCGCAACTATCATATTCGCAGC-------------------------- 271

Gli-A2-8 CCATTTCCGCAGCCGCAACTACCATATTCGCAAC-------------------------- 271

Gli-A2-4 CCATTTCCGCAGCCGCAACTACCATATTCGCAAC-------------------------- 271

Gli-A2-16 CCATTTCCGCAGCCGCAACTACCATATTCGCAAC-------------------------- 271

Gli-A2-11 CCATTTCCGCAGCCGCAACTACCATATTCACAGC-------------------------- 271

Gli-A2-18 CCATTTCCGCAGCCGCAACTACCATATTCACAGC-------------------------- 271

Gli-A2-6 CCATTTCCGCAGCCGCAACTATCATATTCGCAGC-------------------------- 271

Gli-A2-13 CCATTTCCGCAGCCGCAACTATCATATTCGCAGC-------------------------- 271

Gli-A2-14 CCATTTCCGCAGCCGCAACTACCATATTCGCAAC-------------------------- 271

Gli-A2-1 CCATTTCCGCAGCCGCAACTACCATATTCACAGC-------------------------- 271

Gli-A2-2 CCATTTCCGCAGCCGCAACTACCATATTCACAGC-------------------------- 271

Gli-A2-12 CCATTTCCGCAGCCGCAACTACCATATTCACAGC-------------------------- 271

Gli-A2-3 CCATTTCCGCAGCCGCAACTACCATATTCACAGC-------------------------- 271

Gli-A2-5 CCATTTCCGCAGCCGCAACTATCATATTCGCAGC-------------------------- 271

Gli-A2-7 CCATTTCCGCAGCCGCAACTACCATATTCGCAAC-------------------------- 271

Gli-A2-9 CCATTTCCGCAGCCGCAACTATCATATTCGCAGC-------------------------- 271

Gli-A2-10 CCATTTCCGCAGCCGCAACTATCGTATTCGCAGC-------------------------- 271

Gli-D2-2 CCATTTCCGCCA---------------------------------------------CAA 255

Gli-D2-12 CCATTTCCGCAGCCGCAACTACCATATCCGCAGCCGCAACTACCATATCCGCAGCCGCAA 297

Gli-D2-13 CCATTTCCGCAGCCGCAACTACCATATCCGCAGCCGCAACTACCATATCCGCAGCCGCAA 297

Gli-D2-14 CCATTTCCGCAGCCGCAACTACCATATCCGCAGCCGCAACTACCATATCCGCAGCCGCAA 297

Gli-D2-15 CCATTTCCGCAGCCGCAACTACCATATCCGCAGCCGCAACTACCATATCCGCAGCCGCAA 297

Gli-D2-18 CCATTTCCACAGCCGCAACTACCA------------------------------------ 261

Gli-D2-16 CCATTTCCACAGCCGCAACTACCA------------------------------------ 261

Gli-D2-17 CCATTTCCACAGCCGCAACTACCA------------------------------------ 261

Gli-D2-19 CCATTTCCGCAGCCGCAACTACCATATCCGCAGCCG---------------------CAA 276

Gli-D2-3 CCATTTCCGCAGCCGCAACTACCATATCCGCAGCCG---------------------CAA 276

Gli-D2-11 CCATTTCCGCAGCCGCAACTACCATATCCGCAGCCG---------------------CAT 276

Gli-D2-9 CCATTTCCGCAGCCGCAACTACCATATCCGCAGCCG---------------------CAA 276

Gli-D2-10 CCATTTCCGCAGCCGCAACTACCATATCCGCAGCCG---------------------CAA 276

Gli-D2-8 CCATTTCCGCAGCCGCAACTACCATATCCGCAGCCG---------------------CAA 276

Gli-D2-7 CCATTTCCGCAGCCGCAACTACCATATCCGCAGCCG---------------------CAA 276

Gli-D2-6 CCATTTCCGCAGCCGCAACTACCATATCCGCAGCCG---------------------CAA 276

Gli-D2-4 CCATTTCCGCAGCCGCAACTACCATATCCGCAGCCG---------------------CAA 276

Gli-D2-5 CCATTTCCGCAGCCGCAACTACCATATCCGCAGCCG---------------------CAA 276

Gli-D2-1 CCATTTCCGCCACA---------------------------------------------A 255

Gli-B2-3 CCATTTCTGCCACA---------------------------------------------A 252

Gli-B2-9 ------------------------------------------------------------ 200

Gli-B2-7 CCATTTCTGCCACA---------------------------------------------A 252

Gli-B2-10 CCATTTCTGCCACA---------------------------------------------A 252

Gli-B2-4 CCATTTCTGCCACA---------------------------------------------A 252

Gli-B2-6 CCATTTCTGCCACA---------------------------------------------A 252

Gli-B2-5 CCATTTCTGCCACA---------------------------------------------A 252

Gli-B2-8 CCATTTCTGCCACA---------------------------------------------A 252

Gli-B2-12 CTACCATATCCGCAGCCACCACCATTTTCACCACAACAACAATATCCACAACCGCAACCA 330

Gli-B2-2 CTACCATATCCGCAGCCGCAATCATTTCCACCACAACAACCATATCCACAACAGCAACCA 330

Gli-B2-11 CTACCATATCCGCAGCCGCAATCATTTCCACCACAACAACCATATCCACAACAGCAACCA 330

Gli-B2-1 ----------------CACAACCATTTCGACCACAACAACCATATCCACAACCGCAACCA 315

Gli-A2-17 ----------------CACAACCATTTCGACCACAACAACCATATCCACAACCGCAACCA 315

Gli-A2-15 ----------------CACAACCATTTCGACCACAACAACCATATCCACAACCGCAACCA 315

Gli-A2-8 ----------------CACAACCATTTCGACCACAACAACCATATCCACAACCGCAACCA 315

Gli-A2-4 ----------------CACAACCATTTCGACCACAACAACCATATCCACAACCGCAACCA 315

Gli-A2-16 ----------------CACAACCATTCCGACCACAACAACCATATCCACAACCGCAACCA 315

Gli-A2-11 ----------------CACAACCATTTCGACCACAACAACCATATCCACAACCGCAACCA 315

Gli-A2-18 ----------------CACAACCATTTCGACCACAACAACCATATCCACAACCGCAACCA 315

Gli-A2-6 ----------------CACAACCATTTCGACCACAACAACCATATCCACAACCGCAACCA 315

Gli-A2-13 ----------------CACAACCATTTCGACCACAACAACCATATCCACAACCGCAACCA 315

Gli-A2-14 ----------------CACAACCATTTCGACCACAACAACCATATCCACAACCGCAACCA 315

Gli-A2-1 ----------------CACAACCATTTCGACCACAACAACCATATCCACAACCGCAACCA 315

Gli-A2-2 ----------------CACAACCATTTCGACCACAACAACCATATCCACAACCGCAACCA 315

Gli-A2-12 ----------------CACAACCATTTCGACCACAACAACCATATCCACAACCGCAACCA 315

Gli-A2-3 ----------------CACAACCATTTCGACCACAACAACCATATCCACAACCGCAACCA 315

Gli-A2-5 ----------------CACAACCATTTCGACCACAACAACTATATCCACAACCGCAACCA 315

Gli-A2-7 ----------------CACAACCATTTCGACCACAACAACCATATCCACAACCGCAACCA 315

Gli-A2-9 ----------------CACAACCATTTCGACCACAACAACTATATCCACAACCGCAACCA 315

Gli-A2-10 ----------------CACAACCATTTCGACCACAACAACTATATCCACAACCGCAACCA 315

Gli-D2-2 CTACCATATCCGCAGACGCAACCATTTCCACCACAACAACCATATCCACAACCGCAACCA 315

Gli-D2-12 CTACCATATCCGCAGCCGCAACCATTTCGACCACAACAACCATATCCACAATCGCAACCA 357

Gli-D2-13 CTACCATATCCGCAGCCGCAACCATTTCGACCACAACAACCATATCCACAATCGCAACCA 357

Gli-D2-14 CTACCATATCCGCAGCCGCAACCATTTCGACCACAACAACCATATCCACAATCGCAACCA 357

Gli-D2-15 CTACCATATCCGCAGCCGCAACCATTTCGACCACAACAACCATATCCACAATCGCAACCA 357

Gli-D2-18 ------TATCCGCAGCCGCAACCATTTCGACCACAACAACCATATCCACAGCCGCAACCA 315

Gli-D2-16 ------TATCCGCAGCCGCAACCATTTCGACCACAACAACCATATCCACAGCCGCAACCA 315

Gli-D2-17 ------TATCCGCAGCCGCAACCATTTCGACCACAACAACCATATCCACAGCCGCAACCA 315

Gli-D2-19 CTACCATATCCGCAGCCGCAACCATTTCGACCACAACAATCATATCCACAACCGCAACCA 336

Gli-D2-3 CTACCATATCCGCAGCCGCAACCATTTCGACCACAACAACCATATCCACAACCGCAACCA 336

Gli-D2-11 CTACCATATCCGCAGCCGCAACCATTTCGACCACAACAACCATATCCACAACCGCAACCA 336

Gli-D2-9 CTACCATATCCGCAGCCGCAACCATTTCGACCACAACAATCATATCCACAACCGCAACCA 336

Gli-D2-10 CTACCATATCCGCAGCCGCAACCATTTCGACCACAACAATCATATCCACAACCGCAACCA 336

Gli-D2-8 CTACCATATCCGCAGCCGCAACCATTTCGACCACAACAATCATATCCACAACCGCAACCA 336

Gli-D2-7 CTACCATATCCGCAGCCGCAACCATTTCGACCACAACAATCATATCCACAACCGCAACCA 336

Gli-D2-6 CTACCATATCCGCAGCCGCAACCATTTCGACCACAACAATCATATCCACAACCGCAACCA 336

Gli-D2-4 CTACCATATCCGCAGCCGCAACCATTTCGACCACAACAATCATATCCACAACCGCAACCA 336

Gli-D2-5 CTACCATATCCGCAGCCGCAGCCATTTCGACCACAACAATCATATCCACAACCGCAACCA 336

Gli-D2-1 CTACCATATCCGCAGACGCAACCATTTCCACCACAACAACCATATCCACAACCGCAACCA 315

Gli-B2-3 CTACCATATCCGCGTCCGCAACCATTTCTACCACAACAACCATATCCACAACCGCAACCA 312

Gli-B2-9 --------------------------------------------------------ACCA 204

Gli-B2-7 CTACCATATCCGCAGCCACAACCATTTCCACCACAACAACCATATCCACAACCGCAACCA 312

Gli-B2-10 CTACCATATCCGCAGCCACAACCATTTCCACCACAACAACCATATCCACAACCGCAACCA 312

Gli-B2-4 CTACCATATCCGCAGCCACAACCATTTCCACCACAACAACCATATCCACAACCGCAACCA 312

Gli-B2-6 CTACCATATCCGCAGCCACAACCATTTCCACCACAACAACCATATCCACAACCGCAACCA 312

Gli-B2-5 CTACCATATCCGCAGCCACAACCATTTCCACCACAACAACCATATCCACAACCGCAACCA 312

Gli-B2-8 CTACCATATCCGCAGCCACAACCATTTCCACCACAACAACCATATCCACAACCGCAACCA 312

****

Gli-B2-12 CAGTATCCGCAACCACAACAACCAATTTCACAGCAACAAGCACAACAACAACAACAACAA 390

Gli-B2-2 CAGTATCTACAACCACAACAACCAATTTCGCAGCAACAAGCACAACAACAACAACAACAA 390

Gli-B2-11 CAGTATCTACAACCACAACAACCAATTTCGCAGCAACAAGCACAACAACAACAACAACAA 390

Gli-B2-1 CAGTATTCGCAACCACAACAACCAATTTCACAGCAGCAGCAGCAGCAACAACAACAACAA 375

Gli-A2-17 CAGTATTCGCAACCACAACAACCAATTTCACAGCAGCAGCAGCAGCAGCAACAACA---A 372

Gli-A2-15 CAGTATTCGCAACCACAACAACCAATTTCACAGCAGCAGCAGCAGCAGCAACAACAACAA 375

Gli-A2-8 CAGTATTCGCAACCACAACAACCAATTTCACAGCAGCAGCAGCAGCAGCAACAACAGCAA 375

Gli-A2-4 CAGTATTCGCAACCACAACAACCAATTTCACAGCAGCAGCAGCAGCAGCAACAACAACAA 375

Gli-A2-16 CAGTATTCGCAACCACAACAACCAATTTCACAGCAGCAGCAGCAGCAGCAACAACAACAA 375

Gli-A2-11 CAGTATTCGCAACCACAACAACCAATTTCACAGCAGCAGCAGCAGCAGCAACAACAACA- 374

Gli-A2-18 CAGTATTCGCAACCACAACAACCAATTTCACAGCAGCAGCAGCAGCAGCAACAACAACA- 374

Gli-A2-6 CAGTATTCGCAACCACAACAACCAATTTCACAGCAGCAGCAGCAGCAGCAACAACAACAA 375

Gli-A2-13 CAGTATTCGCAACCACAACAACCAATTTCACAGCAGCAGCAGCAGCAGCAACAACAACA- 374

Gli-A2-14 CAGTATTCGCAACCACAACAACCAATTTCACAGCAGCAGCAGCAGCAGCAACAACAACA- 374

Gli-A2-1 CAGTATTCGCAACCACAACAACCAATTTCACAGCAGCAGCAGCAGCAGCAACAACAACA- 374

Gli-A2-2 CAGTATTCGCAACCACAACAACCAATTTCACAGCAGCAGCAGCAGCAGCAACAACAACA- 374

Gli-A2-12 CAGTATTCGCAACCACAACAACCAATTTCACAGCAGCAGCAGCAGCAGCAACAACAACA- 374

Gli-A2-3 CAGTATTCGCAACCACAACAACCAATTTCACAGCAGCAGCAGCAACAACAACAACAACAA 375

Gli-A2-5 CAGTATTCGCAACCACAACAACCAATTTCACAGCAGCAGCAGCAACAACAACAACAACAA 375

Gli-A2-7 CAGTATTCGCAACCACAACAACCAATTTCACAGCAGCAGCAGCAGCAGCAACAACAACAA 375

Gli-A2-9 CAGTATTCGCAACCACAACAACCAATTTCACAGCAGCAGCAGCAACAACAACAACAACAA 375

Gli-A2-10 CAGTATTCGCAACCACAACAACCAATTTCACAGCAGCAGCAGCAACAACAACAACAACAA 375

Gli-D2-2 CAGTATCCGCAACCACAACAACCAATTTCGCAGCAACAAGCA------------------ 357

Gli-D2-12 CAGTATTCGCAACCACAACAACCAATTTCGCAGCAGCAGCAGCAGCAGCAACAACAACA- 416

Gli-D2-13 CAGTATTCGCAACCACAACAACCAATTTCGCAGCAGCAGCAGCAACAACAACAACAACA- 416

Gli-D2-14 CAGTATTCGCAACCACAACAACCAATTTCGCAGCAGCAGCAGCAACAACAACAACAACA- 416

Gli-D2-15 CAGTATTCGCAACCACAACAACCAATTTCGCAGCAGCAGCAGCAACAACAACAACAACA- 416

Gli-D2-18 CAGTATTCGCAACCACAACAACCAATTTCGCAGCAGCAGCAGCAGCAGCAGCAACA---- 371

Gli-D2-16 CAGTATTCGCAACCACAACAACCAATTTCGCAGCAGCAGCAGCAGCAGCAGCAACA---- 371

Gli-D2-17 CAGTATTCGCAACCACAACAACCAATTTCGCAGCAGCAGCAGCAGCAGCAGCAACA---- 371

Gli-D2-19 CAGTATTCGCAACCACAACAACCAATTTCGCAGCAGCAGCAGCAGCAGCAGCAA------ 390

Gli-D2-3 CAGTATTCGCAACCACAACAACCAATTTCGCAGCAGCAA--------------------- 375

Gli-D2-11 CAGTATTCGCAACCACAACAACCAATTTCGCAGCAGCAGCA------------A------ 378

Gli-D2-9 CAGTATTCGCAACCACAACAACCAATTTCGCAGCAGCAGCAGCAGCAGCAGCAA------ 390

Gli-D2-10 CAGTATTCGCAACCACAACAACCAATTTCGCAGCAGCAGCAGCAGCAGCAGCAA------ 390

Gli-D2-8 CAGTATTCGCAACCACAACAACCAATTTCGCAGCAGCAGCAGCAGCAGCAGCAA------ 390

Gli-D2-7 CAGTATTCGCAACCACAACAACCAATTTCGCAGCAGCAGCAGCAGCAGCAG--------- 387

Gli-D2-6 CAGTATTCGCAACCACAACAACCAATTTCGCAGCAGCAGCAGCAGCAGCAGCAA------ 390

Gli-D2-4 CAGTATTCGCAACCACAACAACCAATTTCGCAGCAGCAGCAGCAGCAGCAGCAA------ 390

Gli-D2-5 CAGTATTCGCAACCACAACAACCAATTTCGCAGCAGCAGCAGCAGCA---GCAA------ 387

Gli-D2-1 CAGTATCCGCAACCACAACAACCAATTTCGCAGCAACAAGCACAACAACAACAACAA--- 372

Gli-B2-3 CAGTATCCGCAACCACAACAACCAATTTCACAGCAACAAGCACAACAAGCACAACAACAA 372

Gli-B2-9 CAGTATCCGCAACCACAACAACCAATTTCACAGCAACAAGCACAACAAGCACAACAACAA 264

Gli-B2-7 CAGTATCCGCAACCACAACAACCAATTTCACAGCAACAAGCACAACAAGCACAACAACAA 372

Gli-B2-10 CAGTATCCACAACCACAACAACCAATTTCACAGCAACAAGCACAACAAGCACAACAACAA 372

Gli-B2-4 CAGTATCCGCAACCACAACAACCAATTTCACAGCAACAAGCACAACAAGCACAACAACAA 372

Gli-B2-6 CAGTATCCGCAACCACAACAACCAATTTCACAGCAACAAGCACAACAAGCACAACAACAA 372

Gli-B2-5 CAGTATCCGCAACCACAACAACCAATTTCACAGCAACAAGCACAACAAGCACAACAACAA 372

Gli-B2-8 CAGTATCCGCAACCACAACAACCAATTTCACAGCAACAAGCACAACAAGCACAACAACAA 372

****** ******************** ***** **

Gli-B2-12 CAACAACAACA---------------------------ACAGCAACAACAACAACAACAA 423

Gli-B2-2 CA------------------------------------ACAACAACAACAACAACAACAA 414

Gli-B2-11 CA------------------------------------ACAACAACAACAACAACAACAA 414

Gli-B2-1 CA------------------------------------ACAACAACAACAAGAACAACAA 399

Gli-A2-17 CAAC------------------------------------AACAACAACAAGAACAACAA 396

Gli-A2-15 CAAC------------------------------------AACAACAACAAGAACAACAA 399

Gli-A2-8 CAAC------------------------------------AACAACAACAAGAACAACAA 399

Gli-A2-4 CAAC------------------------------------AACAACAACAAGAACAACAA 399

Gli-A2-16 CAAC------------------------------------AACAACAACAAGAACAACAA 399

Gli-A2-11 --------------------------------------------ACAACAACAACAACAA 390

Gli-A2-18 --------------------------------------------ACAACAACAACAACAA 390

Gli-A2-6 CAACAAC---AACAACA------------ACAACAACAACAACAACAACAAGAACAACAA 420

Gli-A2-13 --------------------------------------------ACAACAACAACAACAA 390

Gli-A2-14 --------------------------------------------ACAACAACAACAACAA 390

Gli-A2-1 --------------------------------------------ACAACAACAACAACAA 390

Gli-A2-2 --------------------------------------------ACAACAACAACAACAA 390

Gli-A2-12 --------------------------------------------ACAACAACAACAACAA 390

Gli-A2-3 CAACAACAACAACAACA------------ACAACAACAACAACAACAACAAGAACAACAA 423

Gli-A2-5 CAACAACAACAACAACA------------ACAACAACAACAACAACAACAAGAACAACAA 423

Gli-A2-7 CAACAACAACAACAACA------------ACAACAACAACAACAACAACAAGAACAACAA 423

Gli-A2-9 CAACAACAACAACAACA------------ACAACAACAACAACAACAACAAGAACAACAA 423

Gli-A2-10 CAACAACAACAACAACA------------ACAACAACAACAACAACAACAAGAACAACAA 423

Gli-D2-2 ------------------------------------CAACAACAACAACAACAACAACAA 381

Gli-D2-12 -----------ACA---------------ACAAAAACAACAACAACAACAACAACAACAG 450

Gli-D2-13 -----------ACAAAA------------ACAACAACAACAACAACAACAACAACAACAG 453

Gli-D2-14 -----------ACAAAA------------AC---AACAACGACAACAACAACAACAACAG 450

Gli-D2-15 -----------ACAAAA------------AC---AACAACAACAACAACAACAACAACAG 450

Gli-D2-18 -----------------------------------------ACAACAACAACAACAACAA 390

Gli-D2-16 -----------------------------------------ACAACAACAACAACAACAA 390

Gli-D2-17 -----------------------------------------ACAACAACAACAACAACAA 390

Gli-D2-19 ------------------------------------------CAACAACAACAACAACAG 408

Gli-D2-3 ------------------------------------------CAACAACAACAACAACAA 393

Gli-D2-11 ------------------------------------------CAACAACAACAACAACAG 396

Gli-D2-9 ------------------------------------------CAACAACAACAACAACAG 408

Gli-D2-10 ------------------------------------------CAACAACAACAACAACAG 408

Gli-D2-8 ------------------------------------------CAACAACAACAACAACAG 408

Gli-D2-7 ------------------------------------------CAACAACAACAACAACAG 405

Gli-D2-6 ------------------------------------------CAACAACAACAACAACAG 408

Gli-D2-4 ------------------------------------------CAACAACAACAACAACAG 408

Gli-D2-5 ------------------------------------------CAACAACAACAACAACAG 405

Gli-D2-1 ---------------------------------------------------CAACAACAA 381

Gli-B2-3 CAACAACAACAACAACAACAACAACAACAACAACAACAACAACAACAACAACAACAACAA 432

Gli-B2-9 CAACAACAACA---ACAACAACAACAACAACAACAACAACAACAACAACAACAACAACAA 321

Gli-B2-7 CAACAACAACAACAACAA------CAACAACAACAACAACAACAACAACAACAACAACAA 426

Gli-B2-10 CAACAACAACAACAACAACAACAACAACAACAACAACAACAACAACAACAACAACAACAA 432

Gli-B2-4 CAACAACAACAACAACAACAACAACAACAACAACAACAACAACAACAACAACAACAACAA 432

Gli-B2-6 CAACAACGACAACAACAACAACAACAACAACAACAACAACAACAACAACAACAACAACAA 432

Gli-B2-5 CAACAACAACAACAACAACAACAACAACAACAACAACAACAACAACAACAACAACAACAA 432

Gli-B2-8 CAACAACAACGACAACAACAACAACAACAACAACAACAACAACAACAACAACAACAACAA 432

*******

Gli-B2-12 CAAATCCTTCAACAAATTTTGCAACAACAACTGATTCCATGCAGGGATGTTGTCTTGCAA 483

Gli-B2-2 CAAATTCTTCAACAAATTTTGCAACAACAACTGATTCCATGCAGGGATGTTGTCTTGCAA 474

Gli-B2-11 CAAATTCTTCAACAAATTTTGCAACAACAACTGATTCCATGCAGGGATGTTGTCTTGCAA 474

Gli-B2-1 AT---CCTTCAACAAATTTTGCAACAACAACTGATTCCATGCATGGATGTTGTATTGCAG 456

Gli-A2-17 AT---CCTTCAACAAATTTTGCAACAACAACTGATTCCATGCATGGATGTTGTATTGCAG 453

Gli-A2-15 AT---CCTTCAACAAATTTTGCAACAACAACTGATTCCATGCATGGATGTTGTATTGCAG 456

Gli-A2-8 AT---CCTTCAACAAATTTTGCAACAACAACTGATTCCATGCATGGATGTTGTATTGCAG 456

Gli-A2-4 AT---CCTTCAACAAATTTTGCAACAACAACTGATTCCATGCATGGATGTTGTATTGCAG 456

Gli-A2-16 AT---CCTTCAACAAATTTTGCAACAACAACTGATTCCATGCATGGATGTTGTATTGCAG 456

Gli-A2-11 AT---CCTTCAACAAATTTTGCAACAACAACTGATTCCATGCATGGATGTTGTATTGCAG 447

Gli-A2-18 AT---CCTTCAACAAATTTTGCAACAACAACTGATTCCATGCATGGATGTTGTATTGCAG 447

Gli-A2-6 AT---CCTTCAACAAATTTTGCAACAACAACTGATTCCATGCATGGATGTTGTATTGCAG 477

Gli-A2-13 AT---CCTTCAACAAATTTTGCAACAACAACTGATTCCATGCATGGATGTTGTATTGCAG 447

Gli-A2-14 AT---CCTTCAACAAATTTTGCAACAACAACTGATTCCATGCATGGATGTTGTATTGCAG 447

Gli-A2-1 AT---CCTTCAACAAATTCTGCAACAACAACTGATTCCATGCATGGATGTTGTATTGCAG 447

Gli-A2-2 AT---CCTTCAACAAATTTTGCAACAACAACTGATTCCATGCATGGATGTTGTATTGCAG 447

Gli-A2-12 AT---CCTTCAACAAATTTTGCAACAACAACTGATTCCATGCATGGATGTTGTATTGCAG 447

Gli-A2-3 AT---CCTTCAACAAATTTTGCAACAACAACTGATTCCATGCATGGATGTTGTATTGCAG 480

Gli-A2-5 AT---CCGTCAACAAATTTTGCAACAACAACTGATTCCATGCATGGATGTTGTATTGCAG 480

Gli-A2-7 AT---CCTTCAACAAATTTTGCAACAACAACTGATTCCATGCATGGATGTTGTATTGCAG 480

Gli-A2-9 AT---CCTTCAACAAATTTTGCAACAACAACTGATTCCATGCATGGATGTTGTATTGCAG 480

Gli-A2-10 AT---CCTTCAACAAATTTTGCAACAACAACTGATTCCATGCATGGATGTTGTATTGCAG 480

Gli-D2-2 AT---CCTTCAACAAATTCTGCAACAACAACTGATTCCATGCAGGGATGTTGTCTTGCAA 438

Gli-D2-12 AT---CCTTCAACAAATTTTGCAACAACAACTGATTCCATGCAGGGATGTTGTATTGCAA 507

Gli-D2-13 AT---CCTTCAACAAATTTTGCAACAACAACTGATTCCATGCAGGGATGTTGTATTGCAA 510

Gli-D2-14 AT---CCTTCAACAAATTTTGCAACAACAACTGATTCCGTGCAGGGATGTTGTATTGCAA 507

Gli-D2-15 AT---CCTTCAACAAATTTTGCAACAACAACTGATTCCATGCAGGGATGTTGTATTGCAA 507

Gli-D2-18 AT---CCTACAACAAATTTTGCAACAACAACTGATTCCATGCAGGGATGTTGTATTGCAA 447

Gli-D2-16 AT---CCTACAACAAATTTTGCAACAACAACTGATTCCATGCAGGGATGTCGTATTGCAA 447

Gli-D2-17 AT---CCTACAACAAATTTTGCAACAACAACTGATTCCATGCAGGGATGTTGTATTGCAA 447

Gli-D2-19 AT---CCTTCAACAAATTTTGCAACAACAACTGATTCCATGCAGGGATGTTGTATTGCAA 465

Gli-D2-3 AT---CCTTCAACAAATTTTGCAACAACAACTGATTCCATGCAGGGATGTTGTATTGCAA 450

Gli-D2-11 AT---CCTTCAACAAATTTTGCAACAACAACTGATTCCATGCAGGGATGTTGTATTGCAA 453

Gli-D2-9 AT---CCTTCAACAAATTTTGCAACAACAACTGATTCCATGCAGGGATGTTGTATTGCAA 465

Gli-D2-10 AT---CCTTCAACAAATTTTGCAACAACAACTGATTCCATGCAGGGATGTTGTATTGCAA 465

Gli-D2-8 AT---CCTTCAACAAATTTTGCAACAACAACTGATTCCATGCAGGGATGTTGTATTGCAA 465

Gli-D2-7 AT---CCTTCAACAAATTTTGCAACAACAACTGATTCCATGCAGGGATGTTGTATTGCAA 462

Gli-D2-6 AT---CCTTCAACAAATTTTGCAACAACAACTGATTCCATGCAGGGATGTTGTATTGCAA 465

Gli-D2-4 AT---CCTTCAACAAATTTTGCAACAACAACTGATTCCATGCAGGGATGTTGTATTGCAA 465

Gli-D2-5 AT---CCTTCAACAAATTTTGCAACAACAACTGATTCCATGCAGGGATGTTGTATTGCAA 462

Gli-D2-1 ATCCTTCAACAA---ATTCTGCAACAACAACTGATTCCATGCAGGGATGTTGTCTTGCAA 438

Gli-B2-3 ATCCTTCAACAAATTCTGCAACAACAACAACTGATTCCATGCAGGGATGTCGTCTTGCAA 492

Gli-B2-9 ATCCTTCAACAAATTCTGCAACAACAACAACTGATTCCATGCAGGGATGTCGTCTTGCAA 381

Gli-B2-7 ATCCTTCAACAAATTCTGCAACAACAACAACTGATTCCATGCAGGGATGTCGTCTTGCAA 486

Gli-B2-10 ATCCTTCAACAAATTCTGCAACAACAACAACTGATTCCATGCAGGGATGTCGTCTTGCAA 492

Gli-B2-4 ATCCTTCAACAAATTCTGCAACAACAACAACTGATTCCATGCAGGGATGTCGTCTTGCAA 492

Gli-B2-6 ATCCTTCAACAAATTCTGCAACAACAACAACTGATTCCATGCAGGGATGTCGTCTTGCAA 492

Gli-B2-5 ATCCTTCAACAAATTCTGCAACAACAACAACTGATTCCATGCAGGGATGTCGTCTTGCAA 492

Gli-B2-8 ATCCTTCAACAAATTCTGCAACAACAACTACTGATTCCATGCAGGGATGTCGTCTTGCAA 492

* *** * ******* ********* **** ****** ** *****

Gli-B2-12 CAACACAACATAGCGCATGCAAGATCACAAGTTTTGCAACAAAGCACTTACCAGCCATTG 543

Gli-B2-2 CAACACTACATAGCGCATGCAAGCTCACAAGTTTTGCAACAAAGTACTTACCAGCTATTG 534

Gli-B2-11 CAACACAACATAGCGCATGCAAGCTCACAAGTTTTGCAACAAAGTACTTACCAGCTATTG 534

Gli-B2-1 CAACACAACATAGCGCATGGAACATCACAAGTTTTGCAACAAAGTACTTACCAGCTGTTG 516

Gli-A2-17 CAACACAACATAGCGCATGGAAGATCACAAGTTTTGCAACAAAGTACTTACCAGCTGTTG 513

Gli-A2-15 CAACACAACATAGCGCATGGAAGATCACAAGTTTTGCAACAAAGTACTTACCAGCTGTTG 516

Gli-A2-8 CAACACAACATAGCGCATGGAAGATCACAAGTTTTGCAACAAAGTACTTACCAGCTGTTG 516

Gli-A2-4 CAACACAACATAGCGCATGGAAGATCACAAGTTTTGCAACAAAGTACTTACCAGCTGTTG 516

Gli-A2-16 CAACACAACATAGCGCATGGAAGATCACAAGTTTTGCAACAAAGTACTTACCAGCTGTTG 516

Gli-A2-11 CAACACAACATAGCGCATGGAAGATCACAAGTTTTGCAACAAAGTACTTACCAGCTGTTG 507

Gli-A2-18 CAACACAACATAGCGCATGGAAGATCACAAGTTTTGCAACAAAGTACTTACCAGCTGTTG 507

Gli-A2-6 CAACACAACATAGCGCATGGAAGATCACAAGTTTTGCAACAAAGTACTTACCAGCTGTTG 537

Gli-A2-13 CAACACAACATAGCGCATGGAAGATCACAAGTTTTGCAACAAAGTACTTACCAGCTGTTG 507

Gli-A2-14 CAACACAACATAGCGCATGGAAGATCACAAGTTTTGCAACAAAGTACTTACCAGCTGTTG 507

Gli-A2-1 CAACACAACATAGCGCATGGAAGATCACAAGTTTTGCAACAAAGTACTTACCAGCTGTTG 507

Gli-A2-2 CAACACAACATAGCGCATGGAAGATCACAAGTTTTGCAACAAAGTACTTACCAGCTGTTG 507

Gli-A2-12 CAACACAACATAGCGCATGGAAGATCACAAGTTTTGCAACAAAGTACTTACCAGCTGTTG 507

Gli-A2-3 CAACACAACATAGCGCATGGAAGATCACAAGTTTTGCAACAAAGTACTTACCAGCTGTTG 540

Gli-A2-5 CAACACAACATAGCGCATGGAAGATCACAAGTTTTGCAACAAAGTACTTACCAGCTGTTG 540

Gli-A2-7 CAACACAACATAGCGCATGGAAGATCACAAGTTTTGCAACAAAGTACTTACCAGCTGTTG 540

Gli-A2-9 CAACACAACATAGCGCATGGAAGATCACAAGTTTTGCAACAAAGTACTTACCAGCTGTTG 540

Gli-A2-10 CAACACAACATAGCGCATGGAAGATCACAAGTTTTGCAACAAAGTACTTACCAGCTGTTG 540

Gli-D2-2 CAACACAACATAGCGCATGCAAGCTCACAAGTACTGCAACAAAGTACTTACCAGCTGGTG 498

Gli-D2-12 CAACACAGCATAGCGTATGGAAGCTCACAAGTTTTGCAACAAAGTACTTACCAGCTGGTG 567

Gli-D2-13 CAACACAGCATAGCGTATGGAAGCTCACAAGTTTTGCAACAAAGTACTTACCAGCTGGTG 570

Gli-D2-14 CAACACAGCATAGCGTATGGAAGCTCACAAGTTTTGCAACAAAGTACTTACCAGCTGGTG 567

Gli-D2-15 CAACACAGCATAGCGTATGGAAGCTCACAAGTTTTGCAACAAAGTACTTACCAGCTGGTG 567

Gli-D2-18 CAACACAACATAGCGCATGGAAGCTCACAAGTTTTGCAAGAAAGTACTTACCAGCTGGTG 507

Gli-D2-16 CAACACAACATAGCGCATGGAAGCTCACAAGTTTTGCAAGAAAGTACTTACCAGCTGGTG 507

Gli-D2-17 CAACACAACATAGCGCATGGAAGCTCACAAGTTTTGCAAGAAAGTACTTACCAGCTGGTG 507

Gli-D2-19 CAACACAGCATAGCGCATGGAAGCTCACAAGTTTTGCAACAAAGTACTTACCAGCTGGTG 525

Gli-D2-3 CAACACAACATAGCGCATGGAAGGTCACAAGTTTTGCAACAAAGTACTTACCAGCTGGTG 510

Gli-D2-11 CAACACAGCATAGCGCATGGAAGCTCACAAGTTTTGCAACAAAGTACTTACCAGCTGGTG 513

Gli-D2-9 CAACACAGCATAGCGCATGGAAGCTCACAAGTTTTGCAACAAAGTACTTACCAGCTGGTG 525

Gli-D2-10 CAACACAGCATAGCGTATGGAAGCTCACAAGTTTTGCAACAAAGTACTTACCAGCTGGTG 525

Gli-D2-8 CAACACAGCATAGCGCATGGAAGCTCACAAGTTTTGCAACAAAGTACTTACCAGCTGGTG 525

Gli-D2-7 CAACACAGCATAGCGCATGGAAGCTCACAAGTTTTGCAACAAAGTACTTACCAGCTGGTG 522

Gli-D2-6 CAACACAGCATAGCGCATGGAAGCTCACAAGTTTTGCAACAAAGTACTTACCAGCTGGTG 525

Gli-D2-4 CAACACAGCATAGCGCATGGAAGCTCACAAGTTTTGCAACAAAGTACTTACCAGCTGGTG 525

Gli-D2-5 CAACACAGCATAGCGCATGGAAGCTCACAAGTTTTGCAACAAAGTACTTACCAGCTGGTG 522

Gli-D2-1 CAACACAACATAGCGCATGCAAGCTCACAAGTATTGCAACAAAGTAGTTACCAACAGTTG 498

Gli-B2-3 CAACACAACATAGCGCATGCAAGCTCACAAGTATTGCAACAAAGTAGTTACCAACTGTTG 552

Gli-B2-9 CAACACAACATAGCGCATGCAAGCTCACAAGTATTGCAACAAAGTAGTTACCAACTGTTG 441

Gli-B2-7 CAACACAACATAGCGCATGCAAGCTCACAAGTATTGCAACAAAGTAGTTACCAACTGTTG 546

Gli-B2-10 CAACACAACATAGCGCATGCAAGCTCACAAGTATTGCAACAAAGTAGTTACCAACTGTTG 552

Gli-B2-4 CAACACAACATAGCGCATGCAAGCTCACAAGTATTGCAACAAAGTAGTTACCAACTGTTG 552

Gli-B2-6 CAACACAACATAGCGCATGCAAGCTCACAAGTATTGCAACAAAGTAGTTACCAACTGTTG 552

Gli-B2-5 CAACACAACATAGCGCATGCAAGCTCACAAGTATTGCAACAAAGTAGTTACCAACTGTTG 552

Gli-B2-8 CAACACAACATAGCGCATGCAAGCTCACAAGTATTGCAACAAAGTAGTTACCAACTGTTG 552

****** ******* *** ** ******** ***** **** * ****** * **

Gli-B2-12 CAACAATTGTGTTGTCAACAGCTGTGGCAGATCCCCGAGCAGTCGCGGTGCCAAGCCATC 603

Gli-B2-2 CAACAATTGTGTTGTCAACAACTGTTGCAGATCCCTGAGCAGTCGAGGTGCCAAGCCATC 594

Gli-B2-11 CAACAATTGTGTTGTCAACAACTGTTGCAGATCCCTGAGCAGTCGAGGTGCCAAGCCATC 594

Gli-B2-1 CAAGAATTGTGTTGTCAGCACCTATGGCAGATCCCTGAGCAGTCGCAGTGCCAGGCCATC 576

Gli-A2-17 CAAGAATTGTGTTGTCAGCACCTATGGCAGATCCCTGAGCAGTCGCAGTGCCAGGCCATC 573

Gli-A2-15 CAAGAATTGTGTTGTCAGCACCTATGGCAGATCCCTGAGCAGTCGCAGTGCCAGGCCATC 576

Gli-A2-8 CAAGAATTGTGTTGTCAGCACCTATGGCAGATCCCTGAGCAGTCGCAGTGCCAGGCCATC 576

Gli-A2-4 CAAGAATTGTGTTGTCAGCACCTATGGCAGATCCCTGAGCAGTCGCAGTGCCAGGCCATC 576

Gli-A2-16 CAAGAATTGTGTTGTCAGCACCTATGGCAGATCCCTGAGCAGTCGCAGTGCCAGGCCATC 576

Gli-A2-11 CAAGGATTGTGTTGTCAGCACCTATGGCAGATCCCTGAGCAGTCGCAGTGCCAGGCCATC 567

Gli-A2-18 CAAGAATTGTGTTGTCAGCACCTATGGCAGATCCCTGAGCAGTCGCAGTGCCAGGCCATC 567

Gli-A2-6 CAAGAATTGTGTTGTCAGCACCTATGGCAGATCCCTGAGCAGTCGCAGTGCCAGGCCATC 597

Gli-A2-13 CAAGAATTGTGTTGTCAGCACCTATGGCAGATCCCTGAGCAGTCGCAGTGCCAGGCCATC 567

Gli-A2-14 CAAGAATTGTGTTGTCAACACCTATGGCAGATCCCTGAGCAGTCGCAGTGCCAGGCCATC 567

Gli-A2-1 CAAGAATTGTGTTGTCAACACCTATGGCAGATCCCTGAGCAGTCGCAGTGCCAGGCCATC 567

Gli-A2-2 CAAGAATTGTGTTGTCAACACCTATGGCAGATCCCTGAGCAGTCGCAGTGCCAGGCCATC 567

Gli-A2-12 CAAGAATTGTGTTGTCAACACCTATGGCAGATCCCTGAGCAGTCGCAGTGCCAGGCCATC 567

Gli-A2-3 CAAGAATTGTGTTGTCAGCACCTATGGCAGATCCCTGAGCAGTCGCAGTGCCAGGCCATC 600

Gli-A2-5 CAAGAATTGTGTTGTCAGCACCTATGGCAGATCCCTGAGCAGTCGCAGTGCCAGGCCATC 600

Gli-A2-7 CAAGAATTGTGTTGTCAGCACCTATGGCAGATCCCTGAGCAGTCGCAGTGCCAGGCCATC 600

Gli-A2-9 CAAGAATTGTGTTGTCAGCACCTATGGCAGATCCCTGAGCAGTCGCAGTGCCAGGCCATC 600

Gli-A2-10 CAAGAATTGTGTTGTCAGCACCTATGGCAGATCCCTGAGCAGTCGCAGTGCCAGGCCATC 600

Gli-D2-2 CAACAATTGTGTTGTCAGCAGCTGTGGCAGATCCCCGAGCAGTCGCGGTGCCAAGCCATC 558

Gli-D2-12 CAACAATTGTGTTGTCAGCAGCTGTGGCAGATCCCCGAGCAGTCGCGGTGCCAAGCCATC 627

Gli-D2-13 CAACAATTGTGTTGTCAGCAGCTGTGGCAGATCCCCGAGCAGTCGCGGTGCCAAGCCATC 630

Gli-D2-14 CAACAATTGTGTTGTCAGCAGCTGTGGCAGATCCCCGAGCAGTCGCGGTGCCAAGCCATC 627

Gli-D2-15 CAACAATTGTGTTGTCAGCAGCTGTGGCAGATCCCCGAGCAGTCGCGGTGCCAAGCCATC 627

Gli-D2-18 CAACAATTGTGTTGTCAGCAGCTGTGGCAGATCCCCGAGCAGTCGCGGTGCCAAGCCATC 567

Gli-D2-16 CAACAATTGTGTTGTCAGCAGCTGTGGCAGATCCCCGAGCAGTCGCGGTGCCAAGCCATC 567

Gli-D2-17 CAACAATTGTGTTGTCAGCAGCTGTGGCAGATCCCCGAGCAGTCGCGGTGCCAAGCCATC 567

Gli-D2-19 CAACAATTGTGTTGTCAGCAGCTGTGGCAGATCCCCGAGCAGTCGCGGTGCCAAGCCATC 585

Gli-D2-3 CAACAATTGTGTTGTCAGCAGCTGTGGCAGATCCCCGAGCAGTCGCGGTGCCAAGCCATC 570

Gli-D2-11 CAACAATTGTGTTGTCAGCAGCTGTGGCAGATCCCCGAGCAGTCGCGGTGCCAAGCCATC 573

Gli-D2-9 CAACAATTGTGTTGTCAGCAGCTGTGGCAGATCCCCGAGCAGTCGCGGTGCCAAGCCATC 585

Gli-D2-10 CAACAATTGTGTTGTCAGCAGCTGTGGCAGATCCCCGAGCAGTCGCGGTGCCAAGCCATC 585

Gli-D2-8 CAACAATTGTGTTGTCAGCAGCTGTGGCAGATCCCCGAGCAGTCGCGGTGCCAAGCCATC 585

Gli-D2-7 CAACAATTGTGTTGTCAGCAGCTGTGGCAGATCCCCGAGCAGTCGCGGTGCCAAGCCATC 582

Gli-D2-6 CAACAATTGTGTTGTCAGCAGCTGTGGCAGATCCCCGAGCAGTCGCGGTGCCAAGCCATC 585

Gli-D2-4 CAACAATTGTGTTGTCAGCAGCTGTGGCAGATCCCCGAGCAGTCGCGGTGCCAAGCCATC 585

Gli-D2-5 CAACAATTGTGTTGTCAGCAGCTGTGGCAGATCCCCGAGCAGTCGCGGTGCCAAGCCATC 582

Gli-D2-1 CAACAATTATGTTGTCAGCAACTGTTTCAGATCCCCGAGCAGTCGCGGTGCCAAGCCATC 558

Gli-B2-3 CAACAATTATGTTGTCAACGTTTGTGGCAGATCCCCGAGCAGTCGCGGTGCCAAGCCATC 612

Gli-B2-9 CAACAATTATGTTGTCAACGTTTGTGGCAGATCCCCGAGCGGTCGCGGTGCCAAGCCATC 501

Gli-B2-7 CAACAATTATGTTGTCAACGTTTGTGGCAGATCCCCGAGCAGTCGCGGTGCCAAGCCATC 606

Gli-B2-10 CAACAATTATGTTGTCAACGTTTGTGGCAGATCCCCGAGCAGTCGCGGTGCCAAGCCATC 612

Gli-B2-4 CAACAATTATGTTGTCAACGTTTGTGGCAGATCCCCGAGCAGTCGCGGTGCCAAGCCATC 612

Gli-B2-6 CAACAATTATGTTGTCAACGTTTGTGGCAGATCCCCGAGCAGTCGCGGTGCCAAGCCATC 612

Gli-B2-5 CAACAATTATGTTGTCAACGTTTGTGGCAGATCCCCGAGCAGTCGCGGTGCCAAGCCATC 612

Gli-B2-8 CAACAATTATGTTGTCAACGTTTGTGGCAGATCCCCGAGCAGTCGCGGTGCCAAGCCATC 612

*** *** ******** * * * ******** **** **** ****** ******

Gli-B2-12 CACAATGTTGTTCATGCTATTATTCTGCATCAACAACAGCGACA---------------- 647

Gli-B2-2 CATAATGTTGCTCATGCTATTATTATGCAACAACAACAACAACAACAACAACAACAACAA 654

Gli-B2-11 CATAATGTTGCTCATGCTATTATTATGCATCAACAACAACAACAACAACAAGAACAAAAA 654

Gli-B2-1 CACAATGTTGTTCATGCTATTATTCTGCATCAACAACAACAACA---------------- 620

Gli-A2-17 CACAATGTTGTTCATGCTATTATTCTGCATCAACAACAAAAACA---------------- 617

Gli-A2-15 CACAATGTTGTTCATGCTATTATTCTGCATCAACAACAAAAACA---------------- 620

Gli-A2-8 CACAATGTTGTTCATGCTATTATTCTGCATCAACAACAAAAACA---------------- 620

Gli-A2-4 CACAATGTTGTTCATGCTATTATTCTGCATCAACAACAAAAACA---------------- 620

Gli-A2-16 CACAATGTTGTTCATGCTATTATTCTGCATCAACAACAAAAACA---------------- 620

Gli-A2-11 CACAATGTTGTTCATGCTATTATTCTGCATCAACAACAAAAACA---------------- 611

Gli-A2-18 CACAATGTTGTTCATGCTATTATTCTGCATCAACAACAAAAACA---------------- 611

Gli-A2-6 CACAATGTTGTTCATGCTATTATTCTGCATCAACAACAAAAACA---------------- 641

Gli-A2-13 CACAATGTTGTTCATGCTATTATTCTGCATCAACAACAAAAACA---------------- 611

Gli-A2-14 CACAATGTTGTTCATGCTATTATTCTGCATCAACAACAAAAACA---------------- 611

Gli-A2-1 CACAATGTTGTTCATGCAATTATTCTGCATCAACAACAAAAACA---------------- 611

Gli-A2-2 CACAATGTTGTTCATGCTATTATTCTGCATCAACAACAAAAACA---------------- 611

Gli-A2-12 CACAATGTTGTTCATGCTATTATTCTGCATCAACAACAAAAACA---------------- 611

Gli-A2-3 CACAATGTTGTTCATGCTATTATTCTGCATCAACAACAAAAACA---------------- 644

Gli-A2-5 CACAATGTTGTTCATGCTATTATTCTGCATCAACAACAAAAACA---------------- 644

Gli-A2-7 CACAATGTTGTTCATGCTATTATTCTGCATCAACAACAAAAACA---------------- 644

Gli-A2-9 CACAATGTTGTTCATGCTATTATTCTGCATCAACAACAAAAACA---------------- 644

Gli-A2-10 CACAATGTTGTTCATGCTATTATTCTGCATCAACAACAAAAACA---------------- 644

Gli-D2-2 CACAATGTTGTTCATGCTATTATTCTGCATCAACAACACCACCACCACCAACAAC----- 613

Gli-D2-12 CACAATGTTGTTCATGCTATTATTCTGCATCAACAGCAACAACAACA------------- 674

Gli-D2-13 CACAATGTTGTTCATGCTATTATTCTGCATCAACAGCAACAACAACA------------- 677

Gli-D2-14 CACAATGTTGTTCATGCTATTATTCTGCATCAACAGCAACAACAACA------------- 674

Gli-D2-15 CACAATGTTGTTCATGCTATTATTCTGCATCAACAGCAACAACAACA------------- 674

Gli-D2-18 CACAATGTTGTTCATGCTATTATTCTGCATCAACAACACCACCACCACCAACAA------ 621

Gli-D2-16 CACAATGTTGTTCATGCTATTATTCTGCATCAACAACACCACCACCACCAACAA------ 621

Gli-D2-17 CACAATGTTGTTCATGCTATTATTCTGCATCAACAACACCACCACCACCAACAA------ 621

Gli-D2-19 CACAATGTTGTTCATGCTATTATTCTGCATCAACAACACCACCACCACCGACAA------ 639

Gli-D2-3 CACAATGTTGTTCATGCTATTATTCTGCATCATCATCAACAACAA--------------- 615

Gli-D2-11 CACAATGTTGTTCATGCTATTATTCTGCATCAACAACAACAACAACGACA---------- 623

Gli-D2-9 CACAATGTTGTTCATGCTATTATTCTGCATCAACAGCAACAACAACAACA---------- 635

Gli-D2-10 CACAATGTTGTTCATGCTATTATTCTGCATCAACAGCAACAACAACAA------------ 633

Gli-D2-8 CACAATGTTGTTCATGCTATTATTCTGCATCAGCAACAACAACA---------------- 629

Gli-D2-7 CACAATGTTGTTCATGCTATTATTCTGCATCAACAACAACAACAACAACAACAACAACA- 641

Gli-D2-6 CACAATGTTGTTCATGCTATTATTCTGCATCAACAACAACAACA---------------- 629

Gli-D2-4 CACAATGTTGTTCATGCTATTATTCTGCATCAACAACAACAACA---------------- 629

Gli-D2-5 CACAATGTTGTTCATGCTATTATTCTGCATCAACAACAACAACA---------------- 626

Gli-D2-1 CACAATGTCGTTCATGCTATTATTCTGCATCATCATCAACAA------------------ 600

Gli-B2-3 CACAATGTCGTTCATGCTATTACTCTGCAACAACAACAACAACAACAACAACAACAACAA 672

Gli-B2-9 CACAATGTCGTTCATGCTATTATTCTGCAACAACAACAACAACAACAACAACAACAACAA 561

Gli-B2-7 CACAATGTCGTTCATGCTATTATTCTGCAACAACAACAACAACAACAACAACAACAACAA 666

Gli-B2-10 CACAATGTCGTTCATGCTATTATTCTGCAACAACAACAACAACAACAACAACAACAACAA 672

Gli-B2-4 CACAATGTCGTTCATGCTATTATTCTGCAACAACAACAACAACAACAACAACAACAACAA 672

Gli-B2-6 CACAATGTCGTTCATGCTATTATTCTGCAACAACAACAACAACAACAACAACAGCAACAA 672

Gli-B2-5 CACAATGTCGTTCATGCTATTATTCTGCAACAACAACAACAACAACAACAACAACAACAA 672

Gli-B2-8 CACAATGTCGTTCATGCTATTATTCTGCAACAACAACAACAACAACAACAACAACAACAA 672

** ***** * ****** **** * **** ** ** **

Gli-B2-12 ------------------------------------------------------------ 647

Gli-B2-2 CAACA---------------------ACAACAACAACAACAACAACAACAACAACAACAA 693

Gli-B2-11 CAACAGTTGCAACAACAACAACAACAACAACAGCAACTGCAACAACAACAACAACAACAA 714

Gli-B2-1 --------------------------------------------------------ACAA 624

Gli-A2-17 -----------------------------------------------------------A 618

Gli-A2-15 -----------------------------------------------------------A 621

Gli-A2-8 -----------------------------------------------------------A 621

Gli-A2-4 -----------------------------------------------------------A 621

Gli-A2-16 -----------------------------------------------------------A 621

Gli-A2-11 -----------------------------------------------------------A 612

Gli-A2-18 -----------------------------------------------------------A 612

Gli-A2-6 -----------------------------------------------------------A 642

Gli-A2-13 -----------------------------------------------------------A 612

Gli-A2-14 -----------------------------------------------------------A 612

Gli-A2-1 -----------------------------------------------------------A 612

Gli-A2-2 -----------------------------------------------------------A 612

Gli-A2-12 -----------------------------------------------------------A 612

Gli-A2-3 -----------------------------------------------------------A 645

Gli-A2-5 -----------------------------------------------------------A 645

Gli-A2-7 -----------------------------------------------------------A 645

Gli-A2-9 -----------------------------------------------------------A 645

Gli-A2-10 -----------------------------------------------------------A 645

Gli-D2-2 ----------------------------------------------AACAACAACAACAA 627

Gli-D2-12 -----------------------------------------------ACA---ACAACAA 684

Gli-D2-13 -----------------------------------------------ACAACAACAACAA 690

Gli-D2-14 -----------------------------------------------ACAACAACAACAA 687

Gli-D2-15 -----------------------------------------------ACAACAACAACAA 687

Gli-D2-18 ---------------------------------------------CAACAACAACAACAA 636

Gli-D2-16 ---------------------------------------------CAACAACAACAACAA 636

Gli-D2-17 ---------------------------------------------CAACAACAACAACAA 636

Gli-D2-19 ---------------------------------------------CAACAACAACAACAA 654

Gli-D2-3 ------------------------------------------------------CAACAA 621

Gli-D2-11 -----------------------------------------------------ACAACAA 630

Gli-D2-9 -----------------------------------------------------ACAACAA 642

Gli-D2-10 ------------------------------------------------------CAACAA 639

Gli-D2-8 --------------------------------------------------------ACAA 633

Gli-D2-7 --------------------------------------------ACAACAACAACAACAA 657

Gli-D2-6 --------------------------------------------------------ACAA 633

Gli-D2-4 --------------------------------------------------------ACAA 633

Gli-D2-5 --------------------------------------------------------ACAA 630

Gli-D2-1 ---------------------------------------------------------CAA 603

Gli-B2-3 CAACAAC---------------------AACAACAACAACAACAACAACAACAACAACAA 711

Gli-B2-9 CAACAAC---------------------AACAACAACAACAACAACAACAACAACAACAA 600

Gli-B2-7 CAACAAC---------------------A---ACAACAACAACAACAACAACAACAACAA 702

Gli-B2-10 CAACAAC---------------------A---ACAACAACAACAACAACAACAACAACAA 708

Gli-B2-4 CAACA---------------------------ACAACAACAACAACAACAACAACAACAA 705

Gli-B2-6 CAACAAC---------------------AACAACAACAACAACAGCAACAACAACAACAA 711

Gli-B2-5 CAACAAC---------------------AACAACAACAACAACAACAACAACAACAACAA 711

Gli-B2-8 CAACAAC---------------------AACAACAACAACAACAACAACAACAACAACAA 711

Gli-B2-12 --ACAACAACCATCGAGCCAGGTCTCCTTGCAACAGCCTCAGCAACAATATCCATCGGGC 705

Gli-B2-2 CAACAACAACCGTCGAGCCAGGTCTCCTACCAGCAGCCTCAGCAACAATATCCATCGGGC 753

Gli-B2-11 CAACAACAACCGTCGAGCCAGGTCTCCTTCCAACAGCCTCAGCAGCAATATCCATCAAGC 774

Gli-B2-1 CAACAACAACCGTTGAGCCAGGTCTGCTTCCAACAGTCTCAACAACAATATCCATCAGGC 684

Gli-A2-17 CAACAACAACCATCGAGCCAGTTCTCCTTCCAACAGCCTCTGCAACAATATCCGTTAGGC 678

Gli-A2-15 CAACAACAACCATCGAGCCAGTTCTCCTTCCAACAGCCTCTGCAACAATATCCGTTAGGC 681

Gli-A2-8 CAACAACAACCATCGAGCCAGGTCTCCTTCCAACAGCCTCTGCAACAATATCCATTAGGC 681

Gli-A2-4 CAACAACAACCATCGAGCCAGTTCTCCTTCCAACAGCCTCTGCAACAATATCCGTTAGGC 681

Gli-A2-16 CAACAACAACCATCGAGCCAGTTCTCCTTCCAACAGCCTCTGCAACAATATCCGTTAGGC 681

Gli-A2-11 CAGCAACAACCATCGAGCCAGTTCTCCTTCCAACAGCCTCTGCAACAATATCCGTTAGGC 672

Gli-A2-18 CAACAACAACCATCGAGCCAGTTCTCCTTCCAACAGCCTCTGCAACAATATCCGTTAGGC 672

Gli-A2-6 CAACAACAACCATCGAGCCAGGTCTCCTTCCAACAGCCTCTGCAACAATATCCATTAGGC 702

Gli-A2-13 CAACAACAACCATCGAGCCAGGTCTCCTTCCAACAGCCTCTGCAACAATATCCATTAGGC 672

Gli-A2-14 CAACAACAACCATCGAGCCAGGTCTCCTTCCAACAGCCTCTGCAACAATATCCATTAGGC 672

Gli-A2-1 CAACAACAACCATCGAGCCAGGTCTCCTTCCAACAGCCTCTGCAACAATATCCATTAGGC 672

Gli-A2-2 CAACAACAACCATCGAGCCAGGTCTCCTTCCAACAGCCTCTGCAACAATATCCATTAGGC 672

Gli-A2-12 CAACAACAACCATCGAGCCAGGTCTCCTTCCAACAGCCTCTGCAACAATATCCATTAGGC 672

Gli-A2-3 CAACAACAACCATCGAGCCAGGTCTCCTTCCAACAGCCTCTGCAACAATATCCATTAGGC 705

Gli-A2-5 CAACAACAACCATCGAGCCAGTTCTCCTTCCAACAGCCTCTGCAACAATATCCGTTAGGC 705

Gli-A2-7 CAACAACAACCATCGAGCCAGGTCTCCTTCCAACAGCCTCTGCAACAATATCCATTAGGC 705

Gli-A2-9 CAACAACAACCATCGAGCCAGGTCTCCTTCCAACAGCCTCTGCAACAATATCCATTAGGC 705

Gli-A2-10 CAACAACAACCATCGAGCCAGGTCTCCTTCCAACAGCCTCTGCAACAATATCCATTAGGC 705

Gli-D2-2 CAACAACAACCGTTGAGCCAAGTCTCCTTCCAACAGCCTCAGCAACAATATCCATCAGGC 687

Gli-D2-12 CAACAACAACCGTTGAGCCAGGTCTCCTTCCAACAGCCTCAACAACAATATCCATCAGGC 744

Gli-D2-13 CAAAAACAACCATTGAGCCAGGTCTCCTTCCAACAGCCTCAACAACAATATCCATCAGGC 750

Gli-D2-14 CAAAAACAACCATTGAGCCAGGTCTCCTTCCAACAGCCTCAACAACAATATCCATCAGGC 747

Gli-D2-15 CAAAAACAACCATTGAGCCAGGTCTCCTTCCAACAGCCTCAACAACAATATCCATCAGGC 747

Gli-D2-18 CAACAACAACCGTTGAGCCAAGTCTCCTTCCAACAGCCTCAGCAACAATATCCATCAGGC 696

Gli-D2-16 CAACAACAACCGTTGAGCCAAGTCTCCTTCCAACAGCCTCAGCAACAATATCCATCAGGC 696

Gli-D2-17 CAACAACAACCGTTGAGCCAAGTCTCCTTCCAACAGCCTCAGCAACAATATCCATCAGGC 696

Gli-D2-19 CAACAACAACCGTTGAGCCAAGTCTCCTTCCAACAGCCTCAGCAACAATATCCATCAGGC 714

Gli-D2-3 CAACAACAACCGTTGACCCAGGTCTCCTTCCAACAGCCTCAACAACAATATCCATCAGGC 681

Gli-D2-11 CAACAGCAACCGTTGAGCCAGGTCTCCTTCCAACAGCCTCAACAACAATATCCATCAGGC 690

Gli-D2-9 CAACAACAACCGTTGAGCCAGGTCTCCTTCCAACAGCCTCAACAACAATATCCATCAGGC 702

Gli-D2-10 CAACAACAACCGTTGAGCCAGGTCTCCTTCCAACAGCCTCAACAACAATATCCATCAGGC 699

Gli-D2-8 CAACAACAACCGTTGAGCCAGGTCTGCTTTCAACAGTCTCAACAACAATATCCATCAGGC 693

Gli-D2-7 CAACAACAACCGTTGAGCCAGGTCTGCTTCCAACAGTCTCAACAACAATATCCATCAGGC 717

Gli-D2-6 CAACAACAACCGTTGAGCCAGGTCTGCTTCCAACAGTCTCAACAACAATATCCATCAGGC 693

Gli-D2-4 CAACAACAACCGTTGAGCCAGGTCTGCTTCCAACAGTCTCAACAACAATATCCATCAGGC 693

Gli-D2-5 CAACAACAACCGTTGAGCCAGGTCTGCTTCCAACAGTCTCAACAACAATATCCATCAGGC 690

Gli-D2-1 CAACAACAACCGTCGAGCCAGGTCTCCTACCAGCAGCCTCAGGAACAATATCCATCAGGC 663

Gli-B2-3 CAACAACAACCGTCGAGCCAGGTCTCCTACCAGCAGCCTCAGCAACAATATCCATCGGGC 771

Gli-B2-9 CAACAACAACCGTCGAGCCAGGTCTCCTACCAGCAGCCTCAGCAACAATATCCATCGGGC 660

Gli-B2-7 CAACAACAACCGTCGAGCCAGGTCTCCTACCAGCAGCCTCAGCAACAATATCCATCGGGC 762

Gli-B2-10 CAACAACAACCGTCGAGCCAGGTCTCCTACCAGCAGCCTCAGCAACAATATCCATCGGGC 768

Gli-B2-4 CAACAACAACCGTCGAGCCAGGTCTCCTACCAGCAGCCTCAGCAACAATATCCATCGGGC 765

Gli-B2-6 CAACAACAACCGTCGAGCCAGGTCTCCTACCAGCAGCCTCAGCAACAATATCCATCGGGC 771

Gli-B2-5 CAACAACAACCGTCGAGCCAGGTCTCCTACCAGCAGCCTCAGCAACAATATCCATCGGGC 771

Gli-B2-8 CAACAACAACCGTCGAGCCAGGTCTCCTACCAGCAGCCTCAGCAACAATATCCATCGGGC 771

* ***** * ** *** *** ** ** *** *** * ******** * **

Gli-B2-12 CAGGGATCCTTCCAGCCATCTCAGCAGAACCCACAGGCCCAGGGCTCTGTCCAGCCTCAA 765

Gli-B2-2 CAGGGATCCTTCCAGCCATCTCAGCAGAACCCACAGGCCCAGGGCTCTGTCCAGCCTCAA 813

Gli-B2-11 CAGGTCTCCTTCCAGCCATCTCAGCTAAACCCACAGGCTCAGGGCTCTGTCCAACCTCAA 834

Gli-B2-1 CAGGGCTCCTTCCAGCCATCTCAGCAAAACCCACAGGCCCAGGGCTCTGTCCAGCCTCAA 744

Gli-A2-17 CAGGGCTCCTTCCGGCCATCTCAGCAAAACCCACAGGCCCAGGGCTCTGTCCAGCCTCAA 738

Gli-A2-15 CAGGGCTCCTTCCGGCCATCTCAGCAAAACCCACAGGCCCAGGGCTCTGTCCAGCCTCAA 741

Gli-A2-8 CAGGGCTCCTTCCGGCCATCTCAGCAAAACCCACAGGCCCAGGGCTCTGTCCAGCCTCAA 741

Gli-A2-4 CAGGGCTCCTTCCGGCCATCTCAGCAAAACCCACAGGCCCAGGGCTCTGTCCAGCCTCAA 741

Gli-A2-16 CAGGGCTCCTTCCGGCCATCTCAGCAAAACCCACAGGCCCAGGGCTCTGTCCAGCCTCAA 741

Gli-A2-11 CAGGGCTCCTTCCGGCCATCTCAGCAAAACCCACAGGCCCAGGGCTCTGTCCAGCCTCAA 732

Gli-A2-18 CAGGGCTCCTTCCGGCCATCTCAGCAAAACCCACAGGCCCAGGGCTCTGTCCAGCCTCAA 732

Gli-A2-6 CAGGGCTCCTTCCGGCCATCTCAGCAAAACCCACAGGCCCAGGGCTCTGTCCAGCCTCAA 762

Gli-A2-13 CAGGGCTCCTTCCGGCCATCTCAGCAAAACCCACAGGCCCAGGGCTCTGTCCAGCCTCAA 732

Gli-A2-14 CAGGGCTCCTTCCGGCCATCTCAGCAAAACCCACAGGCCCAGGGCTCTGTCCAGCCTCAA 732

Gli-A2-1 CAGGGCTCCTTCCGGCCATCTCAGCAAAACCCACAGGCCCAGGGCTCTGTCCAGCCTCAA 732

Gli-A2-2 CAGGGCTCCTTCCGGCCATCTCAGCAAAACCCACAGGCCCAGGGCTCTGTCCAGCCTCAA 732

Gli-A2-12 CAGGGCTCCTTCCGGCCATCTCAGCAAAACCCACAGGCCCAGGGCTCTGTCCAGCCTCAA 732

Gli-A2-3 CAGGGCTCCTTCCGGCCATCTCAGCAAAACCCACAGGCCCAGGGCTCTGTCCAGCCTCAA 765

Gli-A2-5 CAGGGCTCCTTCCGGCCATCTCAGCAAAACCCAGAGGCCCAGGGCTCTGTCCAGCCTCAA 765

Gli-A2-7 CAGGGCTCCTTCCGGCCATCTCAGCAAAACCCACAGGCCCAGGGCTCTGTCCAGCCTCAA 765

Gli-A2-9 CAGGGCTCCTTCCGGCCATCTCAGCAAAACCCACAGGCCCAGGGCTCTGTCCAGCCTCAA 765

Gli-A2-10 CAGGGCTCCTTCCGGCCATCTCAGCAAAACCCACAGGCCCGGGGCTCTGTCCAGCCTCAA 765

Gli-D2-2 CAGGGCTTCTTCCAACCATCTCAGCAAAACCCACAGGCCCAGGGCTCTTTCCAGCCTCAA 747

Gli-D2-12 CAGGGCTCCTTCCAGCCATCTCAGCAAAACCCACAGGCCCAGGGCTCTGTCCAGCCTCAA 804

Gli-D2-13 CAGGGCTCCTTCCAGCCATCTCAGCAAAACCCACAGGCCCAGGGCTCTGTCCAGCCTCAA 810

Gli-D2-14 CAGGGCTCCTTCCAGCCATCTCAGCAAAACCCACAGGCCCAGGGCTCTGTCCAGCCTCAA 807

Gli-D2-15 CAGGGCTCCTTCCAGCCATCTCAGCAAAACCCACAGGCCCAGGGCTCTGTCCAGCCTCAA 807

Gli-D2-18 CAGGGCTTCTTCCAACCATCTCAGCAAAACCCACAGGCCCAGGGCTCTGTCCAGCCTCAA 756

Gli-D2-16 CAGGGCTTCTTCCAACCATCTCAGCAAAACCCACAGGCCCAGGGCTCTTTCCAGCCTCAA 756

Gli-D2-17 CAGGGCTTCTTCCAACCATCTCAGCAAAACCCACAGGCCCAGGGCTCTTTCCAGCCTCAA 756

Gli-D2-19 CAGGGCTTCTTCCAACCATCTCAGCAAAATCCACAGGCCCAGGGCTCTTTCCAGCCTCAA 774

Gli-D2-3 CAGGGCTCCTTCCAGCCATCTCAGCAAAACCCACAGGCCCAGGGCTCTGTCCAGCCTCAA 741

Gli-D2-11 CAGGGCTCCTTCCAGCCATCTCAGCAAAACCCACAGGCCCAGGGCTCTGTCCAGCCTCAA 750

Gli-D2-9 CAGGGCTCCTTCCAGCCATCTCAGCAAAACCCACAGGCCCAGGGCTCTGTCCAGCCTCAA 762

Gli-D2-10 CAGGGCTCCTTCCAGCCATCTCAGCAAAACCCACAGGCCCAGGGCTCTGTCCAGCCTCAA 759

Gli-D2-8 CAGGGCTCCTTCCAGCCATCTCAGCAAAACCCACAGGCCCAGGGCTCTGTCCAGCCTCAA 753

Gli-D2-7 CAGGGCTCCTTCCAGCCATCTCAGCAAAACCCACAGGCCCAGGGCTCTGTCCAGCCTCAA 777

Gli-D2-6 CAGGGCTCCTTCCAGCCATCTCAGCAAAACCCACAGGCCCAGGGCTCTGTCCAGCCTCAA 753

Gli-D2-4 CAGGGCTCCTTCCAGCCATCTCAGCAAAACCCACAGGCCCAGGGCTCTGTCCAGCCTCAA 753

Gli-D2-5 CAGGGCTCCTTCCAGCCATCTCAGCAAAACCCACAGGCCCAGGGCTCTGTCCAGCCTCAA 750

Gli-D2-1 CAGGGCTCCTTCCAGTCATCTCAGCAAAACCCACAGGCCCAGGGCTCTGTCCAGCCTCAA 723

Gli-B2-3 CAGGGATCCTTCCAGCCATCTCAGCAGAACCCACAGGCCCAGGGCTCTGTCCAGCCTCAA 831

Gli-B2-9 CAGGGATCCTTCCAGCCATCTCAGCAGAACCCACAGGCCCAGGGCTCTGTCCAGCCTCAA 720

Gli-B2-7 CAGGGATCCTTCCAGCCATCTCAGCAGAACCCACAGGCCCAGGGCTCTGTCCAGCCTCAA 822

Gli-B2-10 CAGGGATCCTTCCAGCCATCTCAGCAGAACCCACAGGCCCAGGGCTCTGTCCAGCCTCAA 828

Gli-B2-4 CAGGGATCCTTCCAGCCATCTCAGCAGAACCCACAGGCCCAGGGCTCTGTCCAGCCTCAA 825

Gli-B2-6 CAGGGATCCTTCCAGCCATCTCAGCAGAACCCACAGGCCCAGGGCTCTGTCCAGCCTCAA 831

Gli-B2-5 CAGGGATCCTTCCAGCCATCTCAGCAGAACCCACAGGCCCAGGGCTCTGTCCAGCCTCAA 831

Gli-B2-8 CAGGGATCCTTCCAGCCATCTCAGCAGAACCCACAGGCCCAGGGCTCTGTCCAGCCTCAA 831

**** * ***** ********* ** *** **** * ******* **** ******

Gli-B2-12 CAACTTCCCCAGTTCGAGGAAATAAGGAATCTAGCGCTGCAGACGCTACCGGCAATGTGC 825

Gli-B2-2 CAACTTCCCCAGTTCGAGGAAATAAGGAATCTAGCGCTGCAGACGCTACCGGCAATGTGC 873

Gli-B2-11 CAACTGCCCCAGTTCGCGGAAATAAGGAACCTAGCGCTACAGACGCTACCTGCAATGTGC 894

Gli-B2-1 CAACTGCCCCAGTTTGAGGAAATAAGGAACCTAGCGCTAGAGACGCTACCTGCAATGTGC 804

Gli-A2-17 CAACTGCCCCAGTTCGAGGAAATAAGGAACCTAGCGCTACAGACGCTACCTGCAATGTGC 798

Gli-A2-15 CAACTGCCCCAGTTCGAGGAAATAAGGAACCTAGCGCTACAGACGCTACCTGCAATGTGC 801

Gli-A2-8 CAACTGCCCCAGTTCGAGGAAATAAGGAACCTAGCGCTACAGACGCTACCTGCAATGTGC 801

Gli-A2-4 CAACTGCCCCAGTTCGAGGAAATAAGGAACCTAGCGCTACAGACGCTACCTGCAATGTGC 801

Gli-A2-16 CAACTGCCCCAGTTCGAGGAAATAAGGAACCTAGCGCTACAGACGCTACCTGCAATGTGC 801

Gli-A2-11 CAACTGCCCCAGTTCGAGGAAATAAGGAACCTAGCGCTACAGACGCTACCTGCAATGTGC 792

Gli-A2-18 CAACTGCCCCAGTTCGAGGAAATAAGGAACCTAGCGCTACAGACGCTACCTGCAATGTGC 792

Gli-A2-6 CAACTGCCCCAGTTCGAGGAAATAAGGAACCTAGCGCTACAGACGCTACCTGCAATGTGC 822

Gli-A2-13 CAACTGCCCCAGTTCGAGGAAATAAGGAACCTAGCGCTACAGACGCTACCTGCAATGTGC 792

Gli-A2-14 CAACTGCCCCAGTTCGAGGAAATAAGGAACCTAGCGCTACAGACGCTACCTGCAATGTGC 792

Gli-A2-1 CAACTGCCCCAGTTCGAGGAAATAAGGAACCTAGCGCTACAGACGCTACCTGCAATGTGC 792

Gli-A2-2 CAACTGCCCCAGTTCGAGGAAATAAGGAACCTAGCGCTACAGACGCTACCTGCAATGTGC 792

Gli-A2-12 CAACTGCCCCAGTTCGAGGAAATAAGGAACCTAGCGCTACAGACGCTACCTGCAATGTGC 792

Gli-A2-3 CAACTGCCCCAGTTCGAGGAAATAAGGAACCTAGCGCTACAGACGCTACCTGCAATGTGC 825

Gli-A2-5 CAACTGCCCCAGTTCGAGGAAATAGTGAACCTAGCGCTACAGACGCTACCTGCAATGTGC 825

Gli-A2-7 CAACTGCCCCAGTTCGAGGAAATAAGGAACCTAGCGCTACAGACGCTACCTGCAATGTGC 825

Gli-A2-9 CAACTGCCCCAGTTCGAGGAAATAAGGAACCTAGCGCTACAGACGCTACCTGCAATGTGC 825

Gli-A2-10 CAACTGCCCCAGTTCGAGGAAATAAGGAACCTAGCGCTACAGACGCTACCTGCAATGTGC 825

Gli-D2-2 CAACTGCCCCAGTTTGAGGCAATAAGGAACCTAGCGCTACAGACGCTACCTGCAATGTGC 807

Gli-D2-12 CAGCTGCCCCAGTTTGAGGAAATAAGGAACCTAGCGCTAGAGACGCTACCTGCAATGTGC 864

Gli-D2-13 CAACTGCCCCAGTTTGAGGAAATAAGGAACCTAGCGCTAGAGACGCTACCTGCAATGTGC 870

Gli-D2-14 CAACTGCCCCAGTTTGAGGAAATAAGGAACCTAGCGCTAGAGACGCTACCTGCAATGTGC 867

Gli-D2-15 CAACTGCCCCAGTTTGAGGAAATAAGGAACCTAGCGCTAGAGACGCTACCTGCAATGTGC 867

Gli-D2-18 CAACTGCCCCAGTTTGAGGAAATAAGGAACCTAGCGCTAGAGACGCTACCTGCAATGTGC 816

Gli-D2-16 CAACTGCCCCAGTTTGAGGCAATAAGGAACCTAGCGCTACAGACGCTACCTGCAATGTGC 816

Gli-D2-17 CAACTGCCCCAGTTTGAGGCAATAAGGAACCTAGCGCTACAGACGCTACCTGCAATGTGC 816

Gli-D2-19 CAACTGCCCCAGTTTGAGGCAATAAGGAACCTAGCGCTACAGACGCTACCTGCAATGTGC 834

Gli-D2-3 CAACTGCCCCAGTTTGAGGAAATAAGGAACCTAGCGCTAGAGACGCTACCTGCAATGTGC 801

Gli-D2-11 CAACTGCCCCAGTTTGAGGAAATAAGGAACCTAGCGCTAGAGACGCTACCTGCAATGTGC 810

Gli-D2-9 CAACTGCCCCAGTTTGAGGAAATAAGGAACCTAGCGCTAGAGACGCTACCTGCAATGTGC 822

Gli-D2-10 CAACTGCCCCAGTTTGAGGAAATAAGGAACCTAGCGCTAGAGACGCTACCTGCAATGTGC 819

Gli-D2-8 CAACTGCCCCAGTTTGAGGAAATAAGGAACCTAGCGCTAGAGACGCTACCTGCAATGTGC 813

Gli-D2-7 CAACTGCCCCAGTTTGAGGAAATAAGGAACCTAGCGCTAGAGACGCTACCTGCAATGTGC 837

Gli-D2-6 CAACTGCCCCAGTTTGAGGAAATAAGGAACCTAGCGCTAGAGACGCTACCTGCAATGTGC 813

Gli-D2-4 CAACTGCCCCAGTTTGAGGAAATAAGGAACCTAGCGCTAGAGACGCTACCTGCAATGTGC 813

Gli-D2-5 CAACTGCCCCAGTTTGAGGAAATAAGGAACCTAGCGCTAGAGACGCTACCTGCAATGTGC 810

Gli-D2-1 CAACTGCCCCAGTTCCAGGAAATAAGGAACTTAGCGCTGCAGACGCTGCCAGCAATGTGC 783

Gli-B2-3 CAACTTCCCCAGTTCGAGGAAATAAGGAATCTAGCGCTGCAGACGCTACCGGCAATGTGC 891

Gli-B2-9 CAACTTCCCCAGTTCGAGGAAATAAGGAATCTAGCGCTGCAGACGCTACCGGCAATGTGC 780

Gli-B2-7 CAACTTCCCCAGTTCGAGGAAATAAGGAATCTAGCGCTGCAGACGCTACCGGCAATGTGC 882

Gli-B2-10 CAACTTCCCCAGTTCGAGGAAATAAGGAATCTAGCGCTGCAGACGCTACCGGCAATGTGC 888

Gli-B2-4 CAACTTCCCCAGTTCGAGGAAATAAGGAATCTAGCGCTGCAGACGCTACCGGCAATGTGC 885

Gli-B2-6 CAACTTCCCCAGTTCGAGGAAATAAGGAATCTAGCGCTGCAGACGCTACCGGCAATGTGC 891

Gli-B2-5 CAACTTCCCCAGTTCGAGGAAATAAGGAATCTAGCGCTGCAGACGCTACCGGCAATGTGC 891

Gli-B2-8 CAACTTCCCCAGTTCGAGGAAATAAGGAATCTAGCGCTGCAGACGCTACCGGCAATGTGC 891

** ** ******** ** **** *** ******* ******* ** *********

Gli-B2-12 AATGTCTACATCCCTCCATATTGCTCGACCACCATTGCGCCATCTGGCATCTTCGGTACC 885

Gli-B2-2 AATGTCTACATCCCTCCATATTGCTCGACCACCATTGCGCCATCTGGCATCTTCGGTACC 933

Gli-B2-11 AATGTCTACATCCCTCCACATTGCTCGACCACCATTGCGCCATTTGGCATCTTCGGTACC 954

Gli-B2-1 AATGTCTATATCCCTCCATATTGCACCA------TTGCTCCAGTTGGCATCTTCGGTACC 858

Gli-A2-17 AATGTCTACATCCCTCCATATTGCACCA------TCGCGCCATTTGGCATCTTCGGTACC 852

Gli-A2-15 AATGTCTACATCCCTCCATATTGCACCA------TCGCGCCATTTGGCATCTTTGGTACC 855

Gli-A2-8 AATGTCTACATCCCTCCATATTGCACCA------TCGCGCCATTTGGCATCTTCGGTACC 855

Gli-A2-4 AATGTCTACATCCCTCCATATTGCACCA------TCGCGCCATTTGGCATCTTCGGTACC 855

Gli-A2-16 AATGTCTACATCCCTCCATATTGCACCA------TCGCGCCATTTGGCATCTTCGGTACC 855

Gli-A2-11 AATGTCTACATCCCTCCATATTGCACCA------TCGCGCCATTTGGCATCTTCGGTACC 846

Gli-A2-18 AATGTCTACATCCCTCCATATTGCACCA------TCGCGCCATTTGGCATCTTCGGTACC 846

Gli-A2-6 AATGTCTACATCCCTCCATATTGCACCA------TCGCGCCATTTGGCATCTTCGGTACC 876

Gli-A2-13 AATGTTTACATCCCTCCATATTGCACCA------TGGCGCCATTTGGCATCTTCGGTACC 846

Gli-A2-14 AATGTCTACATCCCTCCATATTGCACCA------TCGCGCCATTTGGCATCTTCGGTACC 846

Gli-A2-1 AATGTCTACATCCCTCCATATTGCACCA------TCGCGCCATTTGGCATCTTCGGTACC 846

Gli-A2-2 AATGTTTACATCCCTCCATATTGCACCA------TGGCGCCATTTGGCATCTTCGGTACC 846

Gli-A2-12 AATGTCTACATCCCTCCATATTGCACCA------TCGCGCCATTTGGCATCTTCGGTACC 846

Gli-A2-3 AATGTCTACATCCCTCCATATTGCACCA------TCGCGCCATTTGGCATCTTCGGTACC 879

Gli-A2-5 AATGTCTACATCCCTCCATATTGCACCA------TCGCGCCATTTGGCATCTTCGGTACC 879

Gli-A2-7 AATGTCTACATCCCTCCATATTGCACCA------TCGCGCCATTTGGCATCTTCGGTACC 879

Gli-A2-9 AATGTCTACATCCCTCCATATTGCACCA------TCGCGCCATTTGGCATCTTCGGTACC 879

Gli-A2-10 AATGTCTACATCCCTCCATATTGCACCA------TCGCGCCATTTGGCATCTTCGGTACC 879

Gli-D2-2 AATGTGTATATCCCTCCATATTGCACCA------TTGCTCCATTTGGCATCTTCGGTACC 861

Gli-D2-12 AATGTCTATATCCCTCCATATTGCACCA------TTGCTCCAGTTGGCATCTTCGGTACC 918

Gli-D2-13 AATGTCTATATCCCTCCATATTGCACCA------TTGCTCCAGTTGGCATCTTCGGTACC 924

Gli-D2-14 AATGTCTATATCCCTCCATATTGCACCA------TTGCTCCAGTTGGCATCTTCGGTACC 921

Gli-D2-15 AATGTCTATATCCCTCCATATTGCACCA------TTGCTCCAGTTGGCATCTTCGGTACC 921

Gli-D2-18 AATGTCTATATCCCTCCATATTGCACCG------TTGCTCCAGTTGGCATCTTCGGTACC 870

Gli-D2-16 AATGTGTATATCCCTCCATATTGCACCA------TTGCTCCATTTGGCATCTTCGGTACC 870

Gli-D2-17 AATGTGTATATCCCTCCATATTGCACCA------TTGCTCCATTTGGCATCTTCGGTACC 870

Gli-D2-19 AATGTGTATATCCCTCCATATTGCACCA------TTGCTCCATTTGGCATCTTCGGTACC 888

Gli-D2-3 AATGTCTATATCCCTCCATATTGCACCA------TTGCTCAAGTTGGCATCTTCGGTACC 855

Gli-D2-11 AATGTCTATATCCCTCCATATTGCACCA------TTGCTCCAGTTGGCATCTTCGGTACC 864

Gli-D2-9 AATATCTATATCCCTCCATATTGCACCA------TTGCTCCAGTTGGCATCTTCGGTACC 876

Gli-D2-10 AATGTCTATATCCCTCCATATTGCACCA------TTGCTCCAGTTGGCATCTTCGGTACC 873

Gli-D2-8 AATGTCTATATCCCTCCATATTGCACCA------TTGCTCCAGTTGGCATCTTCGGTACC 867

Gli-D2-7 AATGTCTATATCCCTCCATATTGCACCA------TTGCTCCAGTTGGCATCTTCGGTACC 891

Gli-D2-6 AATGTCTATATCCCTCCATATTGCACCA------TTGCTCCAGTTGGCATCTTCGGTACC 867

Gli-D2-4 AATGTCTATATCCCTCCATATTGCACCA------TTGCTCCAGTTGGCATCTTCGGTACC 867

Gli-D2-5 AATGTCTATATCCCTCCATATTGCACCA------TTGCTCCAGTTGGCATCTTCGGTACC 864

Gli-D2-1 AATGTCTACATCCCTCCATATTGCTCGACCACCATTGCGCCATTTGGCATCTTCGGTACC 843

Gli-B2-3 AATGTCTACATCCCTCCATATTGCTCGACCACCATTGCGCCATCTGGCATCTTCGGTACC 951

Gli-B2-9 AATGTCTACATCCCTCCATATTGCTCGACCACCATTGCGCCATCTGGCATCTTCGGTACC 840

Gli-B2-7 AATGTCTACATCCCTCCATATTGCTCGACCACCATTGCGCCATCTGGCATCTTCGGTACC 942

Gli-B2-10 AATGTCTACATCCCTCCATATTGCTCGACCACCATTGCGCCATCTGGCATCTTCGGTACT 948

Gli-B2-4 AATGTCTACATCCCTCCATATTGCTCGACCACCATTGCGCCATCTGGCATCTTCGGTACC 945

Gli-B2-6 AATGTCTACATCCCTCCATATTGCTCGACCACCATTGCGCCATCTGGCATCTTCGGTACC 951

Gli-B2-5 AATGTCTACATCCCTCCATATTGCTCGACCACCATTGCGCCATCTGGCATCTTCGGTACC 951

Gli-B2-8 AATGTCTACATCCCTCCATATTGCTCGACCACCATTGCGCCATCTGGCATCTTCGGTACC 951

*** * ** ********* ***** * * ** * * ********* *****

Gli-B2-12 AACTGA 891

Gli-B2-2 AACTGA 939

Gli-B2-11 AACTGA 960

Gli-B2-1 AACTGA 864

Gli-A2-17 AACTGA 858

Gli-A2-15 AACTGA 861

Gli-A2-8 AACTGA 861

Gli-A2-4 AACTGA 861

Gli-A2-16 AACTGA 861

Gli-A2-11 AACTGA 852

Gli-A2-18 AACTGA 852

Gli-A2-6 AACTGA 882

Gli-A2-13 AACTGA 852

Gli-A2-14 AACTGA 852

Gli-A2-1 AACTGA 852

Gli-A2-2 AACTGA 852

Gli-A2-12 AACTGA 852

Gli-A2-3 AACTGA 885

Gli-A2-5 AACTGA 885

Gli-A2-7 AACTGA 885

Gli-A2-9 AACTGA 885

Gli-A2-10 AACTGA 885

Gli-D2-2 AACTGA 867

Gli-D2-12 AACTGA 924

Gli-D2-13 AACTGA 930

Gli-D2-14 AACTGA 927

Gli-D2-15 AACTGA 927

Gli-D2-18 AACTGA 876

Gli-D2-16 AACTGA 876

Gli-D2-17 AACTGA 876

Gli-D2-19 AACTGA 894

Gli-D2-3 AACTGA 861

Gli-D2-11 AACTGA 870

Gli-D2-9 AACTGA 882

Gli-D2-10 AACTGA 879

Gli-D2-8 AACTGA 873

Gli-D2-7 AACTGA 897

Gli-D2-6 AACTGA 873

Gli-D2-4 AACTGA 873

Gli-D2-5 AACTGA 870

Gli-D2-1 AACTGA 849

Gli-B2-3 AACTGA 957

Gli-B2-9 AACTGA 846

Gli-B2-7 AACTGA 948

Gli-B2-10 AACTGA 954

Gli-B2-4 AACTGA 951

Gli-B2-6 AACTGA 957

Gli-B2-5 AACTGA 957

Gli-B2-8 AACTGA 957

******
