# Supplementary material for: Production and molecular characterization of bread wheat lines with reduced amount of α-type gliadins
Source: BMC Plant Biol. 2017 Dec 19;17:248. doi: 10.1186/s12870-017-1211-3 (PMC5738072; doi:10.1186/s12870-017-1211-3)
Supplement: Supplementary file 2 — Amino acidic alignment of the signal peptide and the five domains corresponding to the 49 α-type gliadins isolated. Heptapeptide QPQLPYP in single copy or duplicated is highlighted in yellow; α-Glia (31–43) in gray; α-Glia (31–49) in gray + light blue; α-Glia (31–55) in gray + light blue + green; α-Glia (44–55) in light blue + green; α-Glia (51–70) underlined; α-Glia (56–75) in italic; α-Glia (206–217) in red; Glia-α in fuchsia; Glia-α2 in orange character; Glia-α9 in the black box; Glia-α20 in the blue box; the 33-mer peptide in the red box; the odd cysteine in bold and red character. (PDF 103 kb) [file 12870_2017_1211_MOESM2_ESM.pdf]

SIGNAL PEPTIDE

```
Gli-A2-7        MKTFLILALLAIVATTATTA   20
Gli-A2-9        MKTFLILALLAIVATTATTA   20
Gli-A2-10       MKTFLILALLAIVATTATTA   20
Gli-A2-6        MKTFLILALLAIVATTATTA   20
Gli-A2-5        MKTFLILALLAIVATTATTA   20
Gli-A2-8        MKTFLILALLAIVATTATTA   20
Gli-A2-11       MKTFLILALLAIVATTATTA   20
Gli-A2-14       MKTFLILALLAIVATTATTA   20
Gli-A2-13       MKTFLILALLAIVATTATTA   20
Gli-A2-12       MKTFLILALLAIVATTATTA   20
Gli-A2-15       MKTFLILALLAIVATTATTA   20
Gli-A2-16       MKTFLILALLAIVATTATTA   20
Gli-A2-18       MKTFLILALLAIVATTATTA   20
Gli-A2-17       MKTFLILALLAIVATTATTA   20
Gli-A2-3        MKTFLILVLLAIVATTATTA   20
Gli-A2-4        MKTFLILVLLAIVATTATTA   20
Gli-A2-2        MKTFLILVLLAIVATTATTA   20
Gli-A2-1        MKTFLILVLLAIVATTATTA   20
Gli-B2-1        MKTFLILALLAIVATTATTA   20
Gli-B2-2        MKTFLILALLAIVATTATTA   20
Gli-B2-3        MKTFLILALLAIVATTATTA   20
Gli-B2-12       MKTFLILALLAIVATTATTA   20
Gli-B2-11       MKTFLILALLAIVATTATTA   20
Gli-B2-6        MKTFLILSLLAIVATTATTA   20
Gli-B2-9        MKTFLILSLLAIVATTATTA   20
Gli-B2-5        MKTFLILSLLAIVATTATTA   20
Gli-B2-7        MKTFLILSLLAIVATTATTA   20
Gli-B2-10       MKTFLILSLLAIVATTATTA   20
Gli-B2-8        MKTFLILSLLAIVATTATTA   20
Gli-B2-4        MKTFLILSLLAIVATTATTA   20
Gli-D2-9        MKTFLILALLAIVATTATIA   20
Gli-D2-10       MKTFLILALLAIVATTATIA   20
Gli-D2-13       MKTFLILALLAIVATTATIA   20
Gli-D2-2        MKTFLILALLAIVATTATSA   20
Gli-D2-19       MKTFLILALLAIVATTATSA   20
Gli-D2-7        MKTFLILALLAIVATTATIA   20
Gli-D2-3        MKTFLILALLAIVATTATIA   20
Gli-D2-11       MKTFLILALLAIVATTATIA   20
Gli-D2-5        MKTFLILALLAIVATTATIA   20
Gli-D2-1        MKTFLILALLAIVATTATSA   20
Gli-D2-15       MKTFLILALLAIVATTATIA   20
Gli-D2-12       MKTFLILALLAIVATTATIA   20
Gli-D2-14       MKTFLILALLAIVATTATIA   20
Gli-D2-17       MKTFLILALRAIVATTATIA   20
Gli-D2-4        MKTFLILALLAIVATTATIA   20
Gli-D2-16       MKTFLILALLAIVATTATTA   20
Gli-D2-18       MKTFLILALLAIVATTATTA   20
Gli-D2-8        MKTFLILALLAIVATTATTA   20
Gli-D2-6        MKTFLILSLLAIVATTATTA   20
                ******* * ******** *
```

DOMAIN R1

```
Gli-A2-7      VRVPVPQLQPQNPSQQQPQEQVPLVQ-QQQFLGQQQPFPPQQPYPQPQPFPSQQPYLQLQPFPQP--------------QLPYSQPQPFRPQQPYPQPQPQYSQPQQPIS     95
Gli-A2-9      VRVPVPQLQPQNPSQQQPQEQVPLVQ-QQQFLGQQQPFPPQQPYPQPQPFPSQQPYLQLQPFPQP--------------QLSYSQPQPFRPQQLYPQPQPQYSQPQQPIS     95
Gli-A2-3      VRFPVPQLQPQNPSQQQPQEQVPLVQ-QQQFLGQQQPFPPQQPYPQPQPFPSQLPYLQLQPFPQP--------------QLPYSQPQPFRPQQPYPQPQPQYSQPQQPIS     95
Gli-A2-10     VRVPVPQLQPQNPSQQQPQEQVPLVQ-QQQFLGQQQPFPPQQPYPQPQPFPSQQPYLQLQPFPQP--------------QLSYSQPQPFRPQQLYPQPQPQYSQPQQPIS     95
Gli-A2-5      VRVPVPQLQPQNPSQQQPQEQVPLVQ-QQQFLGQQQPFPPQQPYPQPQPFPSQQPYLQLQPFPQP--------------QLSYSQPQPFRPQQLYPQPQPQYSQPQQPIS     95
Gli-A2-6      VRVPVPQLQPQNPSQQQPQEQVPLVQ-QQQFLGQQQPFPPQQPYPQPQPFPSQQPYLQLQPFPQP--------------QLSYSQPQPFRPQQPYPQPQPQYSQPQQPIS     95
Gli-A2-4      VRFPVPQLQPQNPSQQQPQEQVPLVQ-QQQFLGQQQPFPPQQPYPQPQPFPSQQPYLQLQPFPQP--------------QLPYSQPQPFRPQQPYPQPQPQYSQPQQPIS     95
Gli-A2-8      VRVPVPQLQPQNPSQQQPQEQVPLVQ-QQQFLGQQQPFPPQQPYPQPQPFPSQQPYLQLQPFPQP--------------QLPYSQPQPFRPQQPYPQPQPQYSQPQQPIS     95
Gli-A2-11     VRVPVPQLQPQNPSQQQPQEQVPLVQ-QQQFLGQQQPFPPQQPYPQPQPFPSQLPYLQLQPFPQP--------------QLPYSQPQPFRPQQPYPQPQPQYSQPQQPIS     95
Gli-A2-14     VRVPVPQLQPQNPSQQQPQEQVPLVQ-QQQFLGQQQPFPPQQPYPQPQPFPSQQPYLQLQPFPQP--------------QLPYSQPQPFRPQQPYPQPQPQYSQPQQPIS     95
Gli-A2-13     VRVPVPQLQPQNPSQQQPQEQVPLVQ-QQQFLGQQQPFPPQQPYPQPQPFPSQQPYLQLQPFPQP--------------QLSYSQPQPFRPQQPYPQPQPQYSQPQQPIS     95
Gli-A2-2      VRFPVPQLQPQNPSQQQPQEQVPLVQ-QQQFLGQQQPFPPQQPYPQPQPFPSQLPYLQLQPFPQP--------------QLPYSQPQPFRPQQPYPQPQPQYSQPQQPIS     95
Gli-A2-12     VRFPVPQLQPQNPSQQQPQEQVPLVQ-QQQFLGQQQPFPPQQPYPQPQPFPSQLPYLQLQPFPQP--------------QLPYSQPQPFRPQQPYPQPQPQYSQPQQPIS     95
Gli-A2-1      VRFPVPQLQPQNPSQQQPQEQVPLVQ-QQQFLGQQQPFPPQQPYPQPQPFPSQLPYLQLQPFPQP--------------QLPYSQPQPFRPQQPYPQPQPQYSQPQQPIS     95
Gli-A2-15     VRVPVPQLQPQNPSQQQPQEQVPLVQ-QQQFLGQQQPFPPQQPYPQPQPFPSQQPYLQLQPFPQP--------------QLSYSQPQPFRPQQPYPQPQPQYSQPQQPIS     95
Gli-A2-16     VRVPVPQLQPQNPSQQQPQEQVPLVQ-QQQFLGQQQPFPPQQPYPQPQPFPSQQPYLQLQPFPQP--------------QLPYSQPQPFRPQQPYPQPQPQYSQPQQPIS     95
Gli-A2-17     VRVPVPQLQPQNPSQQQPQEQVPLVQ-QQQFLGQQQPFPPQQPYPQPQPFPSQQPYLQLQPFPQP--------------QLPYSQPQPFRPQQPYPQPQPQYSQPQQPIS     95
Gli-A2-18     VRVPVPQLQPQNPSQQQPQEQVPLVQ-QQQFLGQQQPFPPQQPYPQPQPFPSQLPYLQLQPFPQP--------------QLPYSQPQPFRPQQPYPQPQPQYSQPQQPIS     95
Gli-B2-12     VRVPVPQPQPQNPSQPQPQGQVPLVQ-QQQFPGQQQQFPPQQPYPQPQPFPSQQPYLQLQPFPQPQPF---------PPQLPYPQPPPFSPQQQYPQPQPQYPQPQQPIS     100
Gli-B2-11     VRVPVPQLQPQNPSQQQPQEQVPLVQ-QQQFPGQQQQFPPQQPYPQPQPFPSQQPYLQLQPFPQPQPF---------PPQLPYPQPQSFPPQQPYPQQQPQYLQPQQPIS     100
Gli-B2-3      VTVPVPQLQPQNPSQQQPQKQVPLVQ-QQQFLGQQQPFPPQQPYPQPQPFPSQQPYP------QPQPF---------LPQLPYRPQPFLPQQPYPQPQPQYPQPQQPIS     94
Gli-B2-10     VRVPVPQLQPQNPSQQQPQEQVSLVQ-QLQYPGQQQPSPPQQPYPQPQPFPSQQPLPQPQPF-LP--------------QLPYPQPQPFFPPQQPYPQPQPQYPQPQQPIS     94
Gli-B2-8      VRVPVPQLQPQNPSQQQPQEQVSLVQ-QLQYPGQQQPFPPQQPYPQPQPFPSQQPLPQPQPF-LP--------------QLPYPQPQPFFPPQQPYPQPQPQYPQPQQPIS     94
Gli-B2-4      VRVPVPQLQPQNPSQQQPQEQVSLVQ-QLQYPGQQQPFPPQQPYPQPQPFPSQQPLPQPQPF-LP--------------QLPYPQPQPFFPPQQPYPQPQPQYPQPQQPIS     94
Gli-B2-7      VRVPVPQLQPQNPSQQQPQEQVSLVQ-QLQYPGQQQPFPPQQPYPQPQPFPSQQPLPQPQPF-LP--------------QLPYPQPQPFFPPQQPYPQPQPQYPQPQQPIS     94
Gli-B2-5      VRVPVPQLQPQNPSQQQPQEQVSLVQ-QLQYPGQQQPFPPQQPYPQPQPFPSQQPLPQPQPF-LP--------------QLPYPQPQPFFPPQQPYPQPQPQYPQPQQPIS     94
Gli-B2-6      VRVPVPQLQPQNPSQQQPQEQVSLVQ-QLQYPGQQQPFPPQQPYPQPQPFPSQQPLPQPQPF-LP--------------QLPYPQPQPFFPPQQPYPQPQPQYPQPQQPIS     94
Gli-B2-2      VRVPVPQLQPQNPSQQQPQEQVPLVQ-QQQFPGQQQQFPPQQPYPQPQPFPSQQPYLQLQPFPQPQPF---------PPQLPYPQPQSFPPQQPYPQQQPQYLQPQQPIS     100
Gli-B2-1      VRVPVPQLQPQNPSQQQPQEQVPLVQ-QQQFLGQQQPFPPQQPYPQPQPFPSQQPYLQLQPFPQP--------------QLPYSQPQPFRPQQPYPQPQPQYSQPQQPIS     95
Gli-B2-9      VRVPVPQLQPQNPSQQQPQEQVSLVQ-QLQYPGQQQPFPPQQPYPQPQP---------------------------------------------------QYPQPQQPIS     58
Gli-D2-7      VRVPVPQLQPQNPSQQQPQEQVPLVQ-QQQFPGQQQPFPPQQPYPQLQPFPSQQPYMQLQPFPQPQLPYP-------QPQLPYPQPQPFRPQQSYPQPQPQYSQPQQPIS     102
Gli-D2-19     VRVPVPQLQPQNPSQQQPQEQVPLVQ-QQQFPGQQQPFPPQQPYPQLQPFPSQQPYMQLQPFPQPQLPYP-------QPQLPYPQPQPFRPQQSYPQPQPQYSQPQQPIS     102
Gli-D2-3      VRVPVPQLQPQNPSQQQPQEQVPLVQ-QQQFPGQQQPFPPQQPYPQPQPFPSQQPYLQLQPFPQPQLPYP-------QPQLPYPQPQPFRPQQPYPQPQPQYSQPQQPIS     102
Gli-D2-14     VRVPVPQLQPQNPSQQQPQEQVPLVQ-QQQFPGQQQPFPPQQPYPQPQPFPSQQPYLQLQPFPQPQLPYPQPQLPYPQPQLPYPQPQPFRPQQPYPQSQPQYSQPQQPIS     109
Gli-D2-15     VRVPVPQLQPQNPSQQQPQEQVPLVQ-QQQFPGQQQPFPPQQPYPQPQPFPSQQPYLQLQPFPQPQLPYPQPQLPYPQPQLPYPQPQPFRPQQPYPQSQPQYSQPQQPIS     109
Gli-D2-12     VRVPVPQLQPQNPSQQQPQEQVPLVQ-QQQFPGQQQPFPPQQPYPQPQPFPSQQPYLQLQPFPQPQLPYPQPQLPYPQPQLPYPQPQPFRPQQPYPQSQPQYSQPQQPIS     109
Gli-D2-13     VRVPVPQLQPQNPSQQQPQEQVPLVQ-QQQFPGQQQPFPPQQPYPQPQPFPSQQPYLQLQPFPQPQLPYPQPQLPYPQPQLPYPQPQPFRPQQPYPQSQPQYSQPQQPIS     109
Gli-D2-17     VRVPVPQLQPQNPSQQQPQEQVPLVQ-EQQFPGQQQPFPPQQPYPQPQPFPSQQPYLQLQPFPQPQLPYP--------------QPQPFRPQQPYPQPQPQYSQPQQPIS     95
Gli-D2-6      VRVPVPQLQPQNPSQQQPQKQVPLVQ-QQQFPGQQQPFPPQQPYPQLQPFPSQQPYMQLQPFPQPQLPYP-------QPQLPYPQPQPFRPQQSYPQPQPQYSQPQQPIS     102
Gli-D2-4      VRVPVPQLQPQNPSQQQPQKQVPLVQ-QQQFPGQQQPFPPQQPYPQLQPFPSQQPYMQLQPFPQPQLPYP-------QPQLPYPQPQPFRPQQSYPQPQPQYSQPQQPIS     102
Gli-D2-5      VRVPVPQLQPQNPSQQQPQKQVPLVQ-QQQFPGQQQPFPPQQPYPQLQPFPSQQPYMQLQPFPQPQLPYP-------QPQLPYPQPQPFRPQQSYPQPQPQYSQPQQPIS     102
Gli-D2-10     VRVPVPQLQPQNPSQQQPQKQVPLVQ-QQQFPGQQQPFPPQQPYPQLQPFPSQQPYMQLQPFPQPQLPYP-------QPQLPYPQPQPFRPQQSYPQPQPQYSQPQQPIS     102
Gli-D2-9      VRVPVPQLQPQNPSQQQPQKQVPLVQ-QQQFPGQQQPFPPQQPYPQLQPFPSQQPYMQLQPFPQPQLPYP-------QPQLPYPQPQPFRPQQSYPQPQPQYSQPQQPIS     102
Gli-D2-16     VRVPVPQLQLQNPSQQQPQEQVPLVQ-EQQFPGQQQPFPPQQPYPQPQPFPSQQPYLQLQPFPQPQLPYP--------------QPQPFRPQQPYPQPQPQYSQPQQPIS     95
Gli-D2-18     VRVPVPQLQLQNPSQQQPQEQVPLVQ-EQQFPGQQQPFPPQQPYPQPQPFPSQQPYLQLQPFPQPQLPYP--------------QPQPFRPQQPYPQPQPQYSQPQQPIS     95
Gli-D2-2      VRVPVPQLQPQNPSQQQPQEQVPLMQQQQQFPGQQEQFPPQQPYPHQQPFPSQQPYPQPQPFP---------------PQLPYPQTQPFPPQQPYPQPQPQYPQPQQPIS     95
Gli-D2-11     VRVPVPQLQPQNPSQQQPQEQVPLVQ-QQQFPGQQQPFPPQQPYPQPQPFPSQQPYLQLQPFPQPQLPYP-------QPHLPYPQPQPFRPQQPYPQPQPQYSQPQQPIS     102
Gli-D2-1      VRVPVPQLQPQNPSQQQPQEQVPLMQQQQQFPGQQEQFPPQQPYPHQQPFPSQQPYPQPQPF---------------PPQLPYPQTQPFPPQQPYPQPQPQYPQPQQPIS     95
Gli-D2-8      VRVPVPQLQPQNPSQQQPQEQVPLVQ-QQQFPGQQQQFPPQQPYPQPQPFPSQQPYMQLQPFPQPQLPYP-------QPQLPYPQPQPFRPQQSYPQPQPQYSQPQQPIS     102
              *  .**** * ***** *** ** *:* : *: ***:  *******: **                                                ** *******
```

DOMAIN QR1

```
Gli-A2-8       -------QQQQQQ-QQ-QQ-QQ-Q-QQEQQ   18
Gli-A2-4       -------QQQQQQ-QQ-QQ-QQ-Q-QQEQQ   18
Gli-A2-17      --------QQQQQ-QQ-QQ-QQ-Q-QQEQQ   17
Gli-A2-6       -----QQQQQQQQQQQQQQQQQQQQQQEQQ   25
Gli-A2-18      -------QQQQQQ-QQ-QQ-QQ-Q-Q---Q   15
Gli-A2-16      -------QQQQQQ-QQ-QQ-QQ-Q-Q---Q   15
Gli-A2-15      -------QQQQQQ-QQ-QQ-QQ-Q-Q---Q   15
Gli-A2-1       -------QQQQQQ-QQ-QQ-QQ-Q-Q---Q   15
Gli-A2-12      -------QQQQQQ-QQ-QQ-QQ-Q-Q---Q   15
Gli-A2-2       -------QQQQQQ-QQ-QQ-QQ-Q-Q---Q   15
Gli-A2-13      -------QQQQQQ-QQ-QQ-QQ-Q-Q---Q   15
Gli-A2-14      -------QQQQQQ-QQ-QQ-QQ-Q-Q---Q   15
Gli-A2-5       ----QQQQQQQQQQQQQQQQQQQQQQQEQQ   26
Gli-A2-10      ----QQQQQQQQQQQQQQQQQQQQQQQEQQ   26
Gli-A2-3       ----QQQQQQQQQQQQQQQQQQQQQQQEQQ   26
Gli-A2-9       ----QQQQQQQQQQQQQQQQQQQQQQQEQQ   26
Gli-A2-7       ----QQQQQQQQQQQQQQQQQQQQQQQEQQ   26
Gli-A2-11      -------QQQQQQ-QQ-QQ-QQ-Q-Q---Q   15
Gli-B2-1       -------QQQQQQ-QQ-QQ-QQ-Q-QQEQQ   18
Gli-B2-2       QQQAQ---QQQQQQQQQ----QQ-QQ---Q   19
Gli-B2-3       QQQAQQAQQQQQQQQQQQQQQQQQQQQQQQ   30
Gli-B2-8       QQQAQQAQQQQQQRQQQQQQQQQQQQQQQQ   30
Gli-B2-4       QQQAQQAQQQQQQQQQQQQQQQQQQQQQQQ   30
Gli-B2-10      QQQAQQAQQQQQQQQQQQQQQQQQQQQQQQ   30
Gli-B2-7       QQQAQQAQQQQQQQQQQQQQQQQQQQQ--Q   28
Gli-B2-5       QQQAQQAQQQQQQQQQQQQQQQQQQQQQQQ   30
Gli-B2-6       QQQAQQAQQQQQRQQQQQQQQQQQQQQQQQ   30
Gli-B2-12      QQQAQ---QQQQQQQQQ----QQ-QQQQQQ   22
Gli-B2-9       QQQAQQAQQQQQQQQQQQQQQQQQQQQQQ-   29
Gli-B2-11      QQQAQ---QQQQQQQQQ----QQ-QQ---Q   19
Gli-D2-15      -------QQQQQQ-QQQQQKQQQQ-QQQQQ   21
Gli-D2-8       -----QQ-Q-Q-Q-Q-QQ-Q-Q-QQQQ---   14
Gli-D2-6       -------QQQQQ-Q-Q-Q-Q-Q-Q--Q-QQ   14
Gli-D2-4       -------QQQ---Q-QQQ-QQQQQ--Q-Q-   14
Gli-D2-14      --------QQQQQQQQQQQKQQRQQQQQQ-   21
Gli-D2-17      -------QQQQ--Q-QQ--QQQQQ--Q-QQ   15
Gli-D2-16      -------QQQQ--Q-QQQ-QQQ-Q--Q-QQ   15
Gli-D2-18      -------QQQQ--Q-QQQ-QQQ-Q--Q-QQ   15
Gli-D2-12      -------QQQQQQQQQQQQKQQQQQQQQ--   21
Gli-D2-1       QQQAQ---QQQQQ------------Q---Q   12
Gli-D2-5       -------QQQQ-Q-Q-Q-Q-Q-Q-Q--QQ-   13
Gli-D2-2       QQQAQ---QQQQQ------------Q---Q   12
Gli-D2-7       -------QQQ---Q-QQQ-QQQ-Q----QQ   13
Gli-D2-19      -------QQQ---Q-QQQ-QQQQQ----QQ   14
Gli-D2-13      --------QQQQQQQQQQQKQQQQQQQQQQ   22
Gli-D2-10      -------QQQQ----QQQ-QQQQQ----QQ   14
Gli-D2-9       -------QQQQQ---Q---QQQQ-QQ--QQ   14
Gli-D2-3       -------QQ-Q---Q--Q--Q--Q-QQ---   9
Gli-D2-11      -------QQQ--Q--Q--Q--Q-Q-QQ---   10
                       *
```

DOMAIN NR1

```
Gli-A2-11       -ILQQILQQQLIPCMDVVLQQHNIAHGRSQVLQQSTYQLLQGLCCQHLWQIPEQSQCQAIHNVVHAIILH-------    69
Gli-A2-5        -IRQQILQQQLIPCMDVVLQQHNIAHGRSQVLQQSTYQLLQELCCQHLWQIPEQSQCQAIHNVVHAIILH-------    69
Gli-A2-7        -ILQQILQQQLIPCMDVVLQQHNIAHGRSQVLQQSTYQLLQELCCQHLWQIPEQSQCQAIHNVVHAIILH-------    69
Gli-A2-9        -ILQQILQQQLIPCMDVVLQQHNIAHGRSQVLQQSTYQLLQELCCQHLWQIPEQSQCQAIHNVVHAIILH-------    69
Gli-A2-3        -ILQQILQQQLIPCMDVVLQQHNIAHGRSQVLQQSTYQLLQELCCQHLWQIPEQSQCQAIHNVVHAIILH-------    69
Gli-A2-10       -ILQQILQQQLIPCMDVVLQQHNIAHGRSQVLQQSTYQLLQELCCQHLWQIPEQSQCQAIHNVVHAIILH-------    69
Gli-A2-6        -ILQQILQQQLIPCMDVVLQQHNIAHGRSQVLQQSTYQLLQELCCQHLWQIPEQSQCQAIHNVVHAIILH-------    69
Gli-A2-4        -ILQQILQQQLIPCMDVVLQQHNIAHGRSQVLQQSTYQLLQELCCQHLWQIPEQSQCQAIHNVVHAIILH-------    69
Gli-A2-8        -----ILQQQLIPCMDVVLQQHNIAHGRSQVLQQSTYQLLQELCCQHLWQIPEQSQCQAIHNVVHAIILH-------    69
Gli-A2-14       -----ILQQQLIPCMDVVLQQHNIAHGRSQVLQQSTYQLLQELCCQHLWQIPEQSQCQAIHNVVHAIILH-------    69
Gli-A2-13       -ILQQILQQQLIPCMDVVLQQHNIAHGRSQVLQQSTYQLLQELCCQHLWQIPEQSQCQAIHNVVHAIILH-------    69
Gli-A2-2        -ILQQILQQQLIPCMDVVLQQHNIAHGRSQVLQQSTYQLLQELCCQHLWQIPEQSQCQAIHNVVHAIILH-------    69
Gli-A2-12       -ILQQILQQQLIPCMDVVLQQHNIAHGRSQVLQQSTYQLLQELCCQHLWQIPEQSQCQAIHNVVHAIILH-------    69
Gli-A2-1        -ILQQILQQQLIPCMDVVLQQHNIAHGRSQVLQQSTYQLLQELCCQHLWQIPEQSQCQAIHNVVHAIILH-------    69
Gli-A2-15       -ILQQILQQQLIPCMDVVLQQHNIAHGRSQVLQQSTYQLLQELCCQHLWQIPEQSQCQAIHNVVHAIILH-------    69
Gli-A2-16       -ILQQILQQQLIPCMDVVLQQHNIAHGRSQVLQQSTYQLLQELCCQHLWQIPEQSQCQAIHNVVHAIILH-------    69
Gli-A2-17       -ILQQILQQQLIPCMDVVLQQHNIAHGRSQVLQQSTYQLLQELCCQHLWQIPEQSQCQAIHNVVHAIILH-------    69
Gli-A2-18       -ILQQILQQQLIPCMDVVLQQHNIAHGRSQVLQQSTYQLLQELCCQHLWQIPEQSQCQAIHNVVHAIILH-------    69
Gli-B2-3        ILQQILQQQQLIPCRDVVLQQHNIAHASSQVLQQSSYQLLQQLCCQRLWQIPEQSRCQAIHNVVHAITL--------    69
Gli-B2-8        ILQQILQQQLLIPCRDVVLQQHNIAHASSQVLQQSSYQLLQQLCCQRLWQIPEQSRCQAIHNVVHAITL--------    69
Gli-B2-9        ILQQILQQQQLIPCRDVVLQQHNIAHASSQVLQQSSYQLLQQLCCQRLWQIPERSRCQAIHNVVHAITL--------    69
Gli-B2-6        ILQQILQQQQLIPCRDVVLQQHNIAHASSQVLQQSSYQLLQQLCCQRLWQIPEQSRCQAIHNVVHAITL--------    69
Gli-B2-5        ILQQILQQQQLIPCRDVVLQQHNIAHASSQVLQQSSYQLLQQLCCQRLWQIPEQSRCQAIHNVVHAITL--------    69
Gli-B2-7        ILQQILQQQQLIPCRDVVLQQHNIAHASSQVLQQSSYQLLQQLCCQRLWQIPEQSRCQAIHNVVHAITL--------    69
Gli-B2-10       ILQQILQQQQLIPCRDVVLQQHNIAHASSQVLQQSSYQLLQQLCCQRLWQIPEQSRCQAIHNVVHAITL--------    69
Gli-B2-4        ILQQILQQQQLIPCRDVVLQQHNIAHASSQVLQQSSYQLLQQLCCQRLWQIPEQSRCQAIHNVVHAITL--------    69
Gli-B2-1        ILQQI-LQQQLIPCMDVVLQQHNIAHGTSQVLQQSTYQLLQELCCQHLWQIPEQSQCQAIHNVVHAIILH-------    69
Gli-B2-12       ILQQI-LQQQLIPCRDVVLQQHNIAHARSQVLQQSTYQPLQQLCCQQLWQIPEQSRCQAIHNVVHAIILH-------    69
Gli-B2-2        ILQQI-LQQQLIPCRDVVLQQHYIAHASSQVLQQSTYQLLQQLCCQQLLQIPEQSRCQAIHNVAHAIIM--------    68
Gli-B2-11       ILQQI-LQQQLIPCRDVVLQQHNIAHASSQVLQQSTYQLLQQLCCQQLLQIPEQSRCQAIHNVAHAIIMH-------    69
Gli-D2-1        -ILQQILQQQLIPCRDVVLQQHNIAHASSQVLQQSSYQQLQQLCCQQLFQIPEQSRCQAIHNVVHAIILHHH-----    71
Gli-D2-19       -----ILQQQLIPCRDVVLQQHSIAHGSSQVLQQSTYQLVQQLCCQQLWQIPEQSRCQAIHNVVHAIILHQQHHHHR    72
Gli-D2-2        -ILQQILQQQLIPCRDVVLQQHNIAHASSQVLQQSTYQLVQQLCCQQLWQIPEQSRCQAIHNVVHAIILHQQHHHH-    75
Gli-D2-18       -ILQQILQQQLIPCRDVVLQQHNIAHGSSQVLQESTYQLVQQLCCQQLWQIPEQSRCQAIHNVVHAIILHQQHHHH-    75
Gli-D2-16       -ILQQILQQQLIPCRDVVLQQHNIAHGSSQVLQESTYQLVQQLCCQQLWQIPEQSRCQAIHNVVHAIILHQQHHHH-    75
Gli-D2-17       -ILQQILQQQLIPCRDVVLQQHNIAHGSSQVLQESTYQLVQQLCCQQLWQIPEQSRCQAIHNVVHAIILHHH-----    71
Gli-D2-3        -ILQQILQQQLIPCRDVVLQQHNIAHGRSQVLQQSTYQLVQQLCCQQLWQIPEQSRCQAIHNVVHAIILH-------    69
Gli-D2-9        -ILQQILQQQLIPCRDVVLQQHSIAHGSSQVLQQSTYQLVQQLCCQQLWQIPEQSRCQAIHNVVHAIILH-------    69
Gli-D2-7        -ILQQILQQQLIPCRDVVLQQHSIAHGSSQVLQQSTYQLVQQLCCQQLWQIPEQSRCQAIHNVVHAIILH-------    69
Gli-D2-11       -ILQQILQQQLIPCRDVVLQQHSIAHGSSQVLQQSTYQLVQQLCCQQLWQIPEQSRCQAIHNVVHAIILH-------    69
Gli-D2-5        -ILQQILQQQLIPCRDVVLQQHSIAHGSSQVLQQSTYQLVQQLCCQQLWQIPEQSRCQAIHNVVHAIILH-------    69
Gli-D2-4        -ILQQILQQQLIPCRDVVLQQHSIAHGSSQVLQQSTYQLVQQLCCQQLWQIPEQSRCQAIHNVVHAIILH-------    69
Gli-D2-6        -ILQQILQQQLIPCRDVVLQQHSIAHGSSQVLQQSTYQLVQQLCCQQLWQIPEQSRCQAIHNVVHAIILH-------    69
Gli-D2-8        -ILQQILQQQLIPCRDVVLQQHSIAHGSSQVLQQSTYQLVQQLCCQQLWQIPEQSRCQAIHNVVHAIILH-------    69
Gli-D2-10       -ILQQILQQQLIPCRDVVLQQHSIAYGSSQVLQQSTYQLVQQLCCQQLWQIPEQSRCQAIHNVVHAIILH-------    69
Gli-D2-13       -ILQQILQQQLIPCRDVVLQQHSIAYGSSQVLQQSTYQLVQQLCCQQLWQIPEQSRCQAIHNVVHAIILH-------    69
Gli-D2-12       -ILQQILQQQLIPCRDVVLQQHSIAYGSSQVLQQSTYQLVQQLCCQQLWQIPEQSRCQAIHNVVHAIILH-------    69
Gli-D2-15       -ILQQILQQQLIPCRDVVLQQHSIAYGSSQVLQQSTYQLVQQLCCQQLWQIPEQSRCQAIHNVVHAIILH-------    69
Gli-D2-14       -ILQQILQQQLIPCRDVVLQQHSIAYGSSQVLQQSTYQLVQQLCCQQLWQIPEQSRCQAIHNVVHAIILH-------    69
                       ** **** ******* **:. *****:*:** :* ****:* ****:*:*******.*** :
```

DOMAIN QR2

```
Gli-A2-7        -----------------------QQQKQQQQ--         8
Gli-A2-9        -----------------------QQQKQQQQ--         8
Gli-A2-3        -----------------------QQQKQQQQ--         8
Gli-A2-10       -----------------------QQQKQQQQ--         8
Gli-A2-5        -----------------------QQQKQQQQ--         8
Gli-A2-6        -----------------------QQQKQQQQ--         8
Gli-A2-4        -----------------------QQQKQQQQ--         8
Gli-A2-8        -----------------------QQQKQQQQ--         8
Gli-A2-11       -----------------------QQQKQQQQ--         8
Gli-A2-14       -----------------------QQQKQQQQ--         8
Gli-A2-13       -----------------------QQQKQQQQ--         8
Gli-A2-2        -----------------------QQQKQQQQ--         8
Gli-A2-12       -----------------------QQQKQQQQ--         8
Gli-A2-1        -----------------------QQQKQQQQ--         8
Gli-A2-15       -----------------------QQQKQQQQ--         8
Gli-A2-16       -----------------------QQQKQQQQ--         8
Gli-A2-17       -----------------------QQQKQQQQ--         8
Gli-A2-18       -----------------------QQQKQQQQ--         8
Gli-B2-11       QQQQQQQEQKQQLQQQQQQQQQLQQQQQQQQQQ         33
Gli-B2-4        QQQQQQQQQ--------QQQQQQQQQQQQQQQQ         25
Gli-B2-3        QQQQQQQQQQQ------QQQQQQQQQQQQQQQQ         27
Gli-B2-8        QQQQQQQQQQQ------QQQQQQQQQQQQQQQQ         27
Gli-B2-10       QQQQQQQQQQQ------QQQQQ-QQQQQQQQQQ         26
Gli-B2-7        QQQQQQQQQQQ---Q--QQQQ--QQQQQQQQQQ         26
Gli-B2-5        QQQQQQQQQQQ---QQ-QQQQ--QQQQQQQQQQ         27
Gli-B2-6        QQQQQQQQQQQ---QQ-QQQQ--QQQQQQQQQQ         27
Gli-B2-9        QQQQQQQQQQQ---QQ-QQQQ--QQQQQQQQQQ         27
Gli-B2-1        -------------QQQQQQQ-----------QQ         9
Gli-B2-2        QQQQQQQQQQQ---QQ-QQQQ--QQQQQQQQQQ         27
Gli-B2-12       ---------------QQQRQ----Q---Q----         7
Gli-D2-17       QQQ-----------------------QQQQQQQ         10
Gli-D2-16       -------------QQ----------QQQQQQQQ         10
Gli-D2-18       -------------QQ----------QQQQQQQQ         10
Gli-D2-14       -------------QQQQQQ------QQ-QQQKQ         13
Gli-D2-15       -------------QQ--QQQQ-----QQQQQKQ         13
Gli-D2-12       -------------QQQQQQQ------QQQQ--Q         12
Gli-D2-11       QQ-------------QQQRQ----Q---QQ-QQ         12
Gli-D2-7        QQ-------------QQQQQQQQQ--QQQQQQQ         18
Gli-D2-2        --------------QQQQ---------QQQQQQ         10
Gli-D2-13       -------------QQQQQQQ-------QQQQKQ         13
Gli-D2-10       QQ---------------QQQQQ-----QQQ--Q         11
Gli-D2-9        QQ-------------Q-QQQQ------QQQQ-Q         12
Gli-D2-19       -------------QQQQQQQ-----------QQ         9
Gli-D2-3        -------------QQQQ-QQ----------Q-Q         8
Gli-D2-5        -------------QQQQQQQ-----------QQ         9
Gli-D2-1        -------------QQQ---QQ-----------Q         6
Gli-D2-4        -------------QQQQQQQ-----------QQ         9
Gli-D2-6        -------------QQQQQQQ-----------QQ         9
Gli-D2-8        -------------QQQQQQQ-----------QQ         9
```

DOMAIN NR2

```
Gli-A2-5        PSSQFSFQQPLQQYPLGQGSFRPSQQNPEAQGSVQPQQLPQFEEIVNLALQTLPAMCNVYIPPYCT--IAPFGIFGTN  76
Gli-A2-4        PSSQFSFQQPLQQYPLGQGSFRPSQQNPQAQGSVQPQQLPQFEEIRNLALQTLPAMCNVYIPPYCT--IAPFGIFGTN  76
Gli-A2-18       PSSQFSFQQPLQQYPLGQGSFRPSQQNPQAQGSVQPQQLPQFEEIRNLALQTLPAMCNVYIPPYCT--IAPFGIFGTN  76
Gli-A2-17       PSSQFSFQQPLQQYPLGQGSFRPSQQNPQAQGSVQPQQLPQFEEIRNLALQTLPAMCNVYIPPYCT--IAPFGIFGTN  76
Gli-A2-16       PSSQFSFQQPLQQYPLGQGSFRPSQQNPQAQGSVQPQQLPQFEEIRNLALQTLPAMCNVYIPPYCT--IAPFGIFGTN  76
Gli-A2-15       PSSQFSFQQPLQQYPLGQGSFRPSQQNPQAQGSVQPQQLPQFEEIRNLALQTLPAMCNVYIPPYCT--IAPFGIFGTN  76
Gli-A2-11       PSSQFSFQQPLQQYPLGQGSFRPSQQNPQAQGSVQPQQLPQFEEIRNLALQTLPAMCNVYIPPYCT--IAPFGIFGTN  76
Gli-A2-13       PSSQVSFQQPLQQYPLGQGSFRPSQQNPQAQGSVQPQQLPQFEEIRNLALQTLPAMCNVYIPPYCT--MAPFGIFGTN  76
Gli-A2-2        PSSQVSFQQPLQQYPLGQGSFRPSQQNPQAQGSVQPQQLPQFEEIRNLALQTLPAMCNVYIPPYCT--MAPFGIFGTN  76
Gli-A2-7        PSSQVSFQQPLQQYPLGQGSFRPSQQNPQAQGSVQPQQLPQFEEIRNLALQTLPAMCNVYIPPYCT--IAPFGIFGTN  76
Gli-A2-9        PSSQVSFQQPLQQYPLGQGSFRPSQQNPQAQGSVQPQQLPQFEEIRNLALQTLPAMCNVYIPPYCT--IAPFGIFGTN  76
Gli-A2-3        PSSQVSFQQPLQQYPLGQGSFRPSQQNPQAQGSVQPQQLPQFEEIRNLALQTLPAMCNVYIPPYCT--IAPFGIFGTN  76
Gli-A2-10       PSSQVSFQQPLQQYPLGQGSFRPSQQNPQARGSVQPQQLPQFEEIRNLALQTLPAMCNVYIPPYCT--IAPFGIFGTN  76
Gli-A2-6        PSSQVSFQQPLQQYPLGQGSFRPSQQNPQAQGSVQPQQLPQFEEIRNLALQTLPAMCNVYIPPYCT--IAPFGIFGTN  76
Gli-A2-8        PSSQVSFQQPLQQYPLGQGSFRPSQQNPQAQGSVQPQQLPQFEEIRNLALQTLPAMCNVYIPPYCT--IAPFGIFGTN  76
Gli-A2-14       PSSQVSFQQPLQQYPLGQGSFRPSQQNPQAQGSVQPQQLPQFEEIRNLALQTLPAMCNVYIPPYCT--IAPFGIFGTN  76
Gli-A2-12       PSSQVSFQQPLQQYPLGQGSFRPSQQNPQAQGSVQPQQLPQFEEIRNLALQTLPAMCNVYIPPYCT--IAPFGIFGTN  76
Gli-A2-1        PSSQVSFQQPLQQYPLGQGSFRPSQQNPQAQGSVQPQQLPQFEEIRNLALQTLPAMCNVYIPPYCT--IAPFGIFGTN  76
Gli-B2-11       PSSQVSFQQPQQQYPSSQVSFQPSQLNPQAQGSVQPQQLPQFAEIRNLALQTLPAMCNVYIPPHCSTTIAPFGIFGTN  78
Gli-B2-12       PSSQVSLQQPQQQYPSGQGSFQPSQQNPQAQGSVQPQQLPQFEEIRNLALQTLPAMCNVYIPPYCSTTIAPSGIFGTN  78
Gli-B2-3        PSSQVSYQQPQQQYPSGQGSFQPSQQNPQAQGSVQPQQLPQFEEIRNLALQTLPAMCNVYIPPYCSTTIAPSGIFGTN  78
Gli-B2-8        PSSQVSYQQPQQQYPSGQGSFQPSQQNPQAQGSVQPQQLPQFEEIRNLALQTLPAMCNVYIPPYCSTTIAPSGIFGTN  78
Gli-B2-4        PSSQVSYQQPQQQYPSGQGSFQPSQQNPQAQGSVQPQQLPQFEEIRNLALQTLPAMCNVYIPPYCSTTIAPSGIFGTN  78
Gli-B2-10       PSSQVSYQQPQQQYPSGQGSFQPSQQNPQAQGSVQPQQLPQFEEIRNLALQTLPAMCNVYIPPYCSTTIAPSGIFGTN  78
Gli-B2-7        PSSQVSYQQPQQQYPSGQGSFQPSQQNPQAQGSVQPQQLPQFEEIRNLALQTLPAMCNVYIPPYCSTTIAPSGIFGTN  78
Gli-B2-5        PSSQVSYQQPQQQYPSGQGSFQPSQQNPQAQGSVQPQQLPQFEEIRNLALQTLPAMCNVYIPPYCSTTIAPSGIFGTN  78
Gli-B2-6        PSSQVSYQQPQQQYPSGQGSFQPSQQNPQAQGSVQPQQLPQFEEIRNLALQTLPAMCNVYIPPYCSTTIAPSGIFGTN  78
Gli-B2-9        PSSQVSYQQPQQQYPSGQGSFQPSQQNPQAQGSVQPQQLPQFEEIRNLALQTLPAMCNVYIPPYCSTTIAPSGIFGTN  78
Gli-B2-1        PLSQVCFQQSQQQYPSGQGSFQPSQQNPQAQGSVQPQQLPQFEEIRNLALETLPAMCNVYIPPYCSTTIAPSGIFGTN  78
Gli-B2-2        PSSQVSYQQPQQQYPSGQGSFQPSQQNPQAQGSVQPQQLPQFEEIRNLALQTLPAMCNVYIPPYCSTTIAPSGIFGTN  78
Gli-D2-18       PLSQVSFQQPQQQYPSGQGFFQPSQQNPQAQGSVQPQQLPQFEEIRNLALETLPAMCNVYIPPYCT--VAPVGIFGTN  76
Gli-D2-17       PLSQVSFQQPQQQYPSGQGFFQPSQQNPQAQGSFQPQQLPQFEAIRNLALQTLPAMCNVYIPPYCT--IAPFGIFGTN  76
Gli-D2-16       PLSQVSFQQPQQQYPSGQGFFQPSQQNPQAQGSFQPQQLPQFEAIRNLALQTLPAMCNVYIPPYCT--IAPFGIFGTN  76
Gli-D2-19       PLSQVSFQQPQQQYPSGQGFFQPSQQNPQAQGSFQPQQLPQFEAIRNLALQTLPAMCNVYIPPYCT--IAPFGIFGTN  76
Gli-D2-2        PLSQVSFQQPQQQYPSGQGFFQPSQQNPQAQGSFQPQQLPQFEAIRNLALQTLPAMCNVYIPPYCT--IAPFGIFGTN  76
Gli-D2-9        PLSQVSFQQPQQQYPSGQGSFQPSQQNPQAQGSVQPQQLPQFEEIRNLALETLPAMCNIYIPPYCT--IAPFGIFGTN  76
Gli-D2-1        PSSQVSYQQPQEQYPSGQGSFQSSQQNPQAQGSVQPQQLPQFQEIRNLALQTLPAMCNVYIPPYCSTTIAPFGIFGTN  78
Gli-D2-14       PLSQVSFQQPQQQYPSGQGSFQPSQQNPQAQGSVQPQQLPQFEEIRNLALETLPAMCNVYIPPYCT--IAPVGIFGTN  76
Gli-D2-15       PLSQVSFQQPQQQYPSGQGSFQPSQQNPQAQGSVQPQQLPQFEEIRNLALETLPAMCNVYIPPYCT--IAPVGIFGTN  76
Gli-D2-12       PLSQVSFQQPQQQYPSGQGSFQPSQQNPQAQGSVQPQQLPQFEEIRNLALETLPAMCNVYIPPYCT--IAPVGIFGTN  76
Gli-D2-11       PLSQVSFQQPQQQYPSGQGSFQPSQQNPQAQGSVQPQQLPQFEEIRNLALETLPAMCNVYIPPYCT--IAPVGIFGTN  76
Gli-D2-3        PLTQVSFQQPQQQYPSGQGSFQPSQQNPQAQGSVQPQQLPQFEEIRNLALETLPAMCNVYIPPYCT--IAPVGIFGTN  76
Gli-D2-13       PLSQVSFQQPQQQYPSGQGSFQPSQQNPQAQGSVQPQQLPQFEEIRNLALETLPAMCNVYIPPYCT--IAPVGIFGTN  76
Gli-D2-10       PLSQVSFQQPQQQYPSGQGSFQPSQQNPQAQGSVQPQQLPQFEEIRNLALETLPAMCNVYIPPYCT--IAPVGIFGTN  76
Gli-D2-7        PLSQVCFQQSQQQYPSGQGSFQPSQQNPQAQGSVQPQQLPQFEEIRNLALETLPAMCNVYIPPYCT--IAPVGIFGTN  76
Gli-D2-5        PLSQVCFQQSQQQYPSGQGSFQPSQQNPQAQGSVQPQQLPQFEEIRNLALETLPAMCNVYIPPYCT--IAPVGIFGTN  76
Gli-D2-4        PLSQVCFQQSQQQYPSGQGSFQPSQQNPQAQGSVQPQQLPQFEEIRNLALETLPAMCNVYIPPYCT--IAPVGIFGTN  76
Gli-D2-6        PLSQVCFQQSQQQYPSGQGSFQPSQQNPQAQGSVQPQQLPQFEEIRNLALETLPAMCNVYIPPYCT--IAPVGIFGTN  76
Gli-D2-8        PLSQVCFQQSQQQYPSGQGSFQPSQQNPQAQGSVQPQQLPQFEEIRNLALETLPAMCNVYIPPYCT--IAPVGIFGTN  76
                * :*.. **  :*** .*  *: ** **:*:**.********  * ****:*******:****:*:  :** ******
```
