# Supplementary material for: Production and molecular characterization of bread wheat lines with reduced amount of α-type gliadins
Source: BMC Plant Biol. 2017 Dec 19;17:248. doi: 10.1186/s12870-017-1211-3 (PMC5738072; doi:10.1186/s12870-017-1211-3)
Supplement: Supplementary file 3 — Accession number, molecular weight (MW) and length of the domains in the α-type gliadins isolated from bread wheat cv Pegaso. S=Signal peptide, R1 = Repetitive N-terminal domain; QR1 and QR2 = Polyglutamine domain 1 and 2; NR1 = Unique domain 1; NR2 = C-terminal unique domain. (DOCX 19 kb) [file 12870_2017_1211_MOESM3_ESM.docx]

| **Accession name** | **Accession number** | **Locus** | **MW**  **(kDa)** | **S** | **R1** | **QR1** | **NR1** | **QR2** | **NR2** | **Length**  **(aa)** |
| --- | --- | --- | --- | --- | --- | --- | --- | --- | --- | --- |
| Gli-A2-1 | LT627562 | *Gli-A2* | 30.5 | 20 | 92 | 18 | 69 | 8 | 76 | 283 |
| Gli-A2-2 | LT627563 | *Gli-A2* | 30.5 | 20 | 92 | 18 | 69 | 8 | 76 | 283 |
| Gli-A2-3 | LT627564 | *Gli-A2* | 31.9 | 20 | 92 | 29 | 69 | 8 | 76 | 294 |
| Gli-A2-4 | LT627565 | *Gli-A2* | 30.9 | 20 | 92 | 21 | 69 | 8 | 76 | 286 |
| Gli-A2-5 | LT627566 | *Gli-A2* | 31.9 | 20 | 92 | 29 | 69 | 8 | 76 | 294 |
| Gli-A2-6 | LT627567 | *Gli-A2* | 31.7 | 20 | 92 | 28 | 69 | 8 | 76 | 293 |
| Gli-A2-7 | LT627568 | *Gli-A2* | 31.8 | 20 | 92 | 29 | 69 | 8 | 76 | 294 |
| Gli-A2-8 | LT627569 | *Gli-A2* | 30.8 | 20 | 92 | 21 | 69 | 8 | 76 | 286 |
| Gli-A2-9 | LT627570 | *Gli-A2* | 31.8 | 20 | 92 | 29 | 69 | 8 | 76 | 294 |
| Gli-A2-10 | LT627571 | *Gli-A2* | 31.9 | 20 | 92 | 29 | 69 | 8 | 76 | 294 |
| Gli-A2-11 | LT627572 | *Gli-A2* | 30.4 | 20 | 92 | 18 | 69 | 8 | 76 | 283 |
| Gli-A2-12 | LT627573 | *Gli-A2* | 30.5 | 20 | 92 | 18 | 69 | 8 | 76 | 283 |
| Gli-A2-13 | LT627574 | *Gli-A2* | 30.4 | 20 | 92 | 18 | 69 | 8 | 76 | 283 |
| Gli-A2-14 | LT627575 | *Gli-A2* | 30.4 | 20 | 92 | 18 | 69 | 8 | 76 | 283 |
| Gli-A2-15 | LT627576 | *Gli-A2* | 30.8 | 20 | 92 | 21 | 69 | 8 | 76 | 286 |
| Gli-A2-16 | LT627577 | *Gli-A2* | 30.9 | 20 | 92 | 21 | 69 | 8 | 76 | 286 |
| Gli-A2-17 | LT627578 | *Gli-A2* | 30.7 | 20 | 92 | 20 | 69 | 8 | 76 | 285 |
| Gli-A2-18 | LT627579 | *Gli-A2* | 30.5 | 20 | 92 | 18 | 69 | 8 | 76 | 283 |
| MEAN |  |  | **31.0** | **20** | **92** | **22.4** | **69** | **8** | **76** | **287.4** |
| Gli-D2-1 | LT627592 | *Gli-D2* | 30.0 | 20 | 92 | 15 | 69 | 8 | 78 | 282 |
| Gli-D2-2 | LT627593 | *Gli-D2* | 31.1 | 20 | 92 | 15 | 69 | 16 | 76 | 288 |
| Gli-D2-3 | LT627594 | *Gli-D2* | 30.8 | 20 | 99 | 12 | 69 | 10 | 76 | 286 |
| Gli-D2-4 | LT627595 | *Gli-D2* | 31.1 | 20 | 99 | 17 | 69 | 9 | 76 | 290 |
| Gli-D2-5 | LT627596 | *Gli-D2* | 31.0 | 20 | 99 | 16 | 69 | 9 | 76 | 289 |
| Gli-D2-6 | LT627597 | *Gli-D2* | 31.1 | 20 | 99 | 17 | 69 | 9 | 76 | 290 |
| Gli-D2-7 | LT627598 | *Gli-D2* | 32.2 | 20 | 99 | 16 | 69 | 18 | 76 | 298 |
| Gli-D2-8 | LT627599 | *Gli-D2* | 31.2 | 20 | 99 | 17 | 69 | 9 | 76 | 290 |
| Gli-D2-9 | LT627600 | *Gli-D2* | 31.5 | 20 | 99 | 17 | 69 | 12 | 76 | 293 |
| Gli-D2-10 | LT627601 | *Gli-D2* | 31.4 | 20 | 99 | 17 | 69 | 11 | 76 | 292 |
| Gli-D2-11 | LT627602 | *Gli-D2* | 31.0 | 20 | 99 | 13 | 69 | 12 | 76 | 289 |
| Gli-D2-12 | LT627603 | *Gli-D2* | 33.2 | 20 | 106 | 24 | 69 | 12 | 76 | 307 |
| Gli-D2-13 | LT627604 | *Gli-D2* | 33.5 | 20 | 106 | 25 | 69 | 13 | 76 | 309 |
| Gli-D2-14 | LT627605 | *Gli-D2* | 33.4 | 20 | 106 | 24 | 69 | 13 | 76 | 308 |
| Gli-D2-15 | LT627606 | *Gli-D2* | 33.4 | 20 | 106 | 24 | 69 | 13 | 76 | 308 |
| Gli-D2-16 | LT627607 | *Gli-D2* | 31.5 | 20 | 92 | 18 | 69 | 16 | 76 | 291 |
| Gli-D2-17 | LT627608 | *Gli-D2* | 31.5 | 20 | 92 | 18 | 69 | 16 | 76 | 291 |
| Gli-D2-18 | LT627609 | *Gli-D2* | 31.4 | 20 | 92 | 18 | 69 | 16 | 76 | 291 |
| Gli-D2-19 | LT627610 | *Gli-D2* | 32.2 | 20 | 99 | 17 | 69 | 16 | 76 | 297 |
| MEAN |  |  | **31.7** | **20** | **98.6** | **17.9** | **69** | **12.5** | **76.1** | **294.2** |
| Gli-B2-1 | LT627580 | *Gli-B2* | 30.8 | 20 | 92 | 21 | 69 | 9 | 76 | 287 |
| Gli-B2-2 | LT627581 | *Gli-B2* | 33.8 | 20 | 97 | 22 | 68 | 27 | 78 | 312 |
| Gli-B2-3 | LT627582 | *Gli-B2* | 34.6 | 20 | 91 | 33 | 69 | 27 | 78 | 318 |
| Gli-B2-4 | LT627583 | *Gli-B2* | 34.2 | 20 | 91 | 33 | 69 | 25 | 78 | 316 |
| Gli-B2-5 | LT627584 | *Gli-B2* | 34.5 | 20 | 91 | 33 | 69 | 27 | 78 | 318 |
| Gli-B2-6 | LT627585 | *Gli-B2* | 34.5 | 20 | 91 | 33 | 69 | 27 | 78 | 318 |
| Gli-B2-7 | LT627586 | *Gli-B2* | 34.1 | 20 | 91 | 31 | 69 | 26 | 78 | 315 |
| Gli-B2-8 | LT627587 | *Gli-B2* | 34.5 | 20 | 91 | 33 | 69 | 27 | 78 | 318 |
| Gli-B2-9 | LT627588 | *Gli-B2* | 30.2 | 20 | 55 | 32 | 69 | 27 | 78 | 281 |
| Gli-B2-10 | LT627589 | *Gli-B2* | 34.3 | 20 | 91 | 33 | 69 | 26 | 78 | 317 |
| Gli-B2-11 | LT627590 | *Gli-B2* | 34.7 | 20 | 97 | 22 | 68 | 33 | 78 | 319 |
| Gli-B2-12 | LT627591 | *Gli-B2* | 31.7 | 20 | 97 | 25 | 68 | 7 | 78 | 296 |
| MEAN |  |  | **33.5** | **20** | **89.6** | **29.3** | **68.8** | **24** | **77.8** | **309.6** |
