# Supplementary material for: Production and molecular characterization of bread wheat lines with reduced amount of α-type gliadins
Source: BMC Plant Biol. 2017 Dec 19;17:248. doi: 10.1186/s12870-017-1211-3 (PMC5738072; doi:10.1186/s12870-017-1211-3)
Supplement: Supplementary file 4 — Presence or absence of seven toxic epitopes (α-Glia (31–43): PGQQQPFPPQQPY; α-Glia (31–49): PGQQQPFPPQQPYPQPQPF; α-Glia (31–55): PGQQQPFPPQQPYPQPQPFPSQQPY; α-Glia (44–55): PQPQPFPSQQPY; α-Glia (51–70): SQQPYLQLQPFPQPQLPY; α-Glia (56–75): LQLQPFPQPQLPYPQPQLPY; α-Glia (206–217): LGQGSFRPSQQN) in the 49 deduced amino acidic sequences isolated from the bread wheat cv Pegaso. (DOCX 17 kb) [file 12870_2017_1211_MOESM4_ESM.docx]

| **Accession name** | **Accession number** | **Locus** | **α-Glia (31-43)** | **α-Glia (31-49)** | **α-Glia (31-55)** | **α-Glia (44-55)** | **α-Glia**  **(51-70)** | **α-Glia**  **(56-75)** | **α-Glia**  **(206-217)** |
| --- | --- | --- | --- | --- | --- | --- | --- | --- | --- |
| Gli-A2-1 | LT627562 | *Gli-A2* | - | - | - | - | - | - | + |
| Gli-A2-2 | LT627563 | *Gli-A2* | - | - | - | - | - | - | + |
| Gli-A2-3 | LT627564 | *Gli-A2* | - | - | - | - | - | - | + |
| Gli-A2-4 | LT627565 | *Gli-A2* | - | - | - | + | + | - | + |
| Gli-A2-5 | LT627566 | *Gli-A2* | - | - | - | + | - | - | + |
| Gli-A2-6 | LT627567 | *Gli-A2* | - | - | - | + | - | - | + |
| Gli-A2-7 | LT627568 | *Gli-A2* | - | - | - | + | + | - | + |
| Gli-A2-8 | LT627569 | *Gli-A2* | - | - | - | + | + | - | + |
| Gli-A2-9 | LT627570 | *Gli-A2* | - | - | - | + | - | - | + |
| Gli-A2-10 | LT627571 | *Gli-A2* | - | - | - | + | - | - | + |
| Gli-A2-11 | LT627572 | *Gli-A2* | - | - | - | - | - | - | + |
| Gli-A2-12 | LT627573 | *Gli-A2* | - | - | - | - | - | - | + |
| Gli-A2-13 | LT627574 | *Gli-A2* | - | - | - | + | - | - | + |
| Gli-A2-14 | LT627575 | *Gli-A2* | - | - | - | + | + | - | + |
| Gli-A2-15 | LT627576 | *Gli-A2* | - | - | - | + | - | - | + |
| Gli-A2-16 | LT627577 | *Gli-A2* | - | - | - | + | + | - | + |
| Gli-A2-17 | LT627578 | *Gli-A2* | - | - | - | + | + | - | + |
| Gli-A2-18 | LT627579 | *Gli-A2* | - | - | - | - | - | - | + |
| Gli-D2-1 | LT627592 | *Gli-D2* | - | - | - | - | - | - | - |
| Gli-D2-2 | LT627593 | *Gli-D2* | - | - | - | - | - | - | - |
| Gli-D2-3 | LT627594 | *Gli-D2* | + | + | + | + | + | + | - |
| Gli-D2-4 | LT627595 | *Gli-D2* | + | - | - | - | - | - | - |
| Gli-D2-5 | LT627596 | *Gli-D2* | + | - | - | - | - | - | - |
| Gli-D2-6 | LT627597 | *Gli-D2* | + | - | - | - | - | - | - |
| Gli-D2-7 | LT627598 | *Gli-D2* | + | - | - | - | - | - | - |
| Gli-D2-8 | LT627599 | *Gli-D2* | - | - | - | + | - | - | - |
| Gli-D2-9 | LT627600 | *Gli-D2* | + | - | - | - | - | - | - |
| Gli-D2-10 | LT627601 | *Gli-D2* | + | - | - | - | - | - | - |
| Gli-D2-11 | LT627602 | *Gli-D2* | + | + | + | + | + | - | - |
| Gli-D2-12 | LT627603 | *Gli-D2* | + | + | + | + | + | + | - |
| Gli-D2-13 | LT627604 | *Gli-D2* | + | + | + | + | + | + | - |
| Gli-D2-14 | LT627605 | *Gli-D2* | + | + | + | + | + | + | - |
| Gli-D2-15 | LT627606 | *Gli-D2* | + | + | + | + | + | + | - |
| Gli-D2-16 | LT627607 | *Gli-D2* | + | + | + | + | + | - | - |
| Gli-D2-17 | LT627608 | *Gli-D2* | + | + | + | + | + | - | - |
| Gli-D2-18 | LT627609 | *Gli-D2* | + | + | + | + | + | - | - |
| Gli-D2-19 | LT627610 | *Gli-D2* | + | - | - | - | - | - | - |
| Gli-B2-1 | LT627580 | *Gli-B2* | - | - | - | + | + | - | - |
| Gli-B2-2 | LT627581 | *Gli-B2* | - | - | - | + | - | - | - |
| Gli-B2-3 | LT627582 | *Gli-B2* | - | - | - | + | - | - | - |
| Gli-B2-4 | LT627583 | *Gli-B2* | + | + | - | - | - | - | - |
| Gli-B2-5 | LT627584 | *Gli-B2* | + | + | - | - | - | - | - |
| Gli-B2-6 | LT627585 | *Gli-B2* | + | + | - | - | - | - | - |
| Gli-B2-7 | LT627586 | *Gli-B2* | + | + | - | - | - | - | - |
| Gli-B2-8 | LT627587 | *Gli-B2* | + | + | - | - | - | - | - |
| Gli-B2-9 | LT627588 | *Gli-B2* | + | - | - | - | - | - | - |
| Gli-B2-10 | LT627589 | *Gli-B2* | - | - | - | - | - | - | - |
| Gli-B2-11 | LT627590 | *Gli-B2* | - | - | - | - | - | - | - |
| Gli-B2-12 | LT627591 | *Gli-B2* | - | - | - | - | - | - | - |
